# Crystal Phase Engineering Accelerates Hydrogen Reverse Spillover for Efficient Alkaline Hydrogen Production

**DOI:** 10.1007/s40820-026-02289-3

**Published:** 2026-07-13

**Authors:** Jun Zhang, Xiaoyu Chen, Bin Wu, Xiangyang Guo, Xianlin Qu, Qunzhi Ma, Ying Wang, Jiayi Li, Wei Liu, Xu Li, Liyun Cao, Yi Wang, Jianfeng Huang, Jingxiang Zhao, Fuxiang Zhang, Yongqiang Feng

**Affiliations:** 1 Shaanxi Key Laboratory of Green Preparation and Functionalization for Inorganic Materials, School of Materials Science and Engineering, Shaanxi University of Science and Technology, Xi’an, 710021 People’s Republic of China; 2 College of Chemistry and Chemical Engineering, Harbin Normal University, Harbin, 150025 People’s Republic of China; 3 School of Materials Science and Engineering, Nanyang Technological University, Singapore, 639798 Singapore; 4 State Key Laboratory of Catalysis, Dalian National Laboratory for Clean Energy, Chinese Academy of Sciences, Dalian Institute of Chemical Physics, Dalian, 116023 People’s Republic of China; 5 Center for Microscopy and Analysis, Nanjing University of Aeronautics and Astronautics, Nanjing, 211106 People’s Republic of China

**Keywords:** Phase engineering, Zirconium dioxide, Electrocatalyst, Hydrogen production, Seawater electrolysis

## Abstract

Phase engineering has proved effective in enhancing electrocatalytic performance by precisely controlling crystal structures. While significant efforts have been dedicated to phase regulation of active metals, the role of crystal phase of catalyst supports in steering the hydrogen migration during hydrogen evolution reaction (HER) remains underexplored. This work systematically investigates zirconium dioxide in its monoclinic (*m*-ZrO_2_) and tetragonal (*t*-ZrO_2_) phases as supports for Ru nanoclusters. A combination of spectroscopy and theoretical calculation demonstrates strong electronic metal–support interactions and work function difference between Ru and *m*-ZrO_2_ lead to interfacial charge accumulation, accelerated water dissociation, and reverse hydrogen spillover, significantly enhancing electrocatalytic HER performance in alkaline medium. The Ru@*m*-ZrO_2_/C catalyst exhibits excellent HER activity in different electrolytes. It achieves 10 mA cm^−2^ with an extremely low overpotential (η_10_) of only 28 mV in 1 M KOH, delivering a mass activity of 2.72 A mg_Ru_^−1^ (at 50 mV overpotential), which is 45 times higher than that of commercial Pt/C. The η_10_ values are 56, 42, and 30 mV in 0.5 M H_2_SO_4_, 1 M KOH + 2 M NaCl, and 1 M KOH + simulated seawater, respectively. Furthermore, the catalyst also demonstrates excellent stability under alkaline simulated seawater electrolysis conditions. When integrated into an anion-exchange membrane water electrolyzer, it achieves 1.0 A cm^−2^ at 1.76 V with stability exceeding 300 h. This work underscores the pivotal importance of support phase engineering for designing high-performance electrocatalysts for water splitting.

**Supplementary Information:**

The online version contains supplementary material available at 10.1007/s40820-026-02289-3.

## Highlights

- Phase engineering of ZrO_2_ regulates the electronic metal–support interactions and work function difference between Ru and ZrO_2_, resulting in reverse hydrogen spillover, therefore enhancing electrocatalytic hydrogen evolution reaction in alkaline medium.
- The Ru@*m*-ZrO_2_/C catalyst demonstrated a low overpotential and high mass activity (45 times that of commercial 20% Pt/C), and when integrated into an anion-exchange membrane water electrolyzer, it exhibited stable operation for over 300 hours.

**Supplementary Information:**

The online version contains supplementary material available at 10.1007/s40820-026-02289-3.

## Graphical abstract

**Figure:**
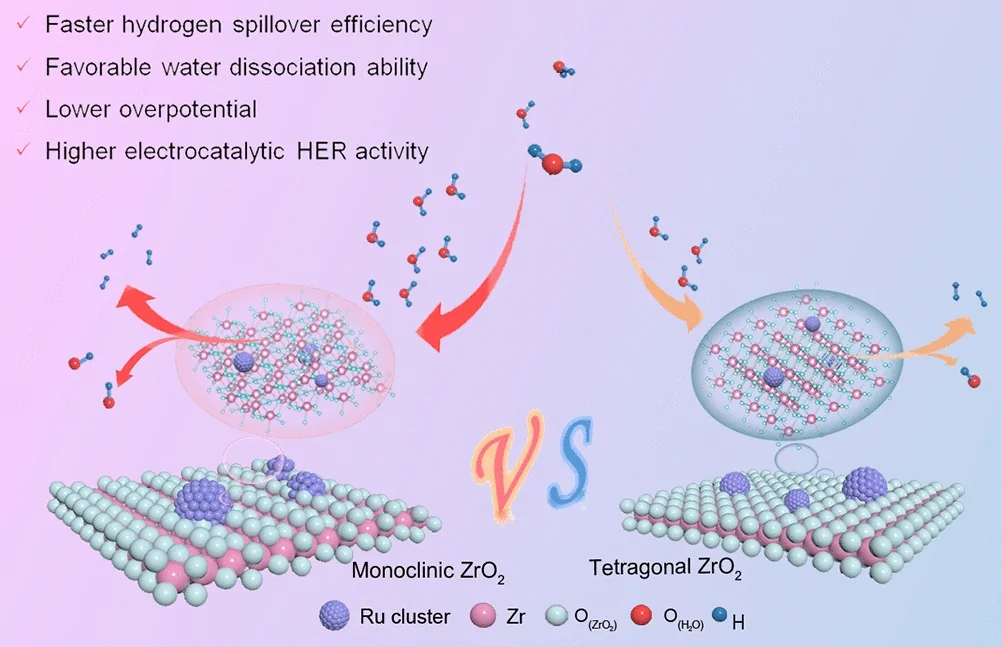

## Introduction

Phase engineering of nanomaterials (PEN) has emerged as a powerful strategy for enhancing electrocatalytic performance by precisely controlling crystal phase structures, thereby offering new pathways toward efficient energy conversion systems, *e.g.*, hydrogen production from water/seawater splitting [1–9], nitrate reduction for ammonia synthesis [10, 11], and alcohol oxidation in fuel cell [12–14]. Up to now, substantial efforts have been devoted to phase regulation of active metals (*e.g.*, Ru, Pt) to optimize their electronic structure and surface adsorption behavior for reactions including the hydrogen evolution reaction (HER), oxygen evolution reaction (OER), and oxygen reduction reaction (ORR), *etc.* [15–22]. For instance, controlled synthesis of face-centered cubic (*fcc*) Ru and hexagonal close-packed (*hcp*) Rh nanostructures has demonstrated remarkable enhancements in activity and stability [23]. However, considerably less attention has been paid to the crystal phase engineering of catalyst supports—a critical factor that governs metal–support interactions and reaction mechanisms such as hydrogen (reverse) spillover.

Electronic metal–support interactions (EMSI) have been leveraged to modulate electronic properties and adsorption energetics at the interfaces, as exemplified in systems such as Pt/MoS_2_ [24] and La_2_Sr_2_PtO_7+δ_ [25]. On the other hand, the work function difference (∆Φ) between metal and support plays a critical role in determining the interfacial charge transfer and hydrogen migration [26, 27]. Several advanced catalyst systems, including Ru-WO_3-x_ [28] and Ir/HfO_2_@C [29], have been designed to exploit hydrogen spillover pathways. It is reported that the large ∆Φ could result in charge accumulation and trap hydrogen species at the interface, thereby facilitating hydrogen reverse spillover (HRS) from the support to metal sites. In contrast, hydrogen spillover is defined as the process by which hydrogen species, after dissociation at the metal sites, migrate to the support [30–33]. However, the role of the support’s crystal phase in regulating EMSI and HRS effects remains underexplored.

Among various support materials, zirconium dioxide (ZrO_2_) exhibits great potential due to its polymorphic nature (monoclinic, tetragonal, cubic, and mixed phases), hydrophilicity, and chemical stability [34–36]. These features make ZrO_2_ an ideal platform for studying the relationship between support crystal phase, metal–support interaction, and hydrogen spillover [37–39]. Although ZrO_2_ has been employed in thermal catalysis and stabilization of single-atom catalysts, its application in electrocatalytic HER in alkaline seawater splitting—particularly with regard to the effect of ZrO_2_ phase on HER activity—has rarely been systematically investigated.

In contrast with conventional EMSI studies that focus on the reconstruction of interfacial electronic structure, the current work highlights the urgent need to advance the fundamental understanding of how the crystal phase of catalyst supports influences the electronic structure of metal centers, modulates metal–support interactions, and directs HRS processes. Specifically, monoclinic ZrO_2_ (*m*-ZrO_2_) and tetragonal ZrO_2_ (*t*-ZrO_2_) supported Ru nanoclusters, defined separately as Ru@*m*-ZrO_2_/C and Ru@*t*-ZrO_2_/C, were successfully synthesized by a two-step solvothermal annealing process. Benefiting from the strong EMSI and large ∆Φ between the surface Ru species and *m*-ZrO_2_ support, the as-synthesized Ru@*m*-ZrO_2_/C exhibits superior HER activity in different media. To deliver a current density of 10 mA cm^−2^, it requires overpotential of 28, 56, 42, and 30 mV in 1 M KOH, 0.5 M H_2_SO_4_, 1 M KOH + 2 M NaCl, and 1 M KOH + simulated seawater, respectively. It also achieves a mass activity as high as 2.72 A mg_Ru_^−1^ at 50 mV overpotential in 1 M KOH, surpassing that of commercial 20% Pt/C by a factor of 45. In situ Raman, electrochemical impedance spectrum (EIS), and density functional theory (DFT) calculation results unveil that the charges accumulated at the Ru/*m*-ZrO_2_ interface not only facilitate the water adsorption and dissociation process at Zr sites, but accelerate the reverse hydrogen spillover from *m*-ZrO_2_ substrate to Ru sites by regulating the *d*-band center of Ru, thereby enhancing the electrocatalytic HER performance. Practically, Ru@*m*-ZrO_2_/C also displays outstanding electrocatalytic activity toward direct seawater electrolysis for hydrogen production with high durability. When assembled in an anion-exchange membrane water electrolyzer (AEMWE) using Ru@*m*-ZrO_2_/C as the cathode and NiFe-layered double hydroxide (NiFe-LDH) as the anode operating at 60 °C in 1 M KOH, a current density of 1.0 A cm^−2^ is achieved at a cell voltage of 1.76 V, which can maintain stable for more than 300 h. This work aims to bridge the gap between phase-controlled support design and HRS-enhanced catalysis, providing a fresh direction for the design of next-generation electrocatalysts for hydrogen production from water splitting.

## Experimental Section

### Materials

Zirconium chloride (ZrCl_4_) was purchased from Shanghai Aladdin Biochemical Technology Co., Ltd. Terephthalic acid (H_2_BDC) was purchased from Saen Chemical Technology (Shanghai) Co., Ltd. Formic acid (HCOOH) was purchased from Tianjin Kemiou Chemical Reagent Co., Ltd. Dimethyl-formamide (DMF) was purchased from Anhui Zesheng Technology Co., Ltd. Methanol (MeOH, CH_3_OH), ethanol (EtOH, CH_3_CH_2_OH), and isopropyl alcohol (IPA, C_3_H_8_O) were purchased from Chemical Reagent Co., Ltd. Ruthenium (III) chloride anhydrous (RuCl_3_), Pt/C (20 wt%), Ru/C (5 wt%), Nafion solution (5%), potassium thiocyanate (KSCN, 99%), ethylenediamine (EDTA, 99%), and potassium hydroxide (KOH, ≧85%) were purchased from Sigma-Aldrich. Sulfuric acid (H_2_SO_4_, 98.5%) was purchased from Codow (Guangdong, China). NaCl, MgCl_2_, MgSO_4_, CaCl_2_, NaHCO_3_, Na_2_SO_4_, and KCl were purchased from Tianjin Tianli Chemical Reagent Co., Ltd. Deionized water (DW, 18.25 MΩ cm^−1^) was obtained from the ultrapure purification system (ULUPURE, UPDR-I-10 T). All the chemicals are analytical grade and used without further purification.

### Preparation of Ru@***m***-ZrO_2_/C, Ru@***t***-ZrO_2_/C, and ZrO_2_/C

#### ***Preparations of Ru@m-ZrO***_***2***_***/C***

Ru@*m*-ZrO_2_/C was synthesized by a two-step method [40, 41]. Firstly, a mixture of ZrCl_4_ (240 mg), H_2_BDC (166.1 mg) and RuCl_3_ (62.1 mg, 0.3 mmol) was dissolved in 55 mL of DMF containing 4 mL of HCOOH in a Teflon-lined autoclave and heated at 120 °C for 12 h. Then, the solid product was collected by washing with DMF and methanol and dried at 60 °C. The obtained product was labeled as Ru@UiO-66. Finally, the Ru@UiO-66 was annealed at 700 °C under Ar for 2 h to obtain Ru@*m*-ZrO_2_/C. The cluster size (0.28, 0.31 and 0.34 mmol) and loading content (0.27, 0.29, 0.32 mmol) of Ru in Ru@*m*-ZrO_2_/C were adjusted by alternating the addition amount of RuCl_3_ while kept other procedures unchanged.

#### ***Preparations of Ru@t-ZrO***_***2***_***/C and ZrO***_***2***_***/C***

Ru@*t*-ZrO_2_/C was synthesized by a three-step method. UiO-66 was synthesized in the same way as Ru@UiO-66, but without the addition of RuCl_3_. Then, 0.3 mmol of RuCl_3_ (62.1 mg) was introduced into a suspension of 200 mg of UiO-66 in 40 mL of methanol and stirred for 24 h. The obtained product, labeled as Ru/UiO-66, was collected by washing with methanol and dried at 60 °C. Finally, the Ru/UiO-66 was annealed at 700 °C under Ar for 2 h to obtain Ru@*t*-ZrO_2_/C. For ZrO_2_/C, the as-synthesized UiO-66 was directly annealed at 700 °C under Ar for 2 h. Similarly, the cluster size (0.28, 0.31, and 0.34 mmol) and loading content (0.29 and 0.32 mmol) of Ru in Ru@*t*-ZrO_2_/C were adjusted by alternating the addition amount of RuCl_3_.

### Material Characterization

X-ray diffraction (XRD) patterns were obtained on a Rigaku D/max-2200PC diffractometer (Japan) with Cu Kα radiation. High-resolution transmission electron microscope (HR-TEM) measurement was conducted on a JEOL JEM-2010F with a 200 kV acceleration voltage. Aberration-corrected high-angle annular dark-field scanning transmission electron microscopy (AC-HAADF-STEM) measurement was conducted on a Thermo Fisher Scientific Spectra 300 operating at 300 kV with cold field emission gun. Raman spectroscopy analysis was performed on a Renishaw-invia microscopic confocal laser Raman spectrometer with a 532-nm laser. Fourier transform infrared spectroscopy (FT-IR) was carried out on a Nicolet-5700 spectrometer in the range of 4000–100 cm^−1^. Scanning electron microscope (SEM) measurement was taken on a Hitachi S-4800 microscope. X-ray photoelectron spectroscopy (XPS) was collected on a Thermo Scientific ESCA Lab 250Xi with Al Kα radiation (200 W). Inductively coupled plasma atomic emission spectroscopy (ICP-AES) measurement was conducted on a SHIMADZU ICPE-90000 instrument. The electrochemical performance was tested on a CHI660E workstation (Chenhua, Shanghai).

### Synchrotron Radiation X-ray Absorption Spectrum Measurement

X-ray absorption near edge structure (XANES) and extended X-ray absorption fine structure (EXAFS) were collected on the BL14W1 beamline in Shanghai Synchrotron Radiation Facility (SSRF). The energy was calibrated by a Ru foil for the Ru K-edge. All data were obtained at room temperature.

### In Situ Electrochemical Raman Measurements

The in situ Raman was performed on a RTS2-DZ-A using a 532-nm laser. During tests, the Ag/AgCl electrode was used as the reference electrode (RE), the platinum wire served as the counter electrode (CE), and the catalyst was loaded on the Ti mesh as the working electrode (WE). Raman spectroscopy was obtained at a voltage of OCP, 0, − 0.005, − 0.010, − 0.015, and − 0.020 V versus reversible hydrogen electrode (RHE) and without voltage. After 100 s of constant voltage stabilization, each spectrum was collected.

### Electrochemical Measurement

All the electrochemical measurements were conducted on a CHI660E electrochemical workstation by a three-electrode system. The 1 M KOH and 0.5 M H_2_SO_4_ electrolyte solutions were prepared from 98% concentrated H_2_SO_4_ and KOH solid and ultrapure water, respectively. The 1 M KOH: *x* M NaCl (*x* = 0, 0.5, 2) electrolyte solution was prepared by KOH and NaCl solid and ultrapure water, and the pH values were 13.99, 13.94, and 13.77 [42], respectively. For the simulated seawater, firstly a mixture of 26.73 g of NaCl, 2.26 g of MgCl_2_, 3.25 g of MgSO_4_, 1.12 g of CaCl_2_, 0.19 g of NaHCO_3_, 3.48 g of Na_2_SO_4,_ and 0.72 g of KCl was dissolved into 1.0 L of ultrapure water [29]. Then, an equal volume of 1 M KOH and the above simulated seawater was mixed and centrifuged to obtain the supernatant as alkaline simulated seawater. The pH value of alkaline simulated seawater was 13.96. A glass carbon electrode (GCE, *ϕ* = 3 mm), a graphitic rod, a Hg/HgO for alkaline and Ag/AgCl for acidic conditions were used as the working, counter- and reference electrode, respectively. To prepare the working electrode: Firstly, 10 mg of the catalyst was dispersed in 195 μL of IPA containing 5 μL of Nafion solution through ultrasonication. Then, 2 μL of the ink was dripped on the surface of GCE and dried at room temperature. All potentials reported in this work were calibrated to the RHE according to the Nernst equation:

E (RHE) = E (Hg/HgO) + 0.924 (1 M KOH).

E (RHE) = E (Ag/AgCl) + 0.281 (0.5 M H_2_SO_4_).

Linear sweep voltammetry (LSV) was performed with compensation at a scan rate of 1 mV s^−1^. Tafel slopes were obtained from the LSV curves according to the equation of *η* = a + b lg j, where *η* refers to the overpotential, b is the Tafel slope, and a denotes the intercept [43]. The electrochemically active surface area (ECSA) and double-layer capacitor (C_dl_) can be obtained in the non-Faradaic region by cyclic voltammetry (CV) scanning with varied scan rate of 2, 4, 6, 8, 10, and 12 mV s^−1^[44]. Faradic efficiency was measured using the drainage method under constant current condition. The electrochemical stability of catalysts was tested by chronoamperometry method at a constant potential. The turnover of frequency (TOF) was estimated by TOF = *I*/2Fn [45], where *I* refers to measured hydrogen current (A), F is Faradaic constant (96,485 C mol^−1^), n is amount of active sites (mol) obtained by the CO-stripping method (n = Q_CO_/2F). The mass activity (MA) is calculated based on the following equation: MA = *I*/*m*, where *I* (A) is the measured current and m (mg) is the mass of Ru loaded on a glassy carbon electrode [29].

### AEMWE Device Test

The anion-exchange membrane (AEM) was prepared by sandwiching the cathodes and anodes on both sides of the membrane (Fumasep FAA-3-PK-130, 110–130 μm thickness). The catalyst of Ru@*m*-ZrO_2_/C loaded on gas diffusion layer (Ru@*m*-ZrO_2_/C/GDL) and NiFe-LDH/GDL (Cropping area: 2.5 × 2.5 cm^2^, active area: 1 × 1 cm^2^, loading: 6.0 mg cm^−2^) were used as cathode and anode, respectively. Subsequently, the AEMWE system was filled with 1.0 M KOH electrolyte, and the flow rate was controlled at 40 mL min^−1^ using a peristaltic pump. Following this, the performance of the AEMWE was assessed with DC power supply. The long-term stability of the device was evaluated using the chronopotentiometry method.

### Theoretical Section

All spin-polarized DFT computations were carried out using the Vienna Ab initio Simulation Package (VASP) [46]. The Perdew–Burke–Ernzerhof (PBE) functional within the generalized gradient approximation (GGA) was employed to describe the exchange–correlation potential [47], using a plane-wave energy cutoff of 450 eV. Consistent with our experimental observations, the (111) and (101) facets were constructed for monoclinic and tetragonal ZrO_2_ materials, denoted as *m*-ZrO_2_ and *t*-ZrO_2_, respectively. To simulate the sub-nanometer Ru particles observed experimentally, we anchored Ru_5_ cluster onto *m*-ZrO_2_ and *t*-ZrO_2_, with the upper two substrate layers fully optimized and the bottom two layers fixed. A vacuum space of 30 Å was applied along the z-direction to minimize interactions between periodic images. Γ-point sampling was performed with a 3 × 3 × 1 k-point grid. The energy convergence criterion was set to 10^−5^ eV, and ionic relaxations were conducted until the maximum force on each atom fell below 0.03 eV Å^−1^. van der Waals interactions were accounted for using the DFT-D3 method, and transition states were located using the climbing image nudged elastic band (CI-NEB) method [48].

To evaluate the catalytic activity toward the HER, the free energy change was computed as ∆G = ∆E + ∆ZPE − T∆S [49, 50], where ∆E represents the reaction energy from the DFT computations. ∆ZPE and T∆S are the energy changes in the zero-point energy and entropy at 298.15, respectively, obtained from vibrational frequency computations and the NIST database for isolated H_2_ and H_2_O. In the HER, the closer the Gibbs free energy of the key intermediate H^*^ (∆G_H*_) is to zero, the better the catalytic performance of the surface catalyst [51, 52].

## Results and Discussion

### Synthesis and Structure Characterization

**Fig. 1:**
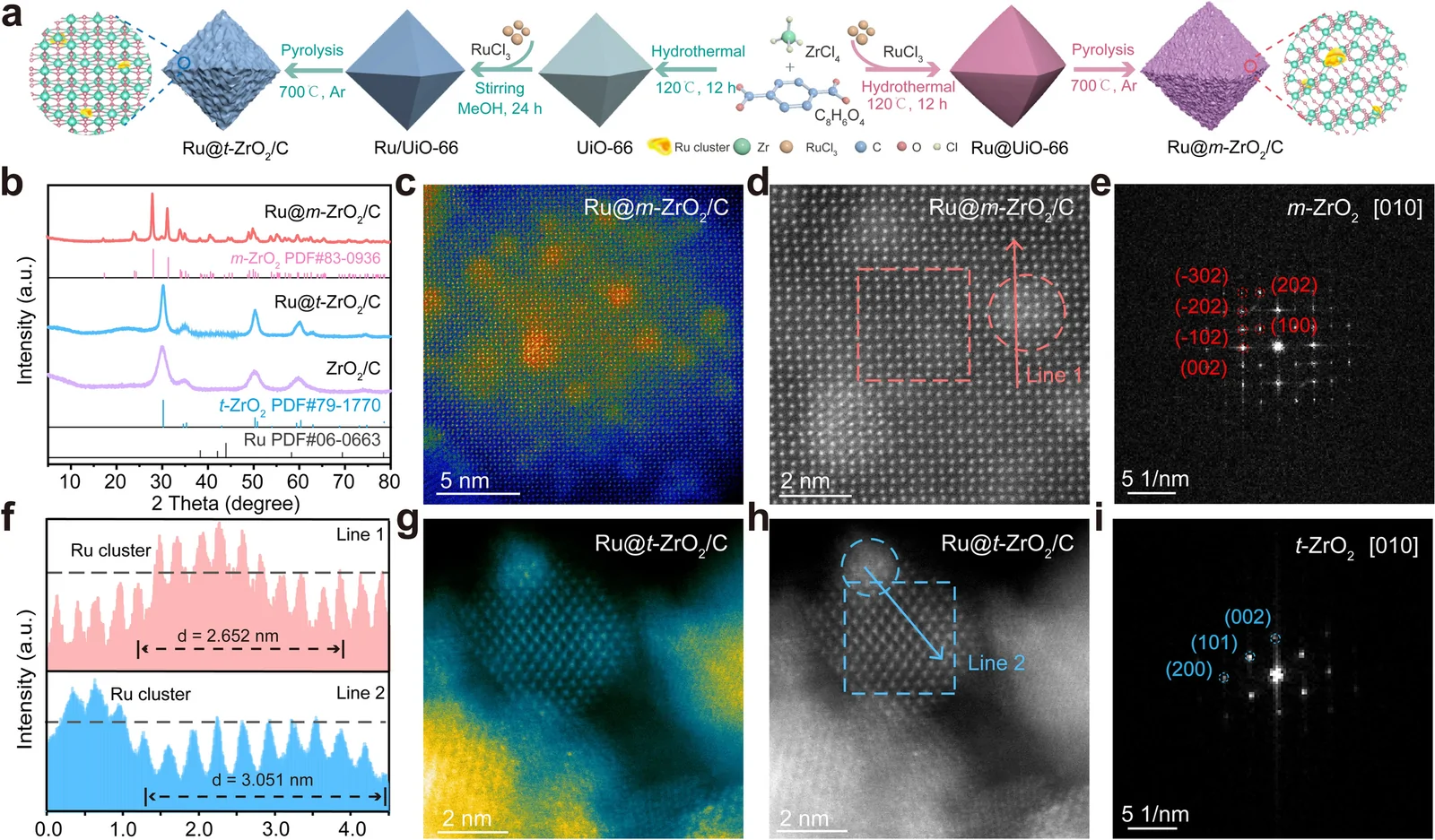
Crystal phase structural characterization. **a** Schematic illustration of the synthetic route of Ru@*m*-ZrO_2_/C and Ru@*t*-ZrO_2_/C. **b** XRD patterns of Ru@*m*-ZrO_2_/C, Ru@*t*-ZrO_2_/C, and ZrO_2_/C. **c** False-color HAADF-STEM image, **d** aberration-corrected HAADF-STEM of Ru@*m*-ZrO_2_/C and **e** the corresponding FFT pattern along the [010] zone axis from the pink dotted square in (**d**). **f** Line intensity profiles of the pink and blue arrows in (**d**) and (**h**) for Ru@*m*-ZrO_2_/C and Ru@*t*-ZrO_2_/C, respectively. **g** False-color HAADF-STEM image, **h** aberration-corrected HAADF-STEM of Ru@*t*-ZrO_2_/C and **i** the corresponding FFT pattern along the [010] zone axis from the blue dotted square in (**h**)

The Ru@*m*-ZrO_2_/C was prepared via a solvothermal annealing process as displayed in Fig. 1a. Typically, RuCl_3_ was introduced into the precursor solution containing H_2_BDC and ZrCl_4_ to form a porous metal–organic framework (defined as Ru@UiO-66, Figs. S1–S4), which was then annealed at 700 °C under Ar atmosphere, converting UiO-66 to monoclinic ZrO_2_ (Figs. S5–S7). In contrast, post-impregnation of RuCl_3_ into pre-synthesized UiO-66 (denoted as Ru/UiO-66) results in reduction to tetragonal ZrO_2_ during annealing, thereby forming the Ru@*t*-ZrO_2_/C counterpart. The crystal phases of Ru@*m*-ZrO_2_/C and Ru@*t*-ZrO_2_/C are first confirmed by XRD. Although the precursor of Ru@UiO-66 and Ru/UiO-66 exhibits similar morphology and structure (Figs. S1–S4), the thermal reduction treatment generates distinct crystalline architectures. As shown in Fig. 1b, a monoclinic ZrO_2_, featuring with 2θ positions at 24.1°, 28.2°, 31.5°, 34.2°, 35.2°, 49.3°, and 50.2°, which are well indexed to the (011), (− 111), (111), (002), (200), (022), and (220) planes of monoclinic zirconia (JCPDS#83-0936), respectively, is observed in Ru@*m*-ZrO_2_/C, while the XRD pattern of Ru@*t*-ZrO_2_/C reassembles the pristine ZrO_2_/C, characterized by a set of diffraction peaks at 30.2°, 35.3°, 50.3°, and 60.2°, corresponding to the (101), (110), (112), and (211) facets of tetragonal zirconia (JCPDS#79-1770), respectively. In both cases, no signal from Ru nanoparticles is detected, indicative of atomic dispersion of Ru species on ZrO_2_ support [53]. The strong adsorption peaks at 742, 584, and 507 cm^−1^ in the FT-IR spectroscopy [54] (Fig. S6) and 185, 331, 375 cm^−1^ in the Raman spectrum [55, 56] (Fig. S7) of Ru@*m*-ZrO_2_/C further demonstrated the existence of *m*-ZrO_2_.

The morphology and structure of Ru@*m*-ZrO_2_/C and Ru@*t*-ZrO_2_/C are then investigated by TEM. The high-resolution TEM, high-angle annular dark-field scanning transmission electron microscopy (HAADF-STEM) images (Figs. S8–S13) and corresponding HAADF image with false color (Fig. 1c, g) show that some bright nanoclusters with diameter of 1–2 nm are anchored on the large nanoparticles and dispersed on the carbon substrate in Ru@*m*-ZrO_2_/C and Ru@*t*-ZrO_2_/C. Aberration-corrected HAADF-STEM further reveals the lattice distance of 0.265 and 0.305 nm assigned to the (002) facet of *m*-ZrO_2_ and (101) of *t*-ZrO_2_ (Fig. 1d, f, h), respectively. The slightly larger crystal spacing of ZrO_2_ substrate in Ru@*m*-ZrO_2_/C and Ru@*t*-ZrO_2_/C with respect to the parent *m*-ZrO_2_ and *t*-ZrO_2_ could be caused by the formation of Ru–O–Zr connection after loading of Ru clusters. The corresponding fast Fourier transform (FFT) patterns in the square region marked by pink and blue dotted lines for Ru@*m*-ZrO_2_/C (Fig. 1e) and Ru@*t*-ZrO_2_/C (Fig. 1i) confirm the ZrO_2_ along [010] zone axes, respectively [57]. The line intensity profiles at the pink and blue arrows separately for Ru@*m*-ZrO_2_/C and Ru@*t*-ZrO_2_/C ambiguously demonstrate the successful incorporation of Ru cluster on the ZrO_2_ matrix (Fig. 1f). The corresponding energy-dispersive X-ray spectroscopy (EDS) mapping reveals the homogeneous distribution of Zr, O, and C elements and the Ru clusters on the ZrO_2_ support (Figs. S12a and S14). Based on the ICP-AES, the Ru content in Ru@*m*-ZrO_2_/C and Ru@*t*-ZrO_2_/C is 0.72 and 1.02 wt%, respectively (Table S1).

**Fig. 2:**
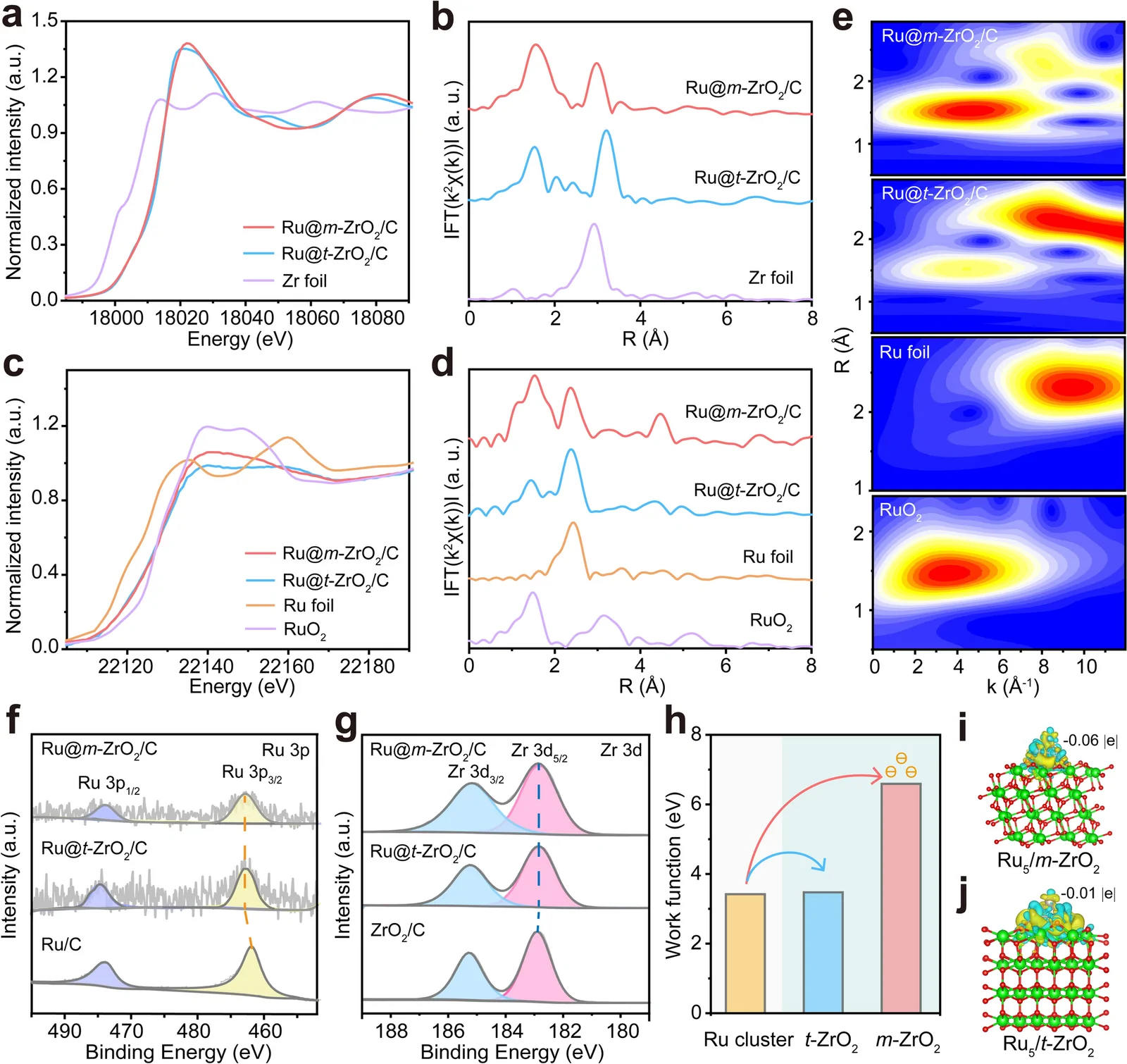
Electronic and coordination structure characterization. **a** Zr K-edge XANES spectra and **b** FT-EXAFS spectra of Ru@*m*-ZrO_2_/C, Ru@*t*-ZrO_2_/C and Zr foil. **c** Ru K-edge XANES spectra, **d** FT-EXAFS spectra, and **e** the corresponding wavelet transform images of Ru@*m*-ZrO_2_/C, Ru@*t*-ZrO_2_/C, Ru foil and RuO_2_. **f** Ru 3*p* XPS profiles of Ru@*m*-ZrO_2_/C, Ru@*t*-ZrO_2_/C and Ru/C. **g** Zr 3*d* XPS profiles of Ru@*m*-ZrO_2_/C, Ru@*t*-ZrO_2_/C and ZrO_2_/C. **h** Work function of Ru cluster, *m*-ZrO_2_ and *t*-ZrO_2_. Charge density difference images of **i** Ru@*m*-ZrO_2_/C, and **j** Ru@*t*-ZrO_2_/C. Color code for the balls: Zr, green; O, red; Ru, cyan. The blue and yellow contour part represents charge depletion and accumulation, respectively

The chemical valence state and local coordination structure of ZrO_2_ support and Ru species in Ru@*m*-ZrO_2_/C and Ru@*t*-ZrO_2_/C are unveiled by XAS and XPS. As shown in Figs. 2a and S15, the typical feature at 18,014 eV in the Zr K-edge XANES spectroscopy proves the Zr^4+^ valence state in Ru@*m*-ZrO_2_/C and Ru@*t*-ZrO_2_/C [58]. Fourier-transformed extended X-ray absorption fine structure (FT-EXAFS) spectroscopy (Fig. 2b) and fitting curves (Fig. S16) disclosed two distinctive geometry of ZrO_2_ in Ru@*m*-ZrO_2_/C and Ru@*t*-ZrO_2_/C [59, 60]. For the former, the peak at 1.58 and 3.0 Å is assigned to Zr–O and Zr–Zr shell with coordination number (CN) of 8 and 6 (Table S2), respectively [58], while the Zr–O and Zr–Zr scattering in Ru@*t*-ZrO_2_/C was located separately at 1.50 and 3.2 Å with CN of 4 and 12, respectively [61]. Such coordination environment divergence is also reflected by the wavelet transform (WT) of EXAFS at Zr K-edge for Ru@*m*-ZrO_2_/C and Ru@*t*-ZrO_2_/C (Fig. S17). Figure 2c illustrates the Ru K-edge XANES, of which the adsorption edge of Ru@*m*-ZrO_2_/C and Ru@*t*-ZrO_2_/C locates between those of Ru foil and RuO_2_ references, indicative of valence state of Ru in the range of 0 ~ 4. The first derivative XANES spectra further demonstrate a relatively higher valence state of Ru in Ru@*m*-ZrO_2_/C with respect to Ru@*t*-ZrO_2_/C (Fig. S18). The FT-EXAFS (Fig. 2d) and fitting result (Fig. S19 and Table S3) reveals the formation of Ru–O and Ru–Ru bond in Ru@*m*-ZrO_2_/C with CN of 2.7 and 2.5, respectively, which is further confirmed by WT-EXAFS at Ru K-edge for Ru@*m*-ZrO_2_/C and Ru@*t*-ZrO_2_/C (Fig. 2e).

The electronic structure of Ru@*m*-ZrO_2_/C and Ru@*t*-ZrO_2_/C was also revealed by XPS. The Ru 3*p* XPS spectrum (462.87 eV for Ru 3*p*_3/2_ and 483.97 eV for Ru 3*p*_1/2_) of Ru@*m*-ZrO_2_/C and Ru@*t*-ZrO_2_/C displayed a upshift to higher binding energy (BE) with respect to Ru/C (Figs. 2f and S20–S22), while the BEs for Zr 3*d* down-shift relative to ZrO_2_/C (Fig. 2g). The result indicates that the electrons transfer from Ru sites to ZrO_2_ substrate via Ru–O–Zr path, which was further confirmed by the work function (WF) and charge density difference (CDD) calculation. As shown in Fig. 2h, the *m*-ZrO_2_ possesses a much larger WF (6.59 eV) than those of *t*-ZrO_2_ (3.47 eV) and Ru cluster (3.42 eV). The larger ∆Φ between Ru cluster and *m*-ZrO_2_ substrate could drive electrons accumulation at the Ru/*m*-ZrO_2_ interface, facilitating the adsorption of water molecules. CDD diagrams and Bader charge analysis (Fig. 2i, j) further illustrate more charge depletion from the Ru_5_ cluster to *m*-ZrO_2_ (0.06 e) with respect to *t*-ZrO_2_ (0.01 e).

### Electrocatalytic HER Performance

**Fig. 3:**
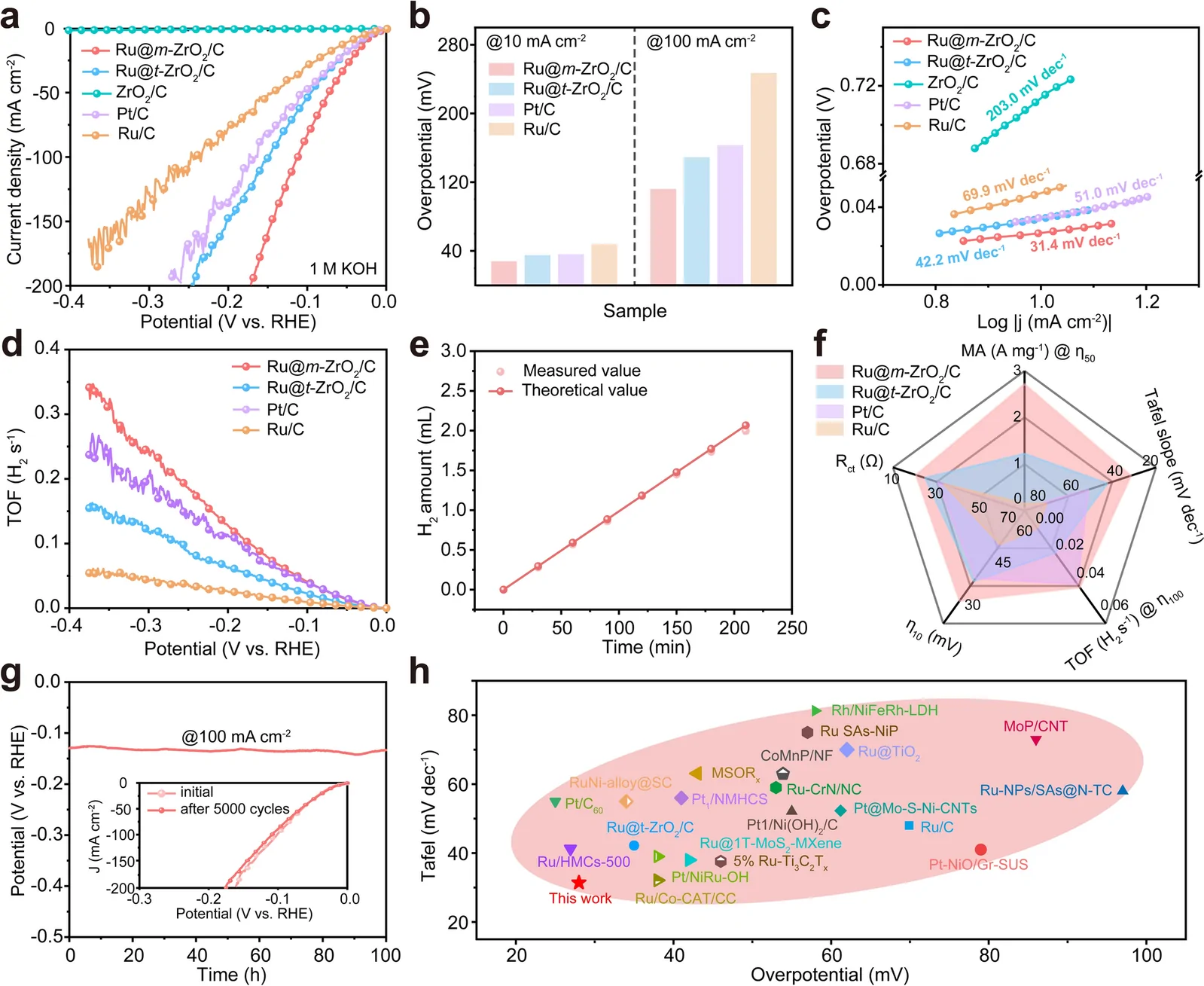
Electrocatalytic HER performance in 1 M KOH. **a** LSV curves, **b** overpotentials at 10 and 100 mA cm^−2^, **c** Tafel slopes, and **d** TOF values for Ru@*m*-ZrO_2_/C, Ru@*t*-ZrO_2_/C, Pt/C and Ru/C. **e** Faradaic efficiency of Ru@*m*-ZrO_2_/C. **f** Comparison of MA, R_ct_, η_10_, TOF and Tafel slope for Ru@*m*-ZrO_2_/C, Ru@*t*-ZrO_2_/C, Pt/C and Ru/C. **g** Chronopotentiometry curve of Ru@*m*-ZrO_2_/C at 100 mA cm^−2^, inset showing the LSV curve before and after 5000 cycles of CV scanning. **h** Comparison of HER performance of Ru@*m*-ZrO_2_/C with the recently-reported HER electrocatalysts

**Fig. 4:**
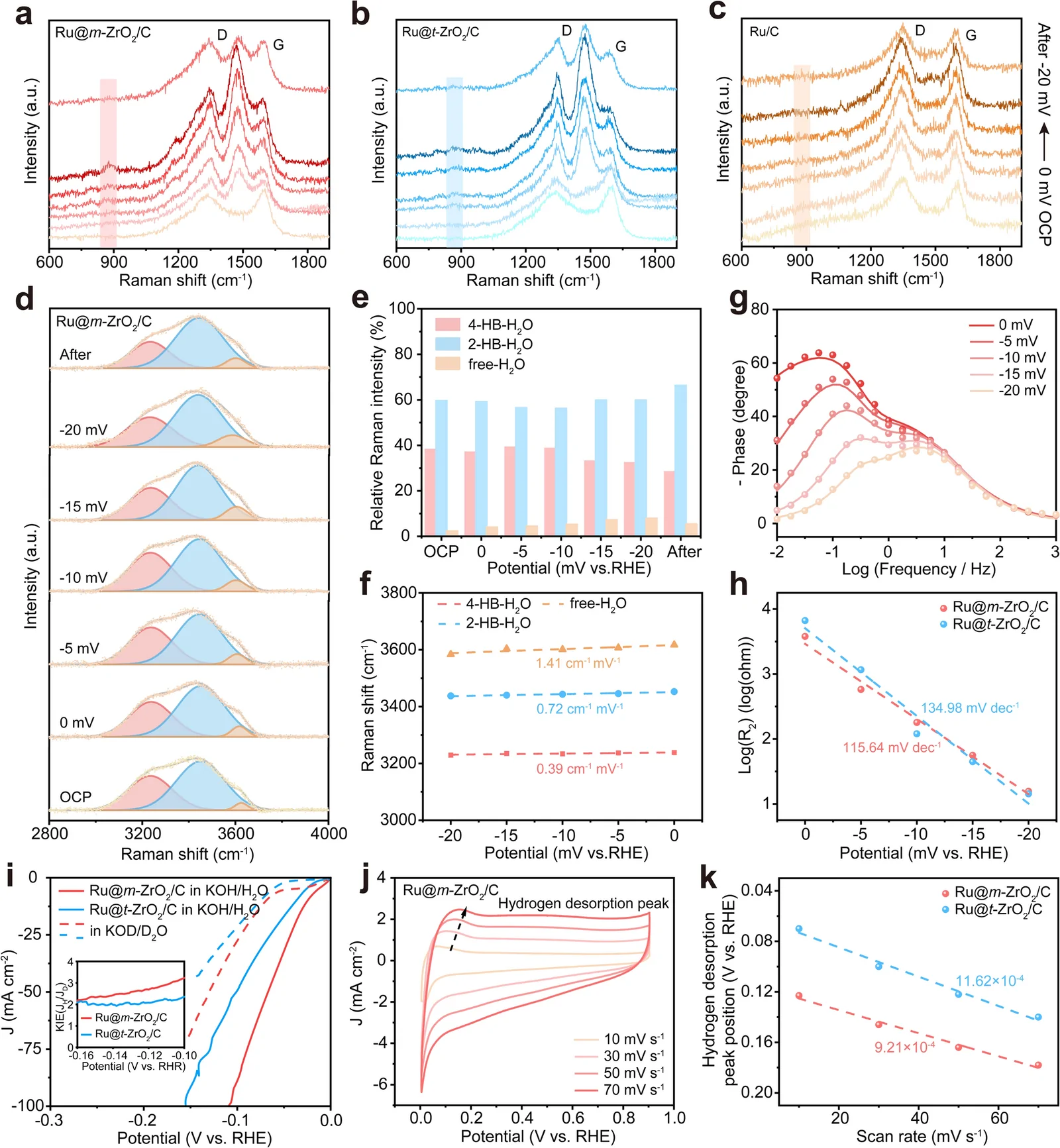
Experimental insights on HER mechanism. In situ Raman spectra of **a** Ru@*m*-ZrO_2_/C, **b** Ru@*t*-ZrO_2_/C and **c** Ru/C in KOD/D_2_O. **d** In situ Raman spectra of Ru@*m*-ZrO_2_/C in the range of 2800–4000 cm^−1^. **e** Population of 4-HB·H_2_O, 2-HB·H_2_O and free H_2_O, and **f** Stark slope derived from Raman spectra at different applied potential in (**d**). **g** Bode plots of Ru@*m*-ZrO_2_/C at varied potentials, **h** Tafel slope of Ru@*m*-ZrO_2_/C and Ru@*t*-ZrO_2_/C estimated from R_2_. **i** Polarization curves of Ru@*m*-ZrO_2_/C and Ru@*t*-ZrO_2_/C in 1 M KOH/H_2_O (solid lines) and KOD/D_2_O (dashed lines), inset showing the KIE value against potential. **j** CV curves of Ru@*m*-ZrO_2_/C. **k** Plots of hydrogen desorption peak position versus scan rate for Ru@*m*-ZrO_2_/C and Ru@*t*-ZrO_2_/C

The HER catalytic performance of Ru@*m*-ZrO_2_/C and Ru@*t*-ZrO_2_/C was evaluated in a three-electrode system with 1 M KOH electrolyte. For comparison, Pt/C, Ru/C, and ZrO_2_/C were selected as references. As shown in Fig. 3a, b, Ru@*m*-ZrO_2_/C exhibits the lowest overpotential, requiring only 28 mV to reach 10 mA cm^−2^, outperforming Ru@*t*-ZrO_2_/C (35 mV), Pt/C (36 mV), Ru/C (48 mV), and ZrO_2_/C (716 mV). The corresponding Tafel slopes (Fig. 3c) further reveal the HER kinetics, with values of 31.4, 42.2, 51.0, 69.9, and 203.0 mV dec^−1^ for Ru@*m*-ZrO_2_/C, Ru@*t*-ZrO_2_/C, Pt/C, Ru/C, and ZrO_2_/C, respectively, indicating superior reaction kinetics for Ru@*m*-ZrO_2_/C [62, 63]. Furthermore, control experiments demonstrate the cluster size and dispersion of Ru clusters in Ru@*m*-ZrO_2_/C and Ru@*m*-ZrO_2_/C have negligible effect on the catalytic HER performance (Figs. S23–S26 and Tables S4 and S5). To probe the intrinsic activity, TOF was measured based on CO-stripping experiment [45]. The results (Figs. 4d and S27, Table S6) confirm that Ru@*m*-ZrO_2_/C exhibits a high TOF value. Mass activity (MA) of Ru@*m*-ZrO_2_/C at η_50_ was as large as 2.72 A mg_Ru_^−1^ (Fig. S28), 45-fold higher than that of commercial 20% Pt/C. ECSA estimated via C_dl_ from CV measurements (Fig. S29) [64] is highest for Ru@*m*-ZrO_2_/C at 37.6 mF cm^−2^, compared to 20.2 mF cm^−2^ for Ru@*t*-ZrO_2_/C, 16.6 mF cm^−2^ for Pt/C, and 4.7 mF cm^−2^ for ZrO_2_/C. Moreover, the charge transfer resistance (*R*_ct_) is derived from EIS data (Fig. S30) [65], of which Ru@*m*-ZrO_2_/C exhibits a smallest *R*_ct_ value, indicative of favorable charge transfer kinetics of Ru@*m*-ZrO_2_/C. In addition, the Faradaic efficiency of Ru@*m*-ZrO_2_/C was measured to be approximately 96.8% (Fig. 3e). The HER parameters of Ru@*m*-ZrO_2_/C are comparably listed in Fig. 3f and Table S7, evidently confirming the superior performance among all the samples. To elucidate the nature of active sites, poisoning experiments using EDTA and KSCN are conducted (Fig. S31). The significant decrease in current density upon KSCN addition—contrasting with negligible change after EDTA treatment—suggests that Ru species primarily exist as clusters, which are critical for HER activity [66]. The Ru@*m*-ZrO_2_/C catalyst also exhibited exceptional stability in alkaline medium, maintaining performance over 100 h at 100 mA cm^−2^ (Fig. 3g). Moreover, after 5000 cycles of continuous CV scanning, the LSV curve of Ru@*m*-ZrO_2_/C almost overlapped with the initial one, further demonstrating the robust durability. Notably, Ru@*m*-ZrO_2_/C also demonstrated enhanced activity in acidic medium, requiring a *η*_10_ of 56 mV, a Tafel slope of 49.4 mV dec^−1^, and a *C*_dl_ value of 30.3 mF cm^−2^ (Figs. S32 and S33). Moreover, post-test analyses via XRD, Raman, SEM, and XPS confirmed the structural and chemical stability of Ru@*m*-ZrO_2_/C (Figs. S34–S39). In addition, the HER performance of Ru@*m*-ZrO_2_/C surpasses many reported electrocatalysts (Fig. 3h, Tables S8 and S9).

### Experimental Evidence for Hydrogen Reverse Spillover

To elucidate the underlying mechanism for the enhanced HER activity of Ru@*m*-ZrO_2_/C, various in situ/operando measurements were employed. Firstly, in situ Raman spectra of Ru@*m*-ZrO_2_/C, Ru@*t*-ZrO_2_/C, ZrO_2_/C, and Ru/C were collected between 0 and − 0.02 V versus RHE (Figs. 4a–c and S40). At open-circuit potential (OCP), all samples only exhibit the typical D and G bands of carbon, which remained almost unchanged with decreasing potential, indicating that the carbon support does not participate in hydrogen migration [67]. Notably, as the potential becomes more negative, the Raman peaks of Ru@*m*-ZrO_2_/C at 1118 and 1504 cm^−1^ intensified more significantly than those of Ru@*t*-ZrO_2_/C, while the corresponding signals for ZrO_2_/C are rather weak and almost undetectable for Ru/C. To assign these peaks, the electrolyte was switched from KOH/H_2_O to KOD/D_2_O (Figs. 4a–c and S41). The peak at 1504 cm^−1^ blue-shifts to 1468 cm^−1^, and the peak at 1118 cm^−1^ red-shifts to 1183 cm^−1^ due to the H–D isotope effect, confirming that both peaks originate from hydrogen atom vibrations. These shifts allow assignment of the 1118 and 1504 cm^−1^ bands to Zr–OH species at the Ru/ZrO_2_ interface. Under D_2_O conditions, slower O–D bond cleavage leads to accumulation of detectable Ru-D species at ~ 876 cm^−1^, providing clear HRS evidence of H* migration from ZrO_2_ support to Ru sites. Compared with Ru@*t*-ZrO_2_/C, Ru@*m*-ZrO_2_/C exhibits a stronger Ru–D signal, further demonstrating the enhanced OH adsorption capacity and superior water dissociation ability of the *m*-ZrO_2_ support. Additionally, CO-stripping measurements were conducted to evaluate OH adsorption ability of Ru@*m*-ZrO_2_/C (Fig. S42), as CO oxidation requires reactive OH* species [68]. In CO-saturated 1 M KOH electrolyte, the onset potential for CO oxidation over Ru@*m*-ZrO_2_/C is negatively shifted by ∼73 mV compared to Ru@*t*-ZrO_2_/C, indicating more facile OH adsorption on the Ru@*m*-ZrO_2_/C surface due to strong Ru/*m*-ZrO_2_ EMSI effect.

Furthermore, the EMSI effect enables to induce interfacial electric field polarization, which is critical in regulation of hydrogen-bond network rearrangement within the electric double layer (EDL). Using Gaussian fitting, the broad peak between 3000 and 3800 cm^−1^ in the Raman spectra of Ru@*m*-ZrO_2_/C and Ru@*t*-ZrO_2_/C (Fig. 4d), attributed to interfacial water vibrations, could be deconvoluted into three components: four-coordinated hydrogen-bonded water (4-HB·H_2_O), two-coordinated hydrogen-bonded water (2-HB·H_2_O), and free H_2_O [69]. As depicted in Fig. 4e, the proportion of free H_2_O in Ru@*m*-ZrO_2_/C is slightly higher than that in Ru@*t*-ZrO_2_/C and increases rapidly with decreased potential (Figs. S43 and S44), indicating the interfacial free H_2_O in the EDL is critical for the hydrogen migration, contributing to the faster water dissociation kinetics over Ru@*m*-ZrO_2_/C during HER [70, 71]. The trend for the vibrational frequency change along with the applied potential is attributed to the vibrational Stark effect [27, 72]. As shown in Fig. 4f, the Stark slope of free H_2_O (1.41 cm^−1^ mV^−1^) on Ru@*m*-ZrO_2_/C is higher than those of 4-HB·H_2_O (0.39 cm^−1^ mV^−1^) and 2-HB·H_2_O (0.72 cm^−1^ mV^−1^) and also higher than that of Ru@*t*-ZrO_2_/C (Fig. S43), indicating that free water on Ru@*m*-ZrO_2_/C is more sensitive to the electrode’s local electric field.

The hydrogen adsorption/desorption kinetics during water dissociation process were subsequently analyzed via in situ EIS measurement [67, 73]. The HER equivalent circuit model consists of three components (Fig. S45): R_s_ represents the uncompensated solution resistance, while CPE_1_ and R_1_ correspond to the capacitance and resistance of hydrogen adsorption at low frequencies (Volmer step), and CPE_2_ and R_2_ represent hydrogen adsorption behavior on the catalyst surface at high frequencies (Heyrovsky step). Nyquist plots of Ru@*m*-ZrO_2_/C and Ru@*t*-ZrO_2_/C obtained at various overpotentials in alkaline medium are shown in Fig. S46 and Tables S10, S11. The corresponding Bode plots in Fig. 4g show two distinct phase angles, confirming that the HER process follows the Volmer–Heyrovsky mechanism. The rapid decrease in phase angles in the low-frequency region suggests fast water dissociation kinetics [74]. The hydrogen adsorption kinetics were quantified by fitting the logarithmic hydrogen adsorption resistance Log(R_2_) against overpotential [26]. As illustrated in Fig. 4h, Ru@*m*-ZrO_2_/C exhibited a lower Tafel slope (115.64 mV dec^−1^) than Ru@*t*-ZrO_2_/C (134.98 mV dec^−1^), indicating accelerated hydrogen adsorption and transfer over *m*-ZrO_2_. The H/D kinetic isotope effect (KIE) is used to probe hydrogen/proton transfer kinetics [75]. Polarization curves revealed significantly lower HER activities for both catalysts in 1 M KOD/D_2_O compared to 1 M KOH/H_2_O (Fig. 4i), with KIE values exceeding 2, confirming the involvement of hydrogen/proton transfer in the HER process. Hydrogen desorption kinetics were further evaluated via CV measurement. By analyzing the shift in hydrogen desorption peak positions with varying scan rates (Figs. 4j, k and S47), the desorption kinetics were quantified [25]. The slope for Ru@*m*-ZrO_2_/C (9.21 × 10^−4^) is lower than that for Ru@*t*-ZrO_2_/C (11.62 × 10^−4^) in alkaline electrolyte, indicating faster hydrogen desorption on Ru@*m*-ZrO_2_/C. In situ EIS and CV measurements under acidic conditions also demonstrated superior hydrogen adsorption/desorption kinetics for Ru@*m*-ZrO_2_/C (Figs. S48–S51 and Tables S12 and S13).

### Theoretical Insight into the Electrocatalytic Mechanism

**Fig. 5:**
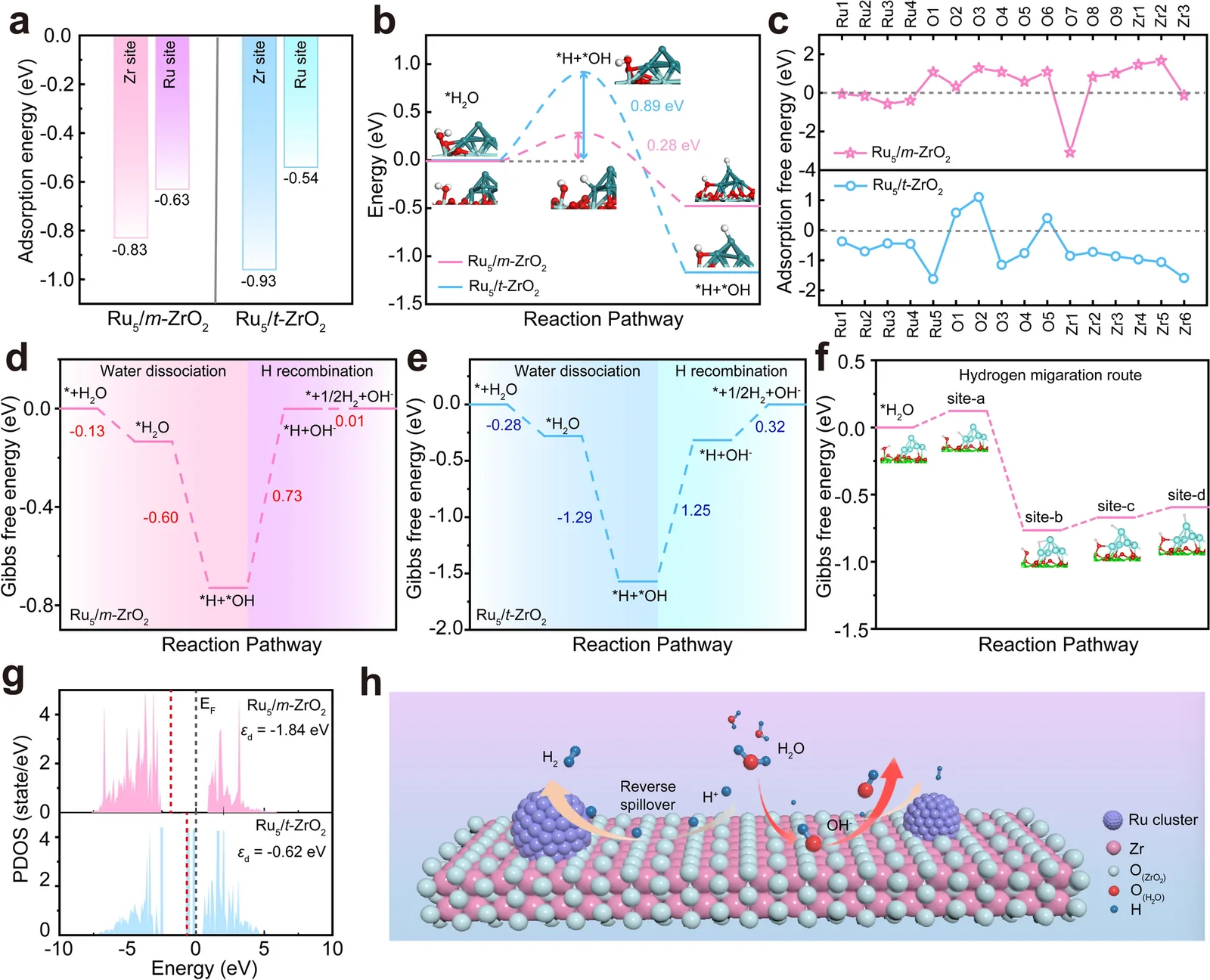
Theoretical calculation for HER mechanism. **a** Calculated *H_2_O adsorption energy on Zr and Ru sites in Ru_5_/*m*-ZrO_2_ and Ru_5_/*t*-ZrO_2_. **b** Kinetic energy barrier of *H_2_O dissociation on Ru_5_/*m*-ZrO_2_ and Ru_5_/*t*-ZrO_2_. **c** Hydrogen adsorption free energy (ΔG_H*_) at Ru, Zr and O sites in Ru_5_/*m*-ZrO_2_ and Ru_5_/*t*-ZrO_2_. Gibbs free energy diagram for HER on **d** Ru_5_/*m*-ZrO_2_ and **e** Ru_5_/*t*-ZrO_2_. **f** Hydrogen migration energy on Ru_5_/*m*-ZrO_2_. **g** PDOS profiles of Ru *d* orbital for Ru_5_/*m*-ZrO_2_ and Ru_5_/*t*-ZrO_2_. **h** Schematic illustration of the HER mechanism

To further elucidate the catalytic mechanism underlying the excellent HER activity of sub-nanometer Ru clusters anchored on ZrO_2_ surfaces under alkaline conditions, DFT calculations were then performed. A Ru_5_ cluster bonded on *m*-ZrO_2_ (Ru_5_/*m*-ZrO_2_) and *t*-ZrO_2_ (Ru_5_/*t*-ZrO_2_) was constructed to represent Ru@*m*-ZrO_2_/C and Ru@*t*-ZrO_2_/C catalyst (Fig. S52), respectively. The binding energies of Ru_5_ clusters on the *m*-ZrO_2_ and *t*-ZrO_2_ surfaces were calculated separately to be − 6.75 and − 11.42 eV (Fig. S53), indicating favorable thermodynamic stability. In alkaline HER, the cleavage of the H–OH bond in H_2_O is inherently sluggish, limiting the overall reaction rate [76, 77]. To address this, we investigated H_2_O adsorption and subsequent dissociation at various sites on both catalysts, including Ru atoms within the Ru_5_ clusters and neighboring Zr sites. Notably, the Zr atoms adjacent to the Ru_5_ clusters exhibited stronger H_2_O adsorption (Fig. 5a), as indicated by more negative adsorption energies (− 0.83 and − 0.96 eV), compared to the Ru sites (− 0.63 and − 0.54 eV), which can be attributed to the *d*-band center (*ε*_*d*_) of the Zr sites being closer to the Fermi level than that of the Ru sites according to the projected density of state (PDOS) result (Fig. S54). We further examined the subsequent dissociation of adsorbed H_2_O into *H and *OH on Zr sites (Fig. 5b). The results show that the energy barrier for water dissociation on the Ru_5_/*m*-ZrO_2_ catalyst is 0.28 eV, which is significantly lower than the barrier on Ru_5_/*t*-ZrO_2_ (0.89 eV), indicating that Ru_5_/*m*-ZrO_2_ possesses markedly enhanced catalytic activity for the cleavage of water molecules. Under realistic alkaline conditions where Zr sites undergo surface hydroxylation, we further evaluated water adsorption and dissociation at the hydroxylated Ru_5_/*m*-ZrO_2_ interface. Compared to the non-hydroxylated surface, the hydroxylated surface exhibits enhanced water adsorption at the Zr site, with an adsorption energy of − 2.35 eV, and a lower dissociation barrier of 0.13 eV (Fig. S55). Thus, under operating conditions, the Zr site remains active, and its catalytic contribution is maintained or even enhanced.

In the HER process, the Gibbs free energy of H* adsorption (ΔG_H*_) is commonly recognized as a key descriptor of a catalyst’s hydrogen evolution activity [78]. Accordingly, we computed ΔG_H*_ for the Ru_5_/*m*-ZrO_2_ and Ru_5_/*t*-ZrO_2_ catalysts, considering various adsorption sites, including each Ru atom within the Ru_5_ cluster as well as Zr and O sites on the substrate (Fig. S56). Notably, our DFT results show that the ΔG_H*_ at the top Ru site (Ru1) of the Ru_5_/*m*-ZrO_2_ catalyst was calculated to be − 0.06 eV (Fig. 5c), which is significantly lower than the lowest ΔG_H*_ value on Ru_5_/*t*-ZrO_2_ (-0.38 eV), thermodynamically confirming that Ru_5_ anchored on *m*-ZrO_2_ exhibits superior HER performance. An in-depth inspection on the reaction pathway reveals that the departure of OH* on *m*-ZrO_2_ as the potential-determining step (PDS) possesses a much lower free energy barrier (0.73 eV, Fig. 5d) with respect to *t*-ZrO_2_ (1.25 eV, Fig. 5e). Moreover, the generated active H* species preferentially spill over from the *m*-ZrO_2_ support to the anchored Ru_5_ cluster with a smaller thermodynamic barrier of 0.60 eV corresponding to an exergonic energy of 0.47 eV compared to *t*-ZrO_2_ (thermodynamic barrier of 1.29 eV and exergonic energy of 1.17 eV), favoring the HRS process (Fig. 5f). The pronounced difference in H* adsorption strength between the Ru_5_ clusters supported on *m*-ZrO_2_ and *t*-ZrO_2_ can be attributed to variations in the *ε*_*d*_ of the Ru active sites (Fig. 5g). Specifically, the *ε*_*d*_ of the Ru sites on the *m*-ZrO_2_ surface lies further from the Fermi level (− 1.84 eV) than that on the *t*-ZrO_2_ support (− 0.62 eV), resulting in weaker H^*^ adsorption and bringing it closer to the ideal ΔG_H*_ of 0 eV for optimal HER performance. Therefore, based on the above theoretical computations, we propose that the origin of the exceptional HER performance of Ru_5_ clusters anchored on ZrO_2_ lies in the differing work functions of the Ru_5_ clusters and the ZrO_2_ substrate (Fig. 2h), which leads to a significant accumulation of electrons at their interface and holds great promise for accelerating the OH* departure and hydrogen spillover process. Among the two materials, the Ru_5_/*m*-ZrO_2_ catalyst exhibits enhanced capability in facilitating the dissociation of adsorbed H_2_O molecules into OH* and H*, with a lower energy barrier of 0.28 eV, thereby promoting the spillover of H^*^ species from the *m*-ZrO_2_ support to the Ru cluster (Fig. 5h).

### Practical Potential Application

**Fig. 6:**
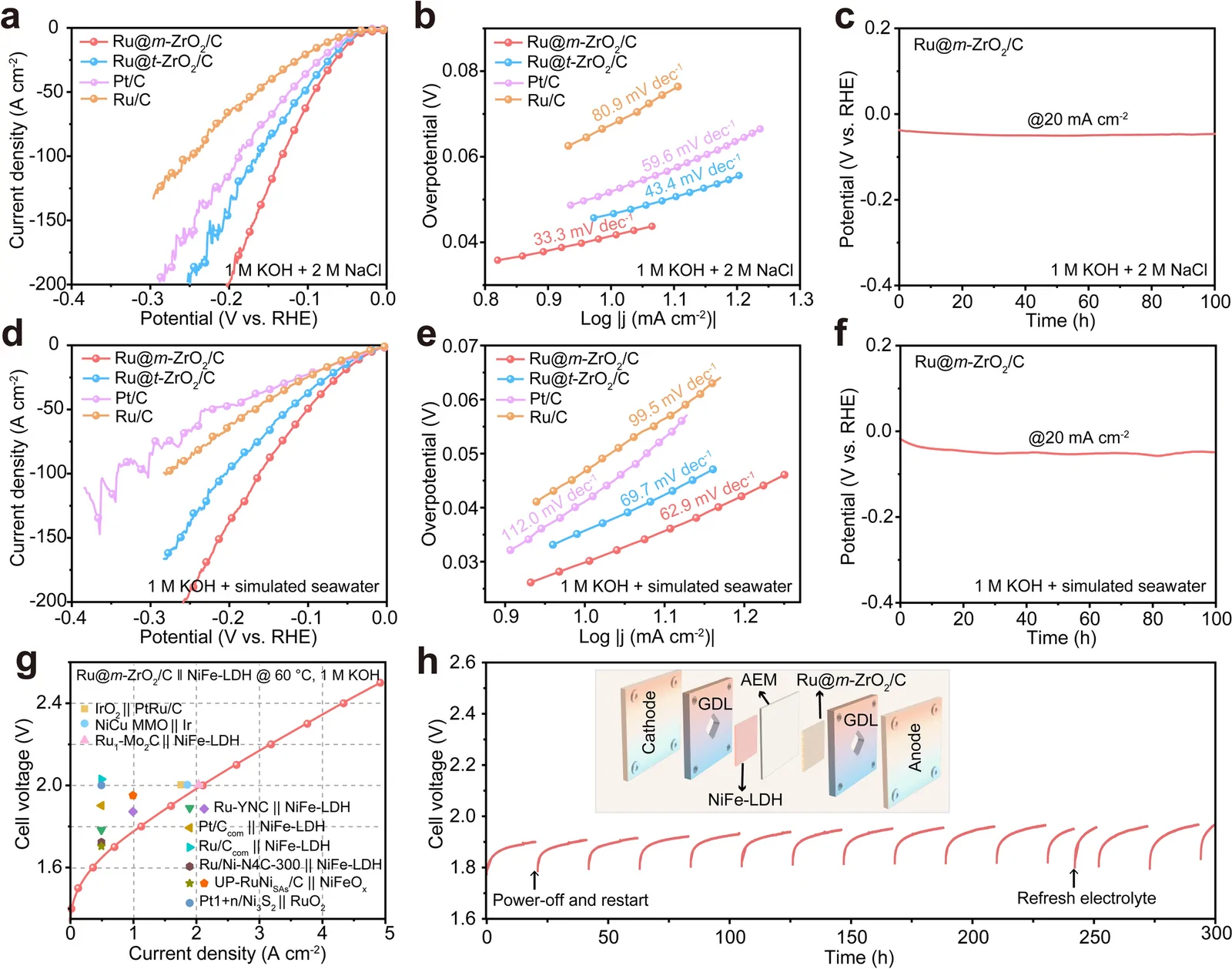
HER performance evaluation in practical application. **a** LSV polarization curves and **b** Tafel slopes measured in 1 M KOH + 2 M NaCl for Ru@*m*-ZrO_2_/C, Ru@*t*-ZrO_2_/C, Pt/C, and Ru/C, **c** the chronopotentiometry curve of Ru@*m*-ZrO_2_/C in 1 M KOH + 2 M NaCl at 20 mA cm^−2^. **d** LSV polarization curves and **e** Tafel slopes measured in 1 M KOH + simulated seawater for Ru@*m*-ZrO_2_/C, Ru@*t*-ZrO_2_/C, Pt/C and Ru/C, **f** the chronopotentiometry curve of Ru@*m*-ZrO_2_/C in 1 M KOH + simulated seawater at 20 mA cm^−2^. **g** Polarization curve of Ru@*m*-ZrO_2_/C ‖ NiFe-LDH based AEMWE device measured at 60 °C in 1 M KOH, inset showing a comparison with reported literatures. **h** Chronopotentiometry curve of the Ru@*m*-ZrO_2_/C ‖ NiFe-LDH device under a current density of 1 A cm^−2^, inset showing a schematic illustration of the AEMWE device

Due to the growing scarcity of freshwater resources, direct seawater electrolysis for hydrogen production is increasingly imperative [79, 80]. In this context, we evaluated the HER performance of the Ru@*m*-ZrO_2_/C catalyst in 1 M KOH with *x* M NaCl (*x* = 0. 0.5, 2) solution and alkaline simulated seawater (1 M KOH + simulated seawater) environments. As shown in Figs. 6a and S57, the catalyst exhibits slightly decreased HER activity in 1 M KOH + 2 M NaCl compared to pure 1 M KOH, yet still achieves a low overpotential of 42 mV at 10 mA cm^−2^ and a Tafel slope of 33.3 mV dec^−1^ (Fig. 6b), significantly outperforming Ru@*t*-ZrO_2_/C, Pt/C, and Ru/C. To systematically assess chloride corrosion resistance, we tested the catalyst under varying Cl^−^ concentrations (*x* = 0, 0.5, 2 M NaCl in 1 M KOH), which reveals dramatically robust Cl^−^ resistance capability (Fig. S58) [81, 82]. Furthermore, in situ XPS result indicated that the electronic structures of Ru and Zr retained intact after Cl^−^ adsorption (Figs. S59 and S60). Besides, DFT calculated adsorption energies of Cl^−^ range from − 1.47 to − 1.30 eV (Fig. S61), indicating spontaneous Cl^−^ adsorption onto Ru active sites under experimental conditions. After Cl^−^ adsorption, the ΔG_H*_ at the top Ru site lies between − 0.01 and 0.06 eV, which is comparable to or even slightly better than that without Cl^−^ (− 0.06 eV). Thus, Cl^−^ adsorption does not adversely affect the intrinsic HER activity of the Ru sites. Chronopotentiometry tests at 20 mA cm^−2^ for ~ 100 h further confirm the exceptional stability of Ru@*m*-ZrO_2_/C in chloride-containing environments, demonstrating strong corrosion resistance (Figs. 6c and S63–S65, Table S14).

The HER performance of Ru@*m*-ZrO_2_/C was further examined in a more realistic alkaline seawater system (1 M KOH + simulated seawater). Although the addition of KOH leads to precipitation of Ca^2+^ and Mg^2+^, which can block active sites [83], the catalyst requires an overpotential of only 30 mV to reach 10 mA cm^−2^ (Figs. 6d and S62). This value is lower than those of Ru@*t*-ZrO_2_/C (36 mV), Pt/C (42 mV), and Ru/C (47 mV) and ranks among the best reported catalysts (Table S14). Moreover, Ru@*m*-ZrO_2_/C shows a Tafel slope of 62.9 mV dec^−1^, superior to Ru@*t*-ZrO_2_/C (69.7 mV dec^−1^), Pt/C (112.0 mV dec^−1^), and Ru/C (99.5 mV dec^−1^), indicating favorable reaction kinetics in seawater electrolyte (Fig. 6e). The excellent stability of the catalyst in simulated seawater was further verified by chronopotentiometry at 20 mA cm^−2^ for 100 h (Fig. 6f and Table S14). Post-stability characterization by XRD, Raman and SEM verified the structural integrity of Ru@*m*-ZrO_2_/C (Figs. S63–S65). Furthermore, to assess potential surface blocking by Ca^2+^ and Mg^2+^ ions, XPS and TEM analyses were conducted on the tested samples. The results show that the Ru@*m*-ZrO_2_/C maintained its structural stability after the test, with only minimal amounts of Ca^2+^ and Mg^2+^ ions detected (Figs. S66 and S67). Given its outstanding HER performance in both alkaline and seawater media, we assembled an AEMWE device using Ru@*m*-ZrO_2_/C as the cathode and NiFe-LDH as the anode. Operating at 60 °C in 1 M KOH, the electrolyzer achieved current densities of 0.50, 1.0, and 2.0 A cm^−2^ at cell voltages of 1.64, 1.76, and 1.98 V, respectively (Fig. 6g). These results surpass most reported electrocatalysts (Fig. 6g and Table S15). In view of the intermittent supply of renewable energy (solar, wind, *etc.*), the catalyst stability was studied by simulating the frequent start–stop operation cycles of water electrolysis. The system demonstrated remarkable stability, maintaining operation at an industrial-level current density of 1 A cm^−2^ for more than 300 h (Fig. 6h), highlighting its potential for practical high-current–density water electrolysis and commercial application.

## Conclusions

In summary, this work establishes that the crystal phase of ZrO_2_ significantly influences the electronic metal–support interaction and hydrogen reverse spillover process, thereby dictating the HER performance of Ru nanoclusters. In situ Raman and EIS analysis revealed the enhanced interfacial water activation and dissociation process on *m*-ZrO_2_. DFT calculations further unveiled that work function-induced charge accumulation at the Ru/*m*-ZrO_2_ interface facilitates water adsorption and dissociation at Zr sites and accelerates reverse hydrogen spillover from the *m*-ZrO_2_ substrate to Ru sites by regulating the *d*-band center of Ru, thereby enhancing electrocatalytic HER performance. These fundamental insights, coupled with the exceptional catalytic performance—exemplified by an overpotential of 28 mV in 1 M KOH and 30 mV in alkaline seawater at 10 mA cm^−2^, and stable AEMWE performance at industrial current density for more than 300 h—demonstrate the great potential of phase‐engineered supports in designing highly efficient and durable electrocatalysts for renewable hydrogen production.

## Supplementary Information

Below is the link to the electronic supplementary material.

Supplementary file1 (DOCX 21334 kb)
