# Supplementary material for: Crystal Phase Engineering Accelerates Hydrogen Reverse Spillover for Efficient Alkaline Hydrogen Production
Source: Nanomicro Lett. 2026 Jul 13;18:438. doi: 10.1007/s40820-026-02289-3 (PMC13365097; doi:10.1007/s40820-026-02289-3)
Supplement: Supplementary file 1 — Supplementary file1 (DOCX 21334 kb) [file 40820_2026_2289_MOESM1_ESM.docx]

Supporting Information for

**Crystal Phase Engineering Accelerates Hydrogen Reverse Spillover for Efficient Alkaline Hydrogen Production**

Jun Zhang^†1^, Xiaoyu Chen^†2^, Bin Wu^†3,^*, Xiangyang Guo^4^, Xianlin Qu^5^, Qunzhi Ma^1^, Ying Wang^1^, Jiayi Li^1^, Wei Liu^1^, Xu Li^1^, Liyun Cao^1^, Yi Wang^5^, Jianfeng Huang^1,^*, Jingxiang Zhao^2,^*, Fuxiang Zhang^4,^*, and Yongqiang Feng^1,^*

^1^ School of Materials Science and Engineering, Shaanxi Key Laboratory of Green Preparation and Functionalization for Inorganic Materials, Shaanxi University of Science and Technology, Xi’an 710021, P. R. China

^2^ College of Chemistry and Chemical Engineering, Harbin Normal University, Harbin 150025, P. R. China

^3^ School of Materials Science and Engineering, Nanyang Technological University, Singapore 639798, Singapore

^4^ State Key Laboratory of Catalysis, Dalian Institute of Chemical Physics, Chinese Academy of Sciences, Dalian National Laboratory for Clean Energy, Dalian 116023, P. R. China

^5^ Center for Microscopy and Analysis, Nanjing University of Aeronautics and Astronautics, Nanjing 211106, P. R. China

† Jun Zhang, Xiaoyu Chen, and Bin Wu contribute equally to this work.

*Corresponding authors. E-mail: [bin.wu@ntu.edu.sg](mailto:bin.wu@ntu.edu.sg) (Bin Wu); [huangjf@sust.edu.cn](mailto:huangjf@sust.edu.cn) (Jianfeng Huang); zhaojingxiang@hrbnu.edu.cn (Jingxiang Zhao); [fxzhang@dicp.ac.cn](mailto:fxzhang@dicp.ac.cn) (Fuxiang Zhang); fengyq@sust.edu.cn (Yongqiang Feng)

Supplementary Figures and Tables


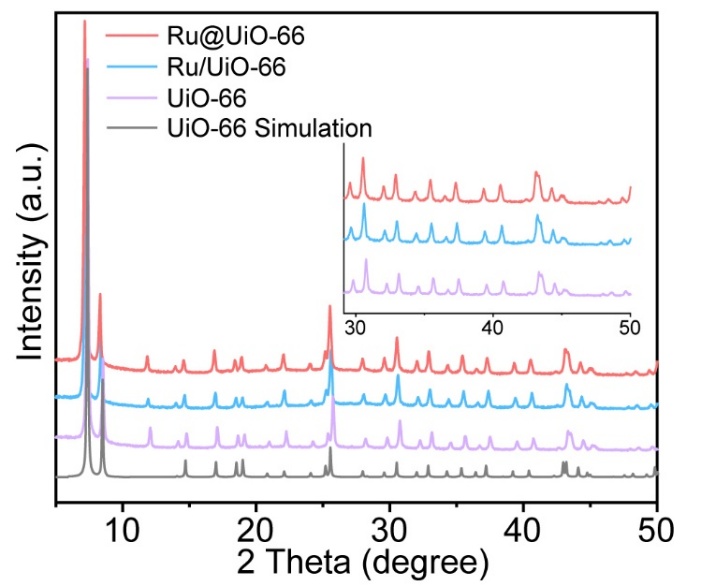


**Fig. S1** XRD patterns of Ru@UiO-66, Ru/UiO-66 and UiO-66.


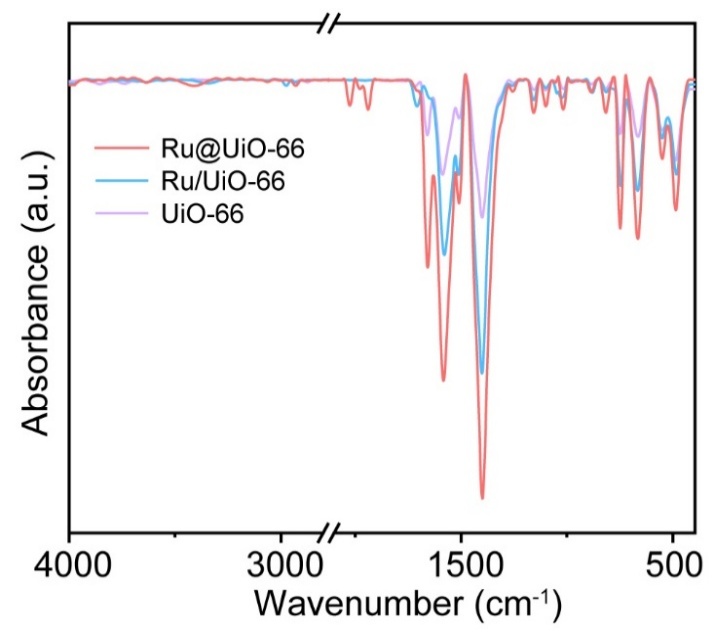


**Fig. S2** FT-IR spectra of Ru@UiO-66, Ru/UiO-66 and UiO-66.


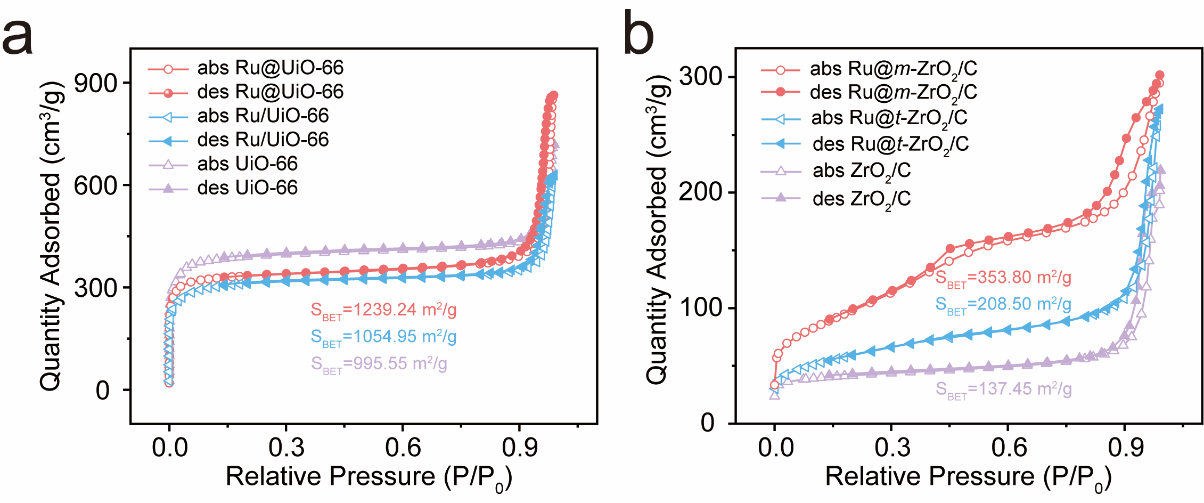


**Fig. S3** N_2_ isothermal adsorption/desorption curves of **a** Ru@UiO-66, Ru/UiO-66 and UiO-66, and **b** Ru@*m*-ZrO_2_/C, Ru@*t*-ZrO_2_/C and ZrO_2_/C.


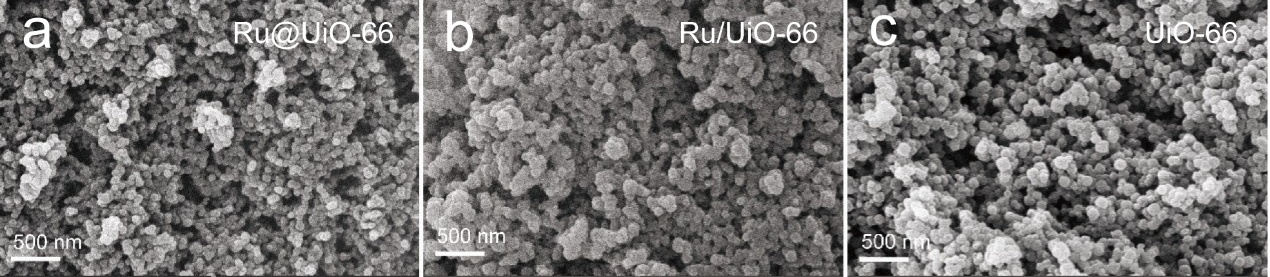


**Fig. S4** SEM images of **a** Ru@UiO-66, **b** Ru/UiO-66 and **c** UiO-66.


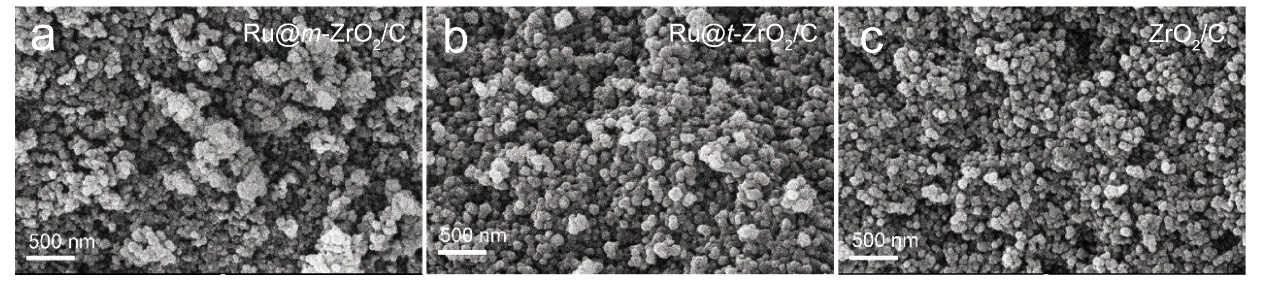


**Fig. S5** SEM images of **a** Ru@*m*-ZrO_2_/C, **b** Ru@*t*-ZrO_2_/C and **c** ZrO_2_/C.


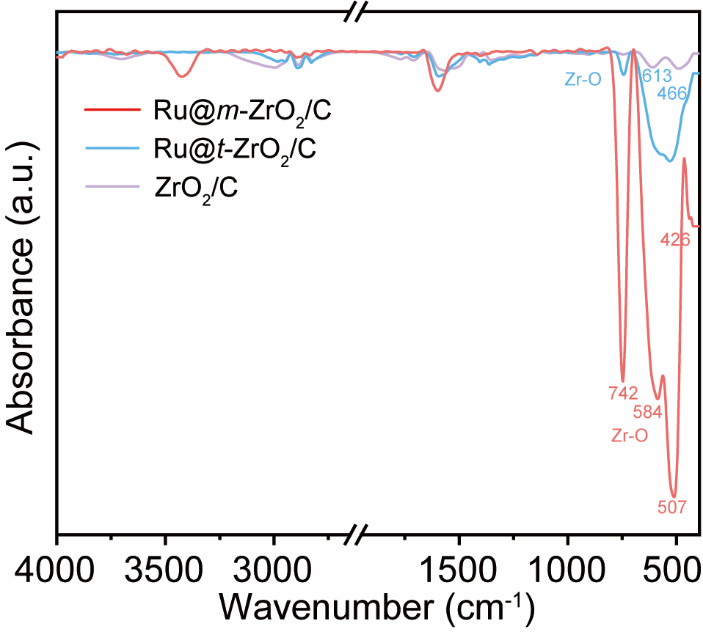


**Fig. S6** FT-IR spectra of Ru@*m*-ZrO_2_/C, Ru@*t*-ZrO_2_/C and ZrO_2_/C.


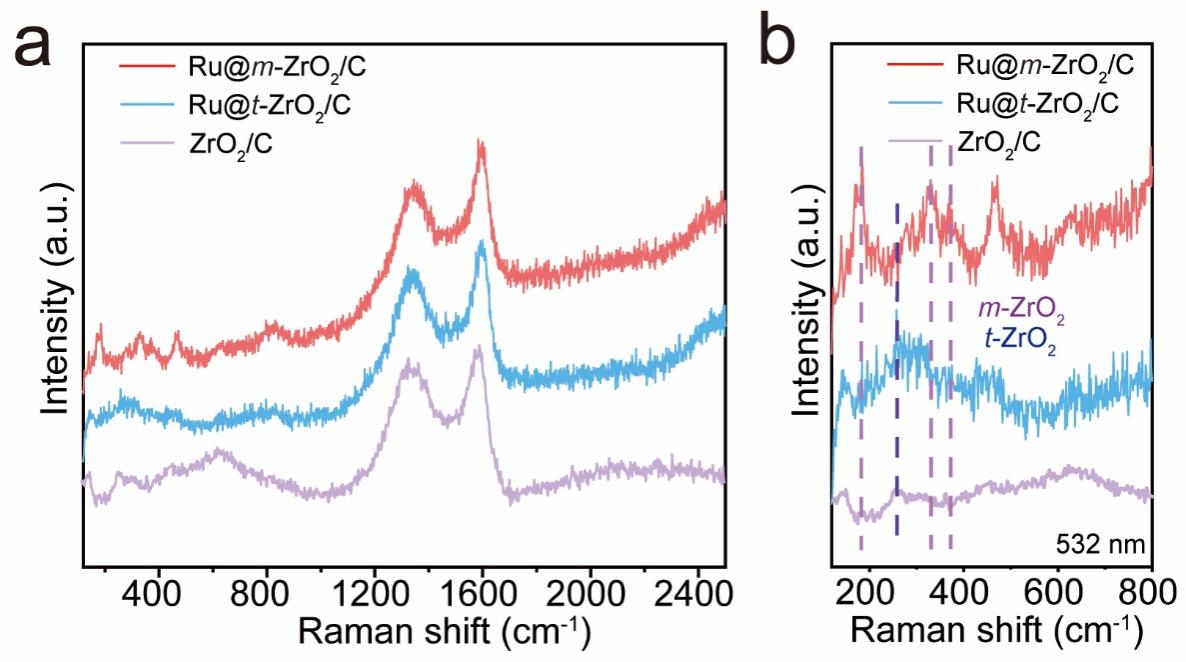


**Fig. S7** Raman spectra of Ru@*m*-ZrO_2_/C, Ru@*t*-ZrO_2_/C and ZrO_2_/C in the range of **a** 150~2500 cm^-1^ and **b** 150~800 cm^-1^.


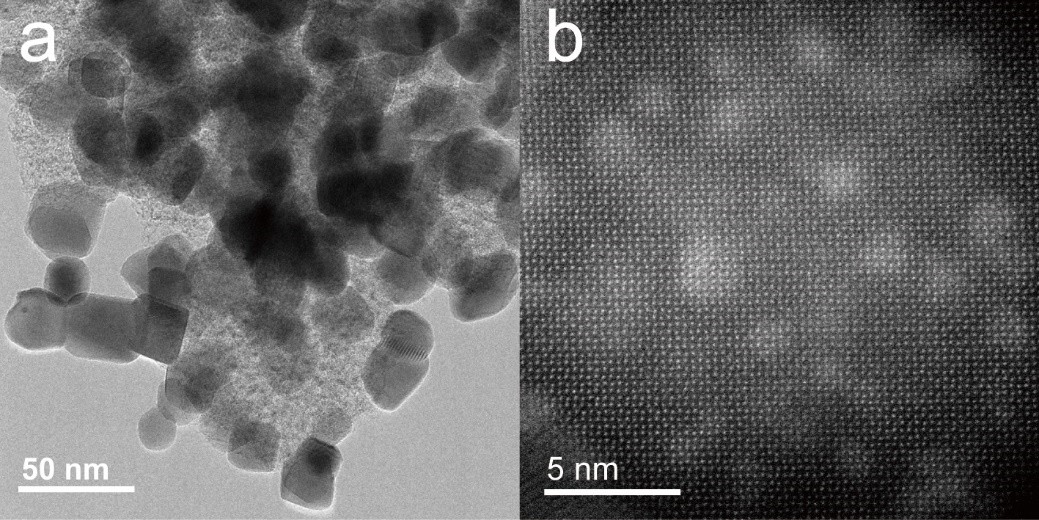


**Fig. S8** **a** HRTEM and **b** HAADF-STEM image of Ru@*m*-ZrO_2_/C.


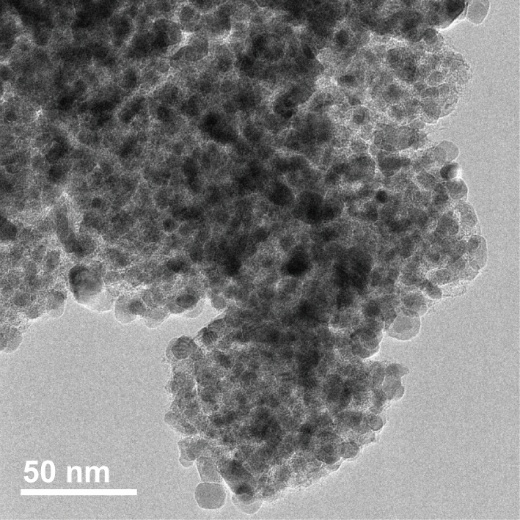


**Fig. S9** HRTEM image of Ru@*t*-ZrO_2_/C.


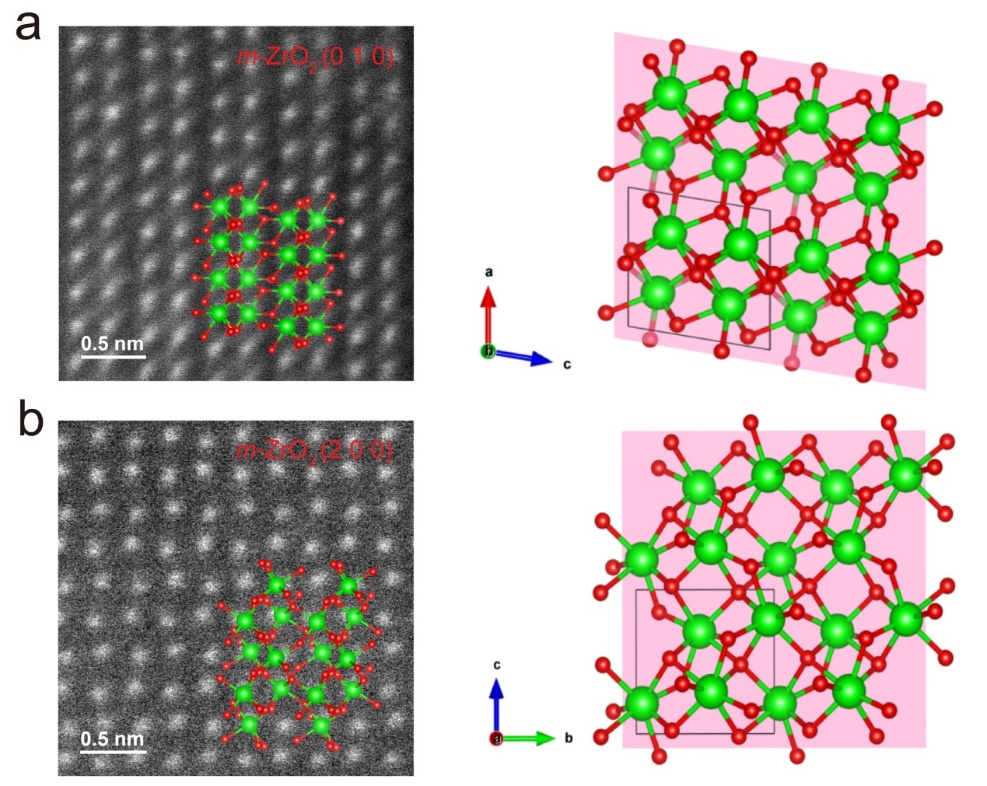


**Fig. S10** HAADF-STEM image of Ru@*m*-ZrO_2_/C and the corresponding structural model of *m*-ZrO_2_ along **a** [010] and **b** [200] zone axis.


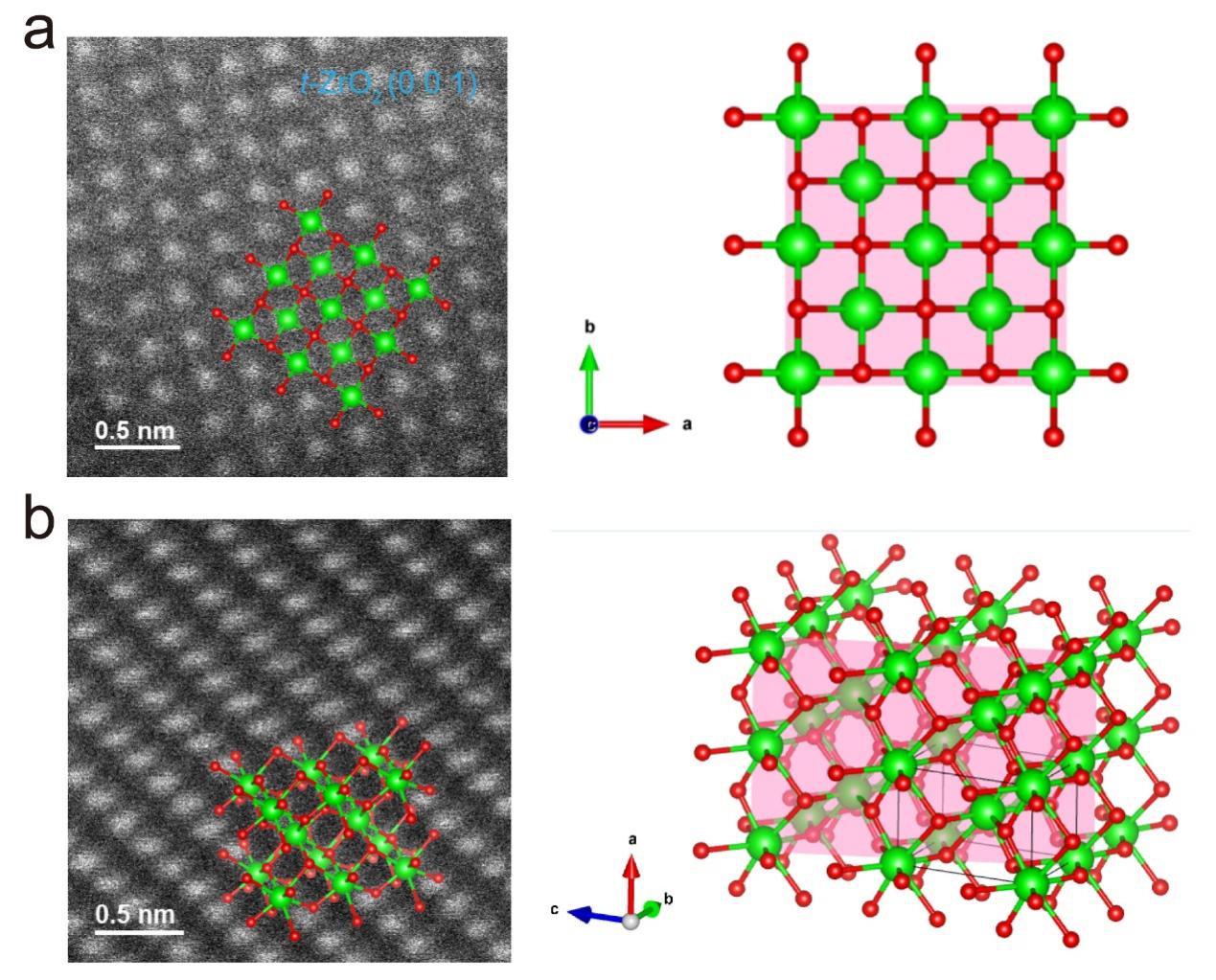


**Fig. S11** HAADF-STEM image of Ru@*t*-ZrO_2_/C and the corresponding structural model of *t*-ZrO_2_.


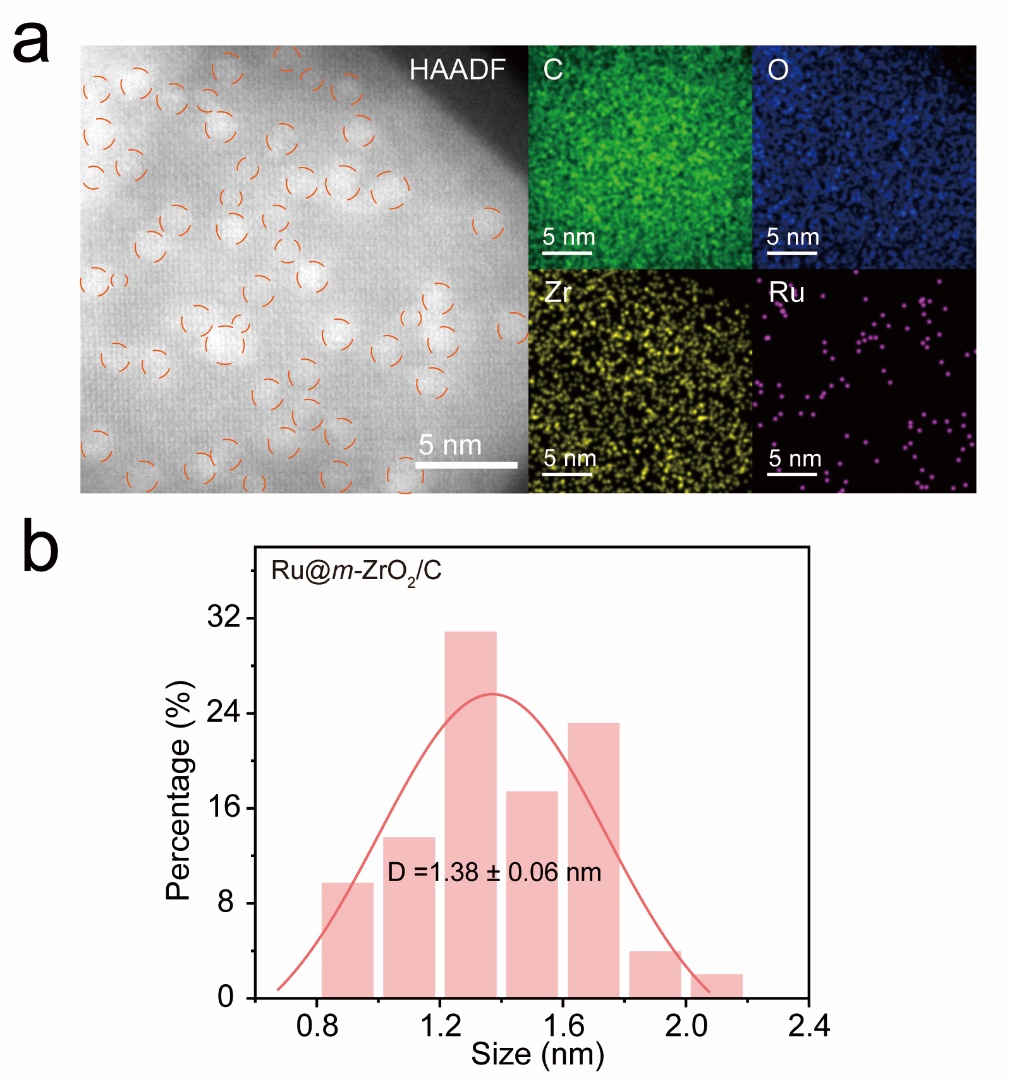


**Fig. S12** **a** HAADF image of Ru@*m*-ZrO_2_/C and the corresponding elemental mapping for C (green), O (blue), Zr (yellow) and Ru (purple). **b** size distribution histogram of Ru clusters in Ru@*m*-ZrO_2_/C.


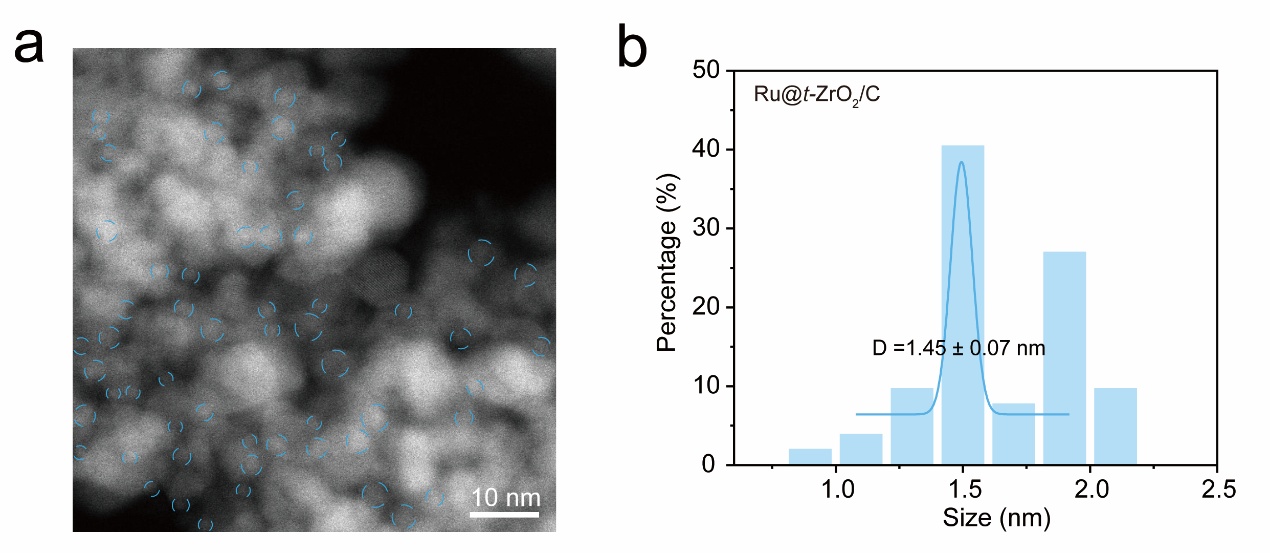


**Fig. S13** **a** HRTEM image of Ru@*t*-ZrO_2_/C and **b** size distribution histogram of Ru clusters in Ru@*t*-ZrO_2_/C.


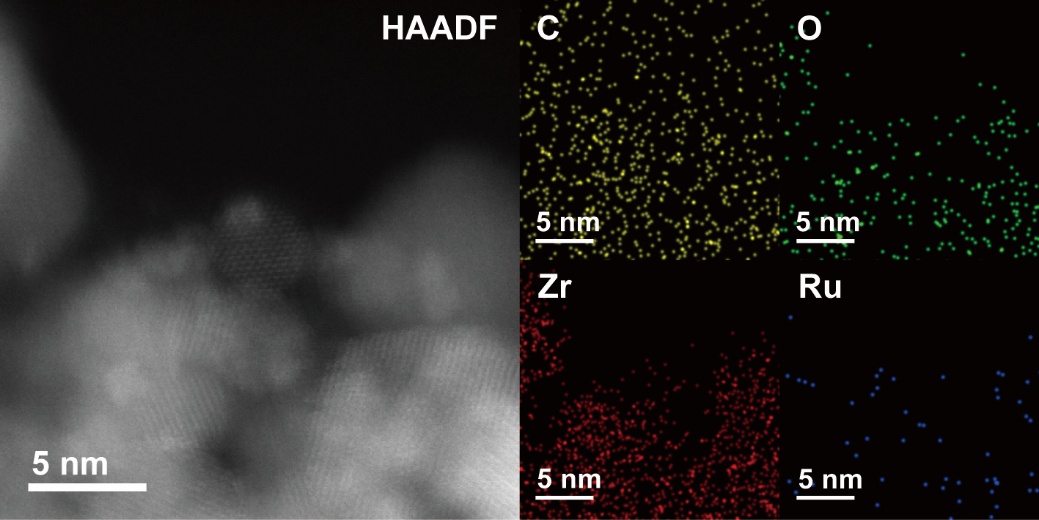


**Fig. S14** HAADF image and the corresponding elemental mapping for C (yellow), O (green), Zr (red) and Ru (blue) in Ru@*t*-ZrO_2_/C.


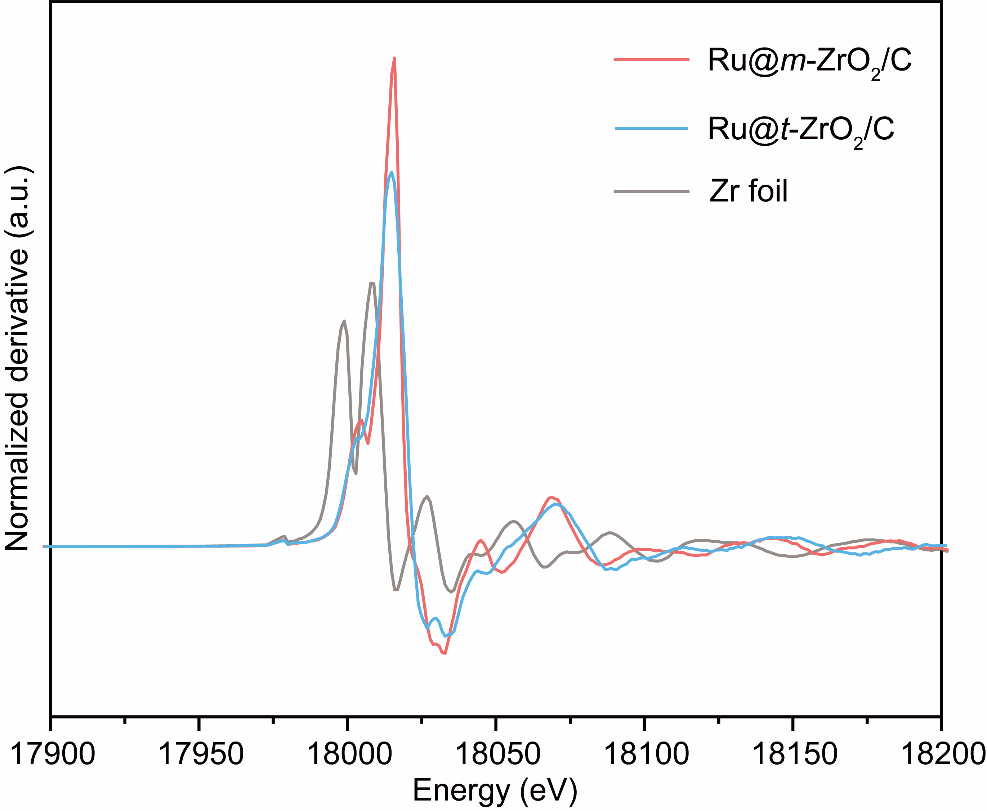


**Fig. S15** The first derivative of Zr K-edge XANES for Ru@*m*-ZrO_2_/C, Ru@*t*-ZrO_2_/C and Zr foil.


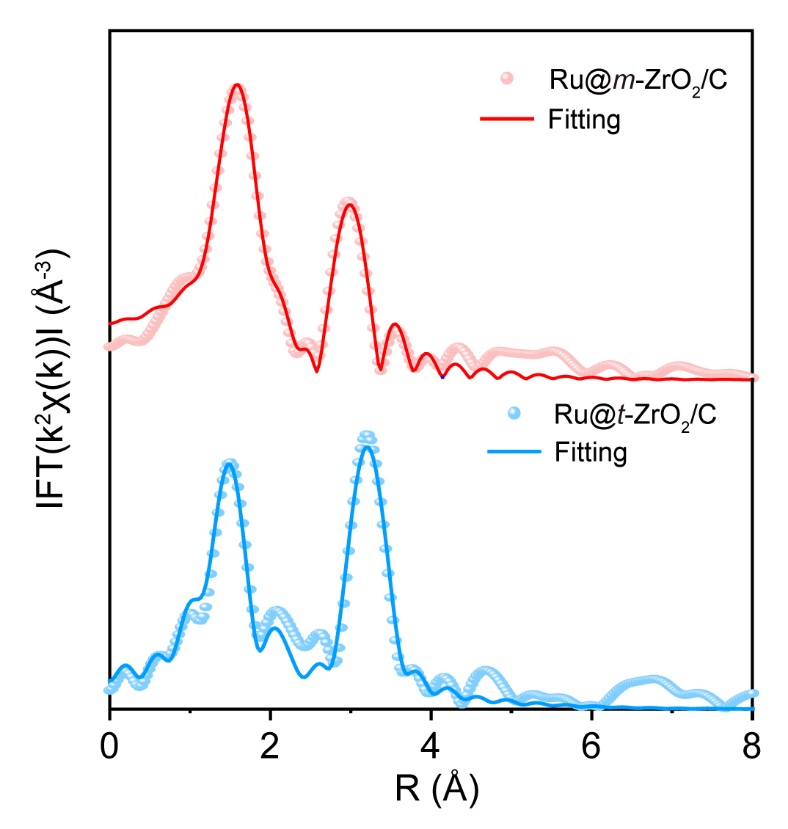


**Fig. S16** Fitting curves of Zr K-edge EXAFS for Ru@*m*-ZrO_2_/C and Ru@*t*-ZrO_2_/C at R-space.


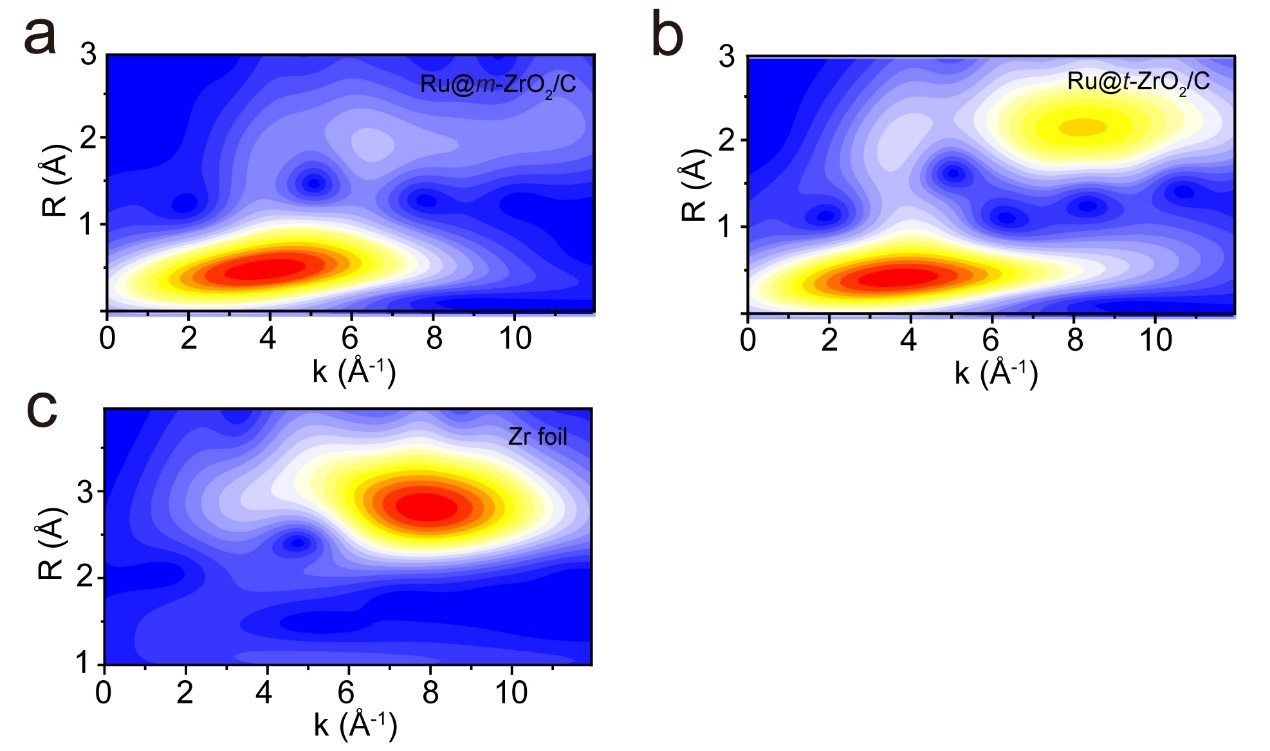


**Fig. S17** Wavelet transforms of the EXAFS spectra at Zr K-edge for **a** Ru@*m*-ZrO_2_/C, **b** Ru@*t*-ZrO_2_/C and **c** Zr foil.


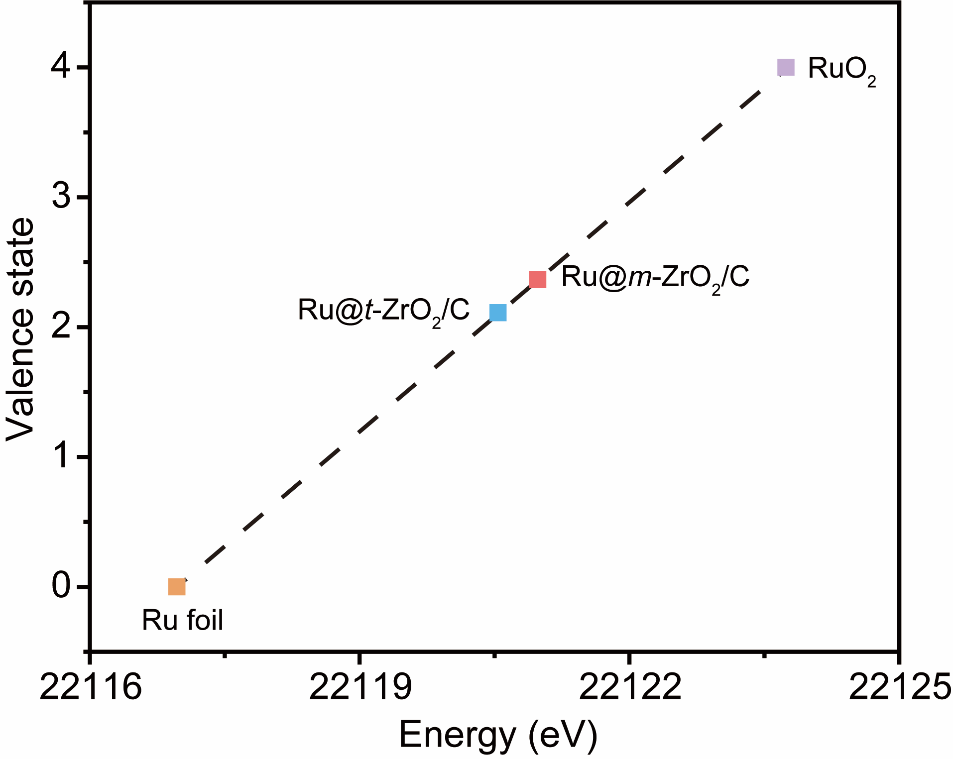


**Fig. S18** The average oxidation state of Ru in Ru@*m*-ZrO_2_/C and Ru@*t*-ZrO_2_/C derived from the Ru K-edge XANES.


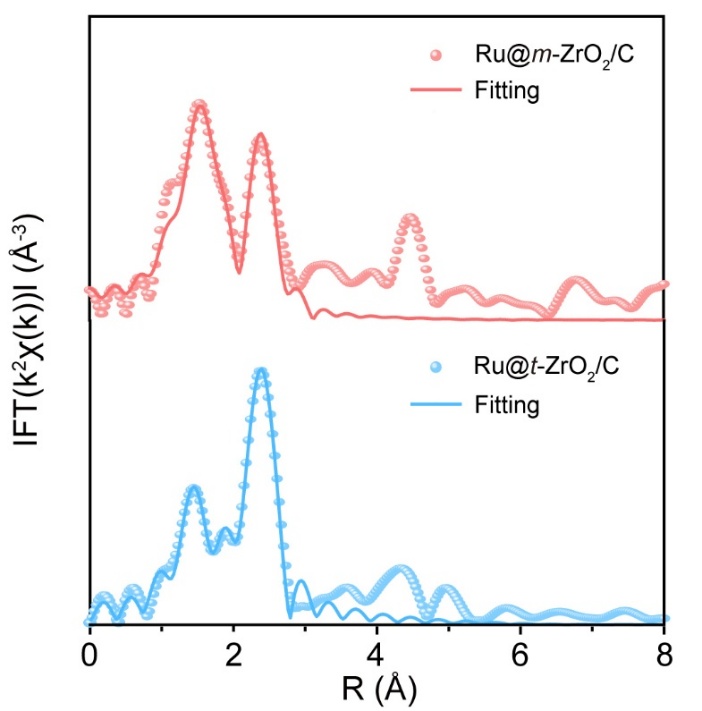


**Fig. S19** Fitting curves of Ru K-edge EXAFS for Ru@*m*-ZrO_2_/C and Ru@*t*-ZrO_2_/C at R-space.


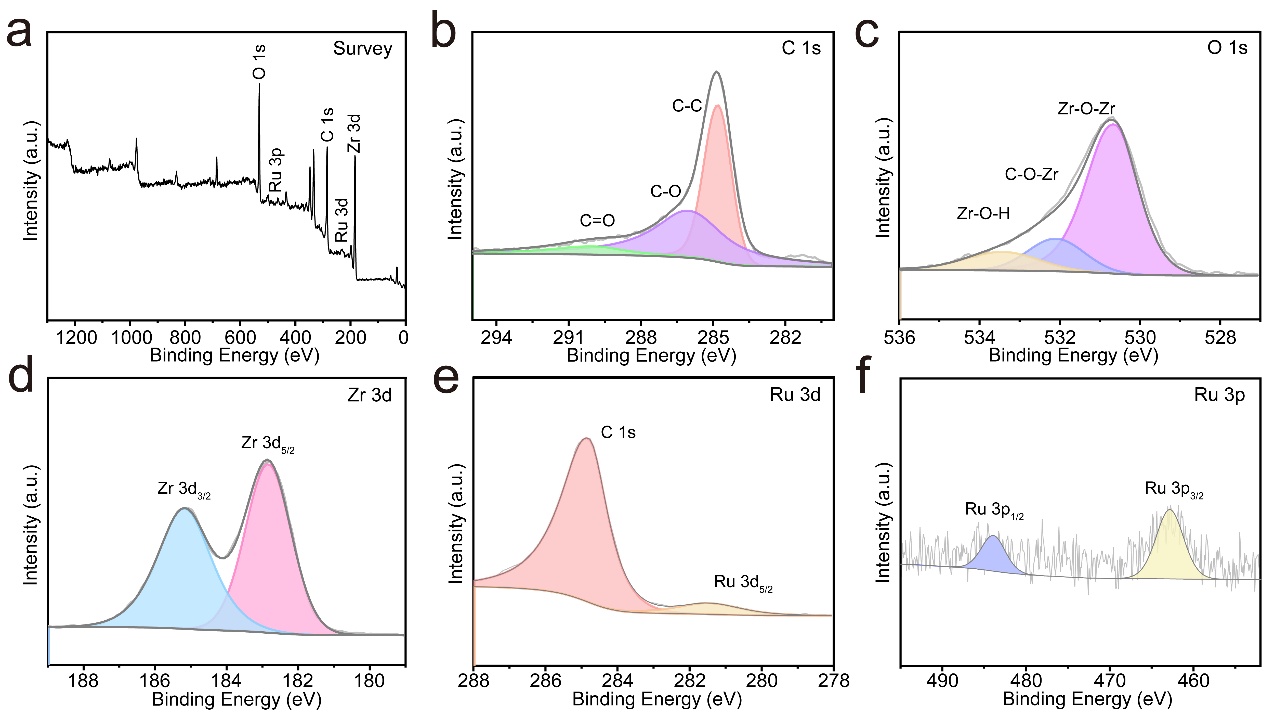


**Fig. S20** XPS spectra of Ru@*m*-ZrO_2_/C for **a** survey, **b** C 1s, **c** O 1s, **d** Zr 3d, **e** Ru 3d and **f** Ru 3p.


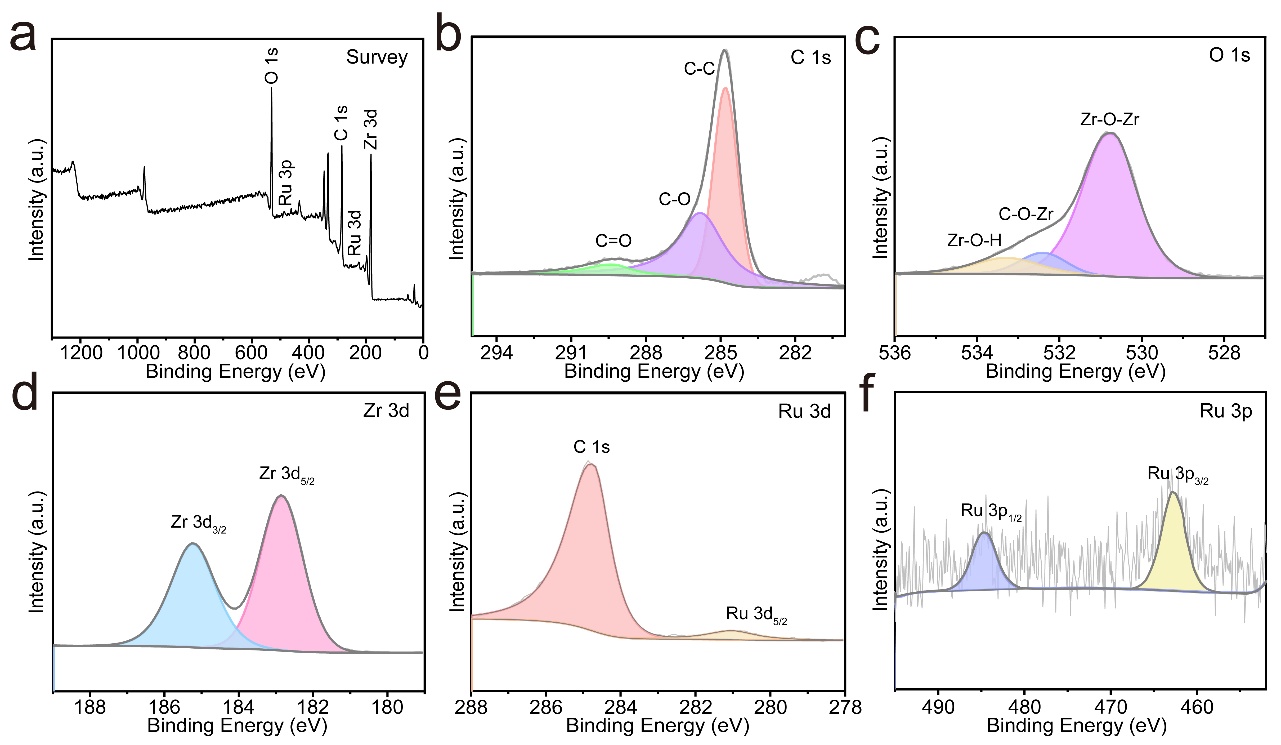


**Fig. S21** XPS spectra of Ru@*t*-ZrO_2_/C for **a** survey, **b** C 1s, **c** O 1s, **d** Zr 3d, **e** Ru 3d and **f** Ru 3p.


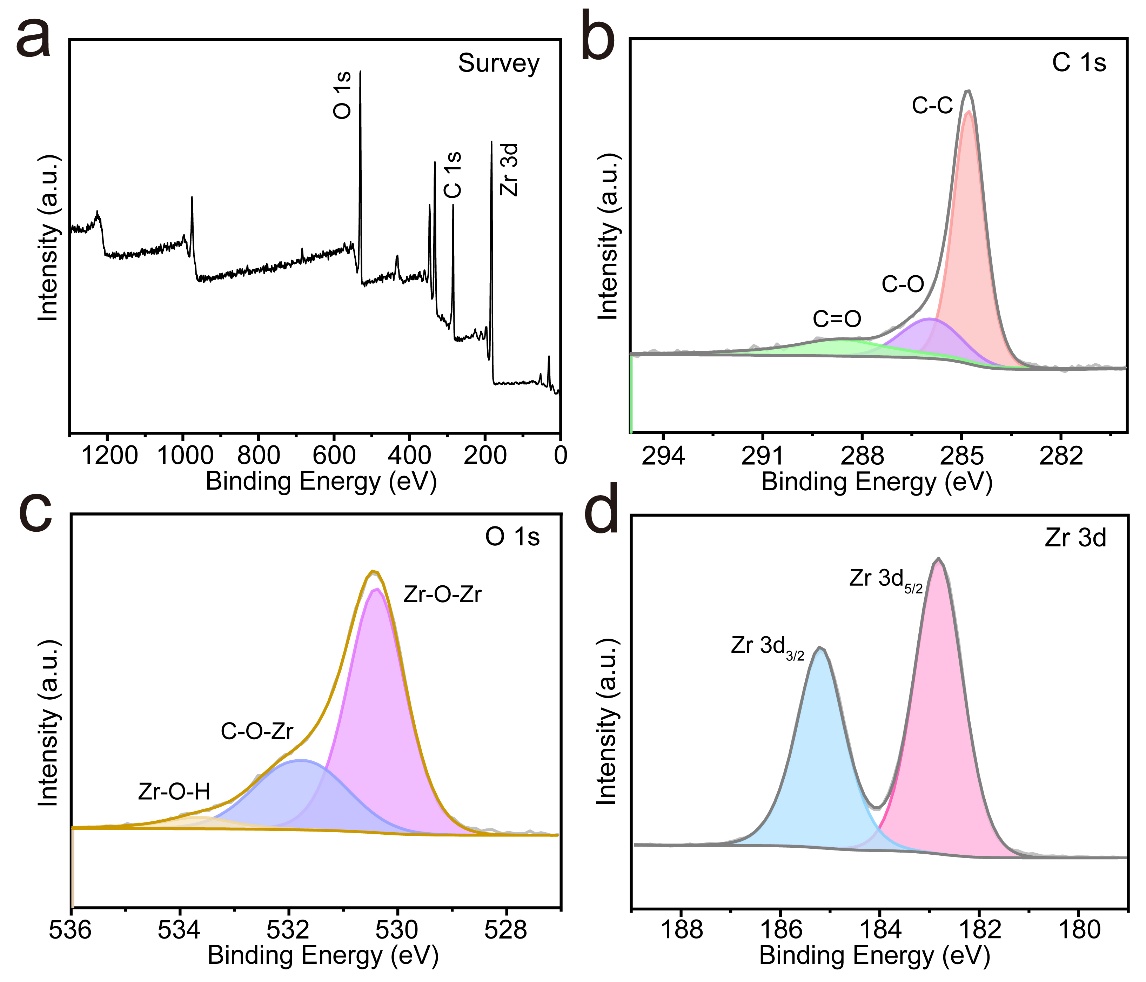


**Fig. S22** XPS spectra of ZrO_2_/C for **a** survey, **b** C 1s, **c** O 1s and **d** Zr 3d.


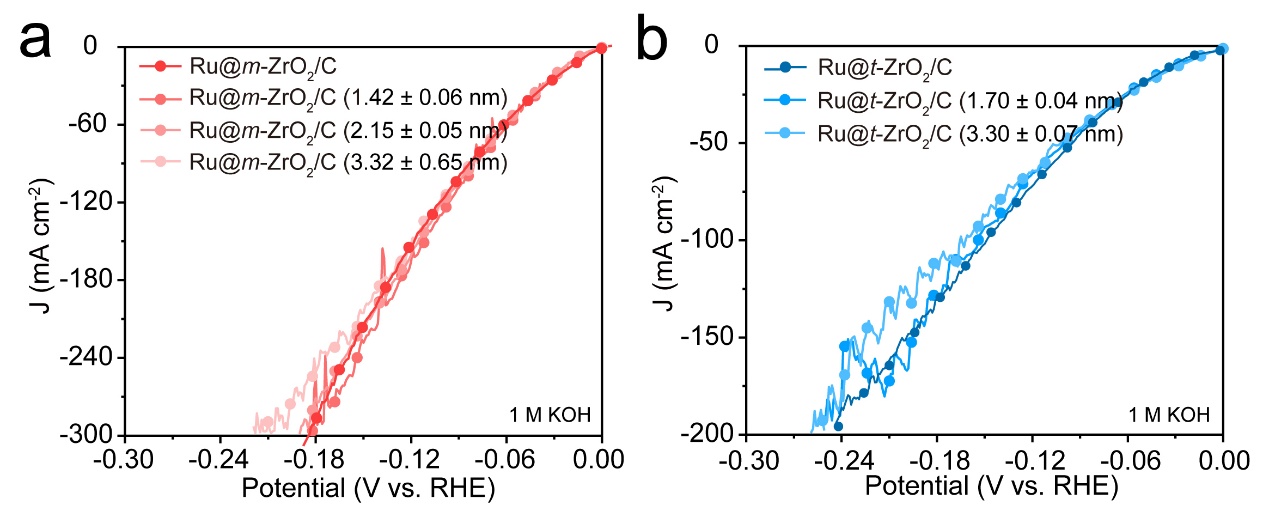


**Fig. S23** LSV curves of **a** Ru@*m*-ZrO_2_/C, Ru@*m*-ZrO_2_/C (1.42 ± 0.06 nm), Ru@*m*-ZrO_2_/C (2.15 ± 0.05 nm) and Ru@*m*-ZrO_2_/C (3.32 ± 0.65 nm), **b** Ru@*t*-ZrO_2_/C, Ru@*t*-ZrO_2_/C (1.70 ± 0.04 nm) and Ru@*t*-ZrO_2_/C (3.30 ± 0.07 nm) in 1 M KOH.


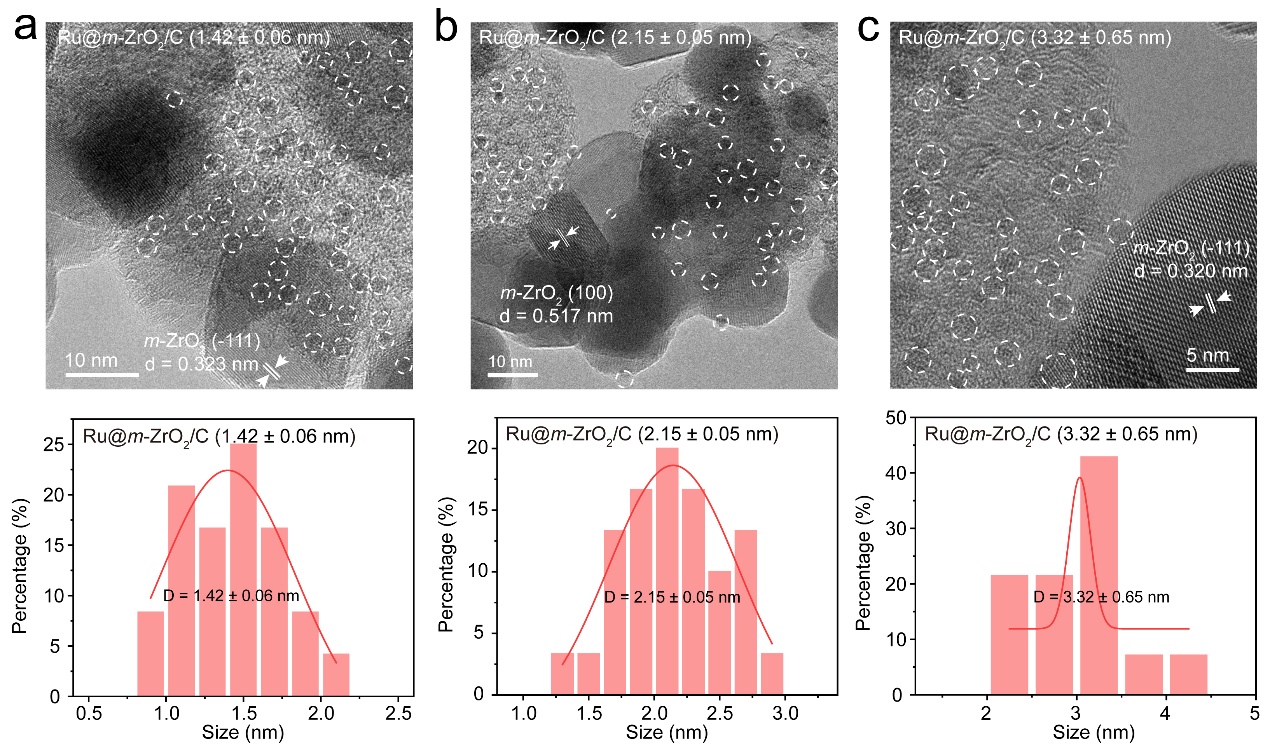


**Fig. S24** HRTEM images and size distribution histogram of Ru clusters in **a** Ru@*m*-ZrO_2_/C (1.42 ± 0.06 nm), **b** Ru@*m*-ZrO_2_/C (2.15 ± 0.05 nm) and **c** Ru@*m*-ZrO_2_/C (3.32 ± 0.65 nm).


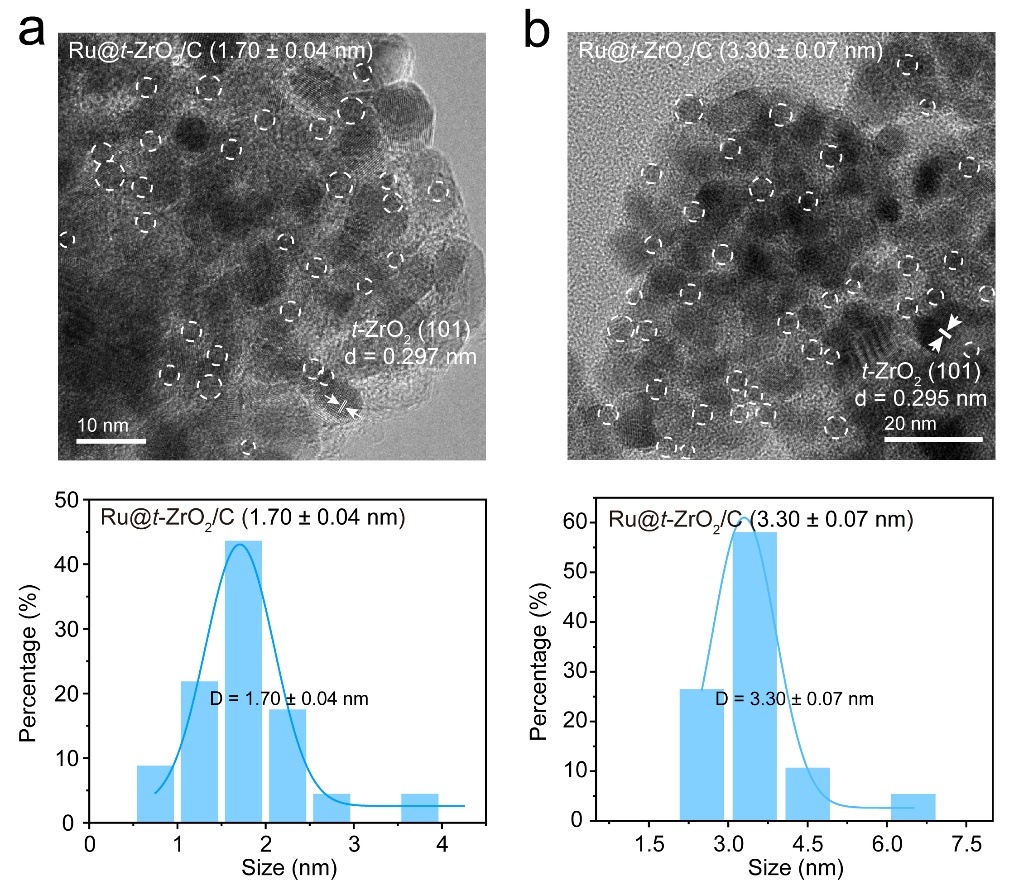


**Fig. S25** HRTEM images and size distribution histogram of Ru clusters in **a** Ru@*t*-ZrO_2_/C (1.70 ± 0.04 nm) and **b** Ru@*t*-ZrO_2_/C (3.30 ± 0.07 nm).


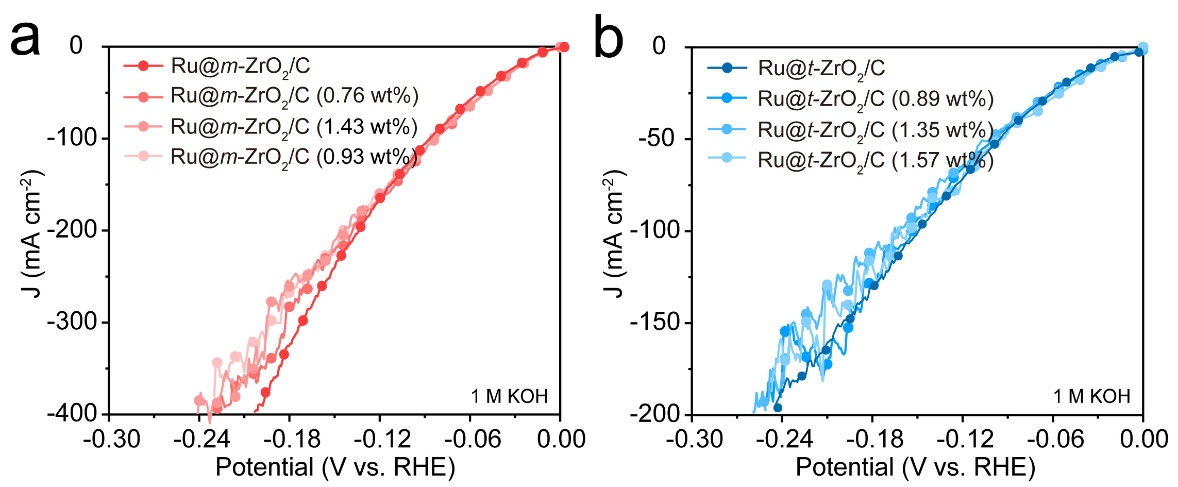


**Fig. S26** LSV curves of **a** Ru@*m*-ZrO_2_/C, Ru@*m*-ZrO_2_/C (0.76 wt%), Ru@*m*-ZrO_2_/C (1.43 wt%) and Ru@*m*-ZrO_2_/C (0.93 wt%), **b** Ru@*t*-ZrO_2_/C, Ru@*t*-ZrO_2_/C (0.89 wt%), Ru@*t*-ZrO_2_/C(1.35 wt%) and Ru@*t*-ZrO_2_/C(1.57 wt%) in 1 M KOH.


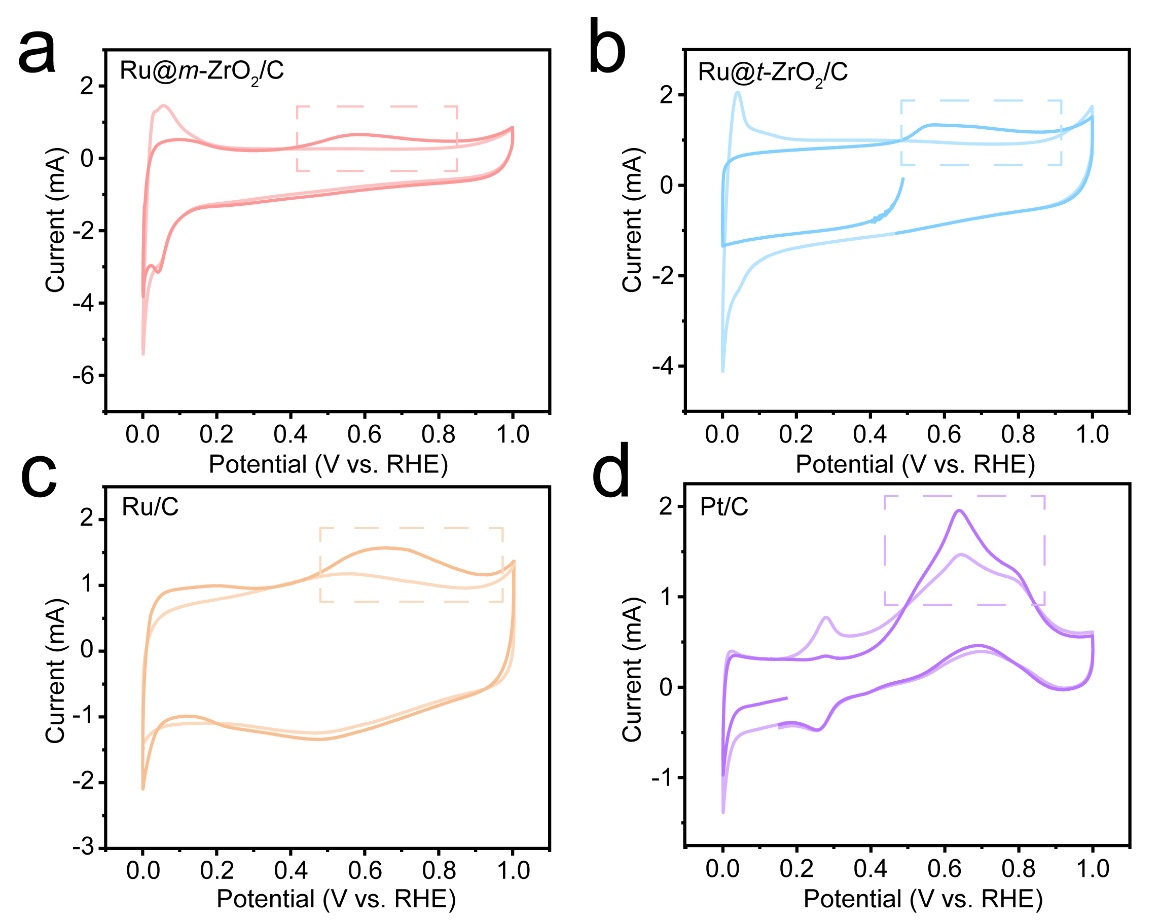


**Fig. S27** CO-stripping curves of **a** Ru@*m*-ZrO_2_/C, **b** Ru@*t*-ZrO_2_/C, **c** Ru/C and **d** Pt/C in 1 M KOH.


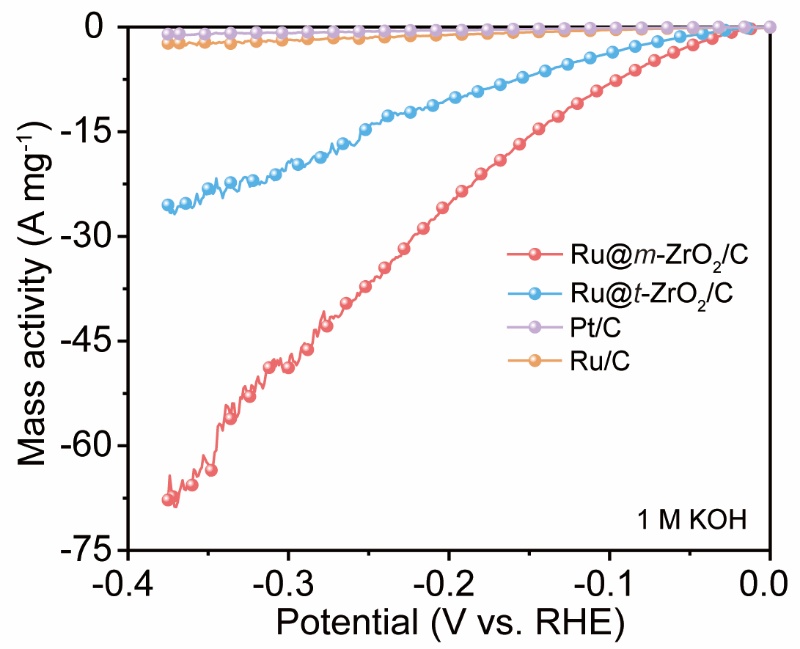


**Fig. S28** Mass-normalized LSV curves of Ru@*m*-ZrO_2_/C, Ru@*t*-ZrO_2_/C, Pt/C and Ru/C in 1 M KOH.


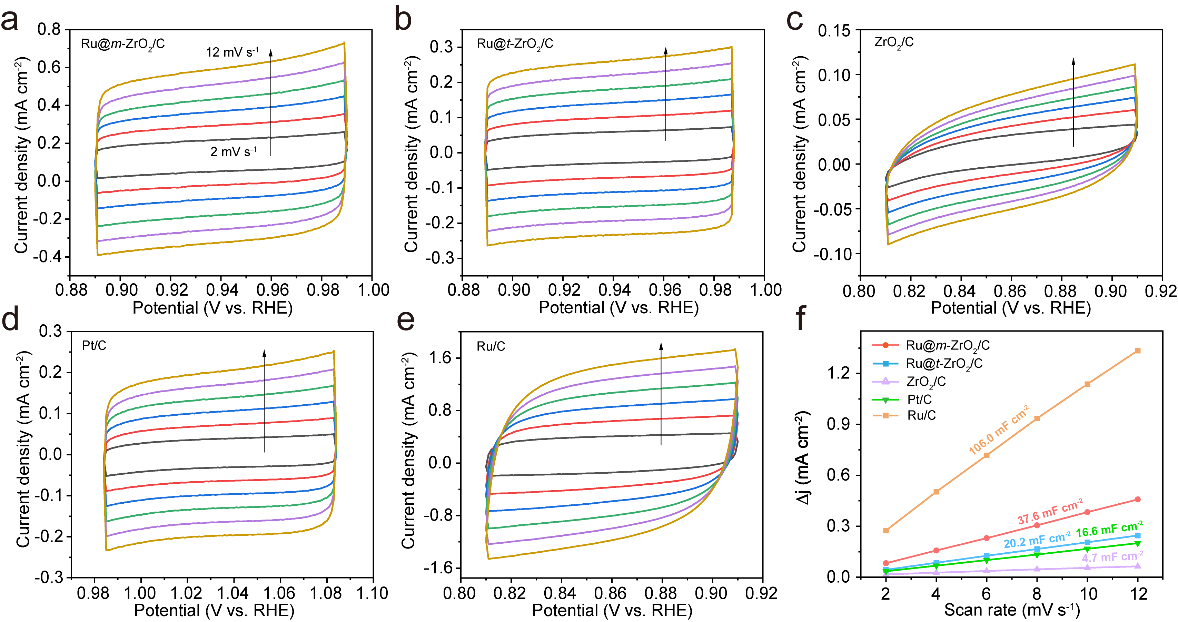


**Fig. S29.** Electrochemical cyclic voltammetry recorded in the non-Faradaic region in 1 M KOH for **a** Ru@*m*-ZrO_2_/C, **b** Ru@*t*-ZrO_2_/C, **c** ZrO_2_/C, **d** Pt/C and **e** Ru/C with scan rates of 2, 4, 6, 8, 10 and 12 mV s^-1^. **f** The corresponding calculated current density difference against scan rate.


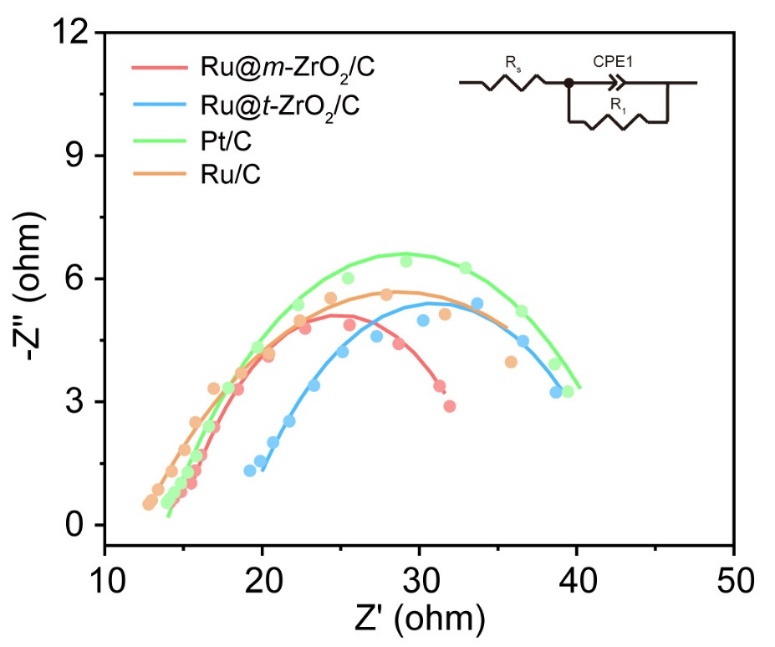


**Fig. S30** Nyquist plots of Ru@*m*-ZrO_2_/C, Ru@*t*-ZrO_2_/C, Pt/C and Ru/C during HER test in 1 M KOH.


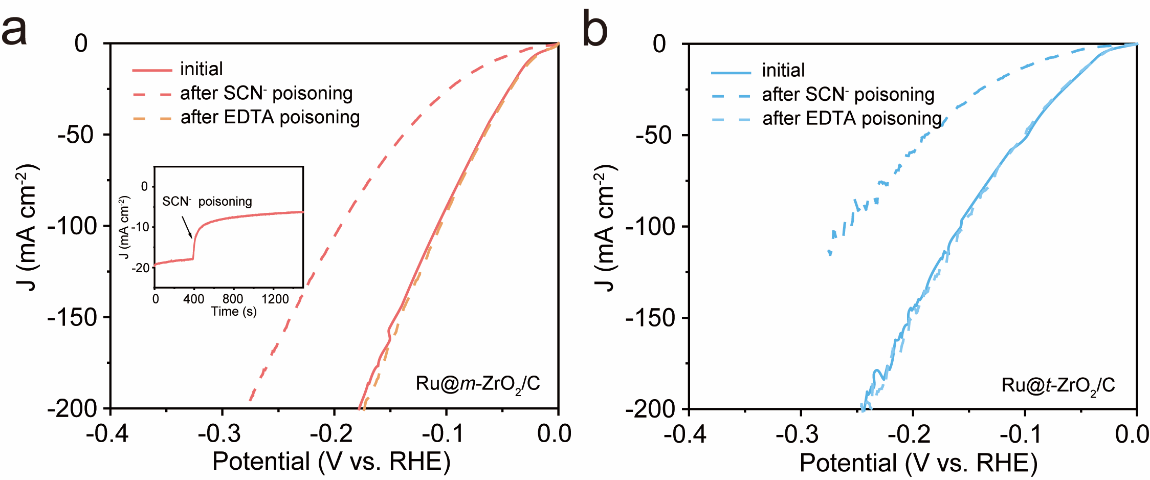


**Fig. S31** LSV curves of **a** Ru@*m*-ZrO_2_/C and **b** Ru@*t*-ZrO_2_/C in 1 M KOH before and after addition of 10 mM of KSCN and 10 mM of EDTA, respectively.


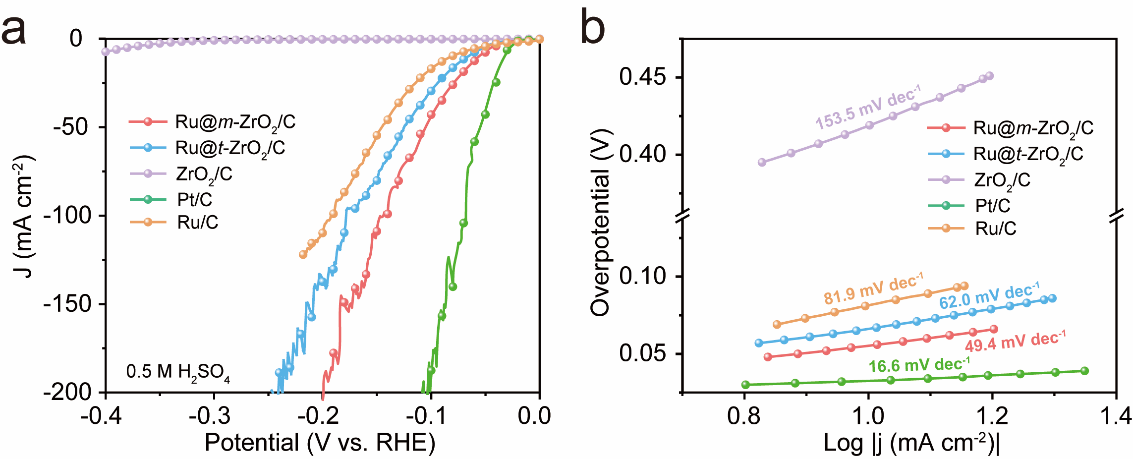


**Fig. S32** **a** LSV curves and **b** Tafel slopes of Ru@*m*-ZrO_2_/C, Ru@*t*-ZrO_2_/C, ZrO_2_/C, Pt/C and Ru/C in 0.5 M H_2_SO_4_.


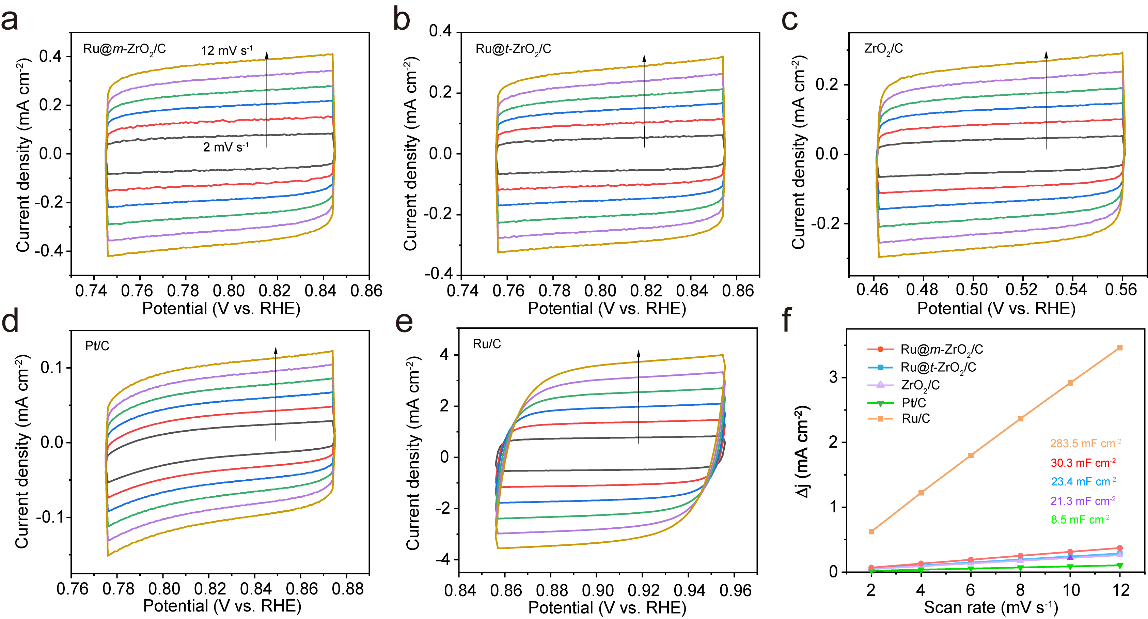


**Fig. S33** Electrochemical cyclic voltammetry recorded in the non-Faradaic region in 0.5 M H_2_SO_4_ for **a** Ru@*m*-ZrO_2_/C, **b** Ru@*t*-ZrO_2_/C, **c** ZrO_2_/C, **d** Pt/C and **e** Ru/C with scan rates of 2, 4, 6, 8, 10 and 12 mV s^-1^. **f** The corresponding calculated current density difference against scan rate.


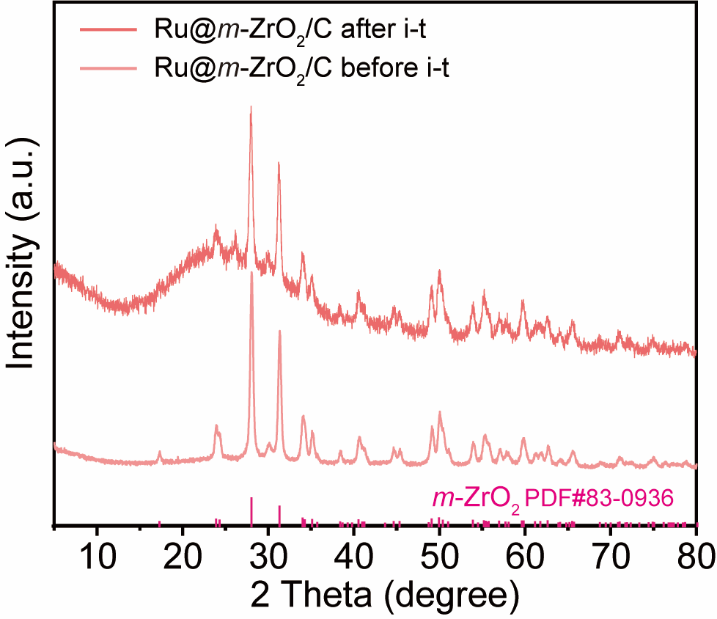


**Fig. S34** XRD pattern of Ru@*m*-ZrO_2_/C before and after i-t test in 1 M KOH.


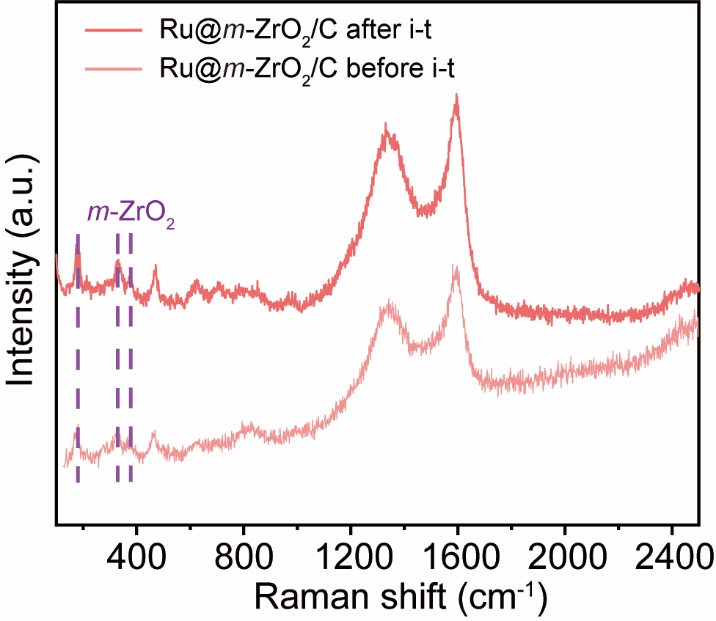


**Fig. S35** Raman spectra of Ru@*m*-ZrO_2_/C before and after i-t test in 1 M KOH.


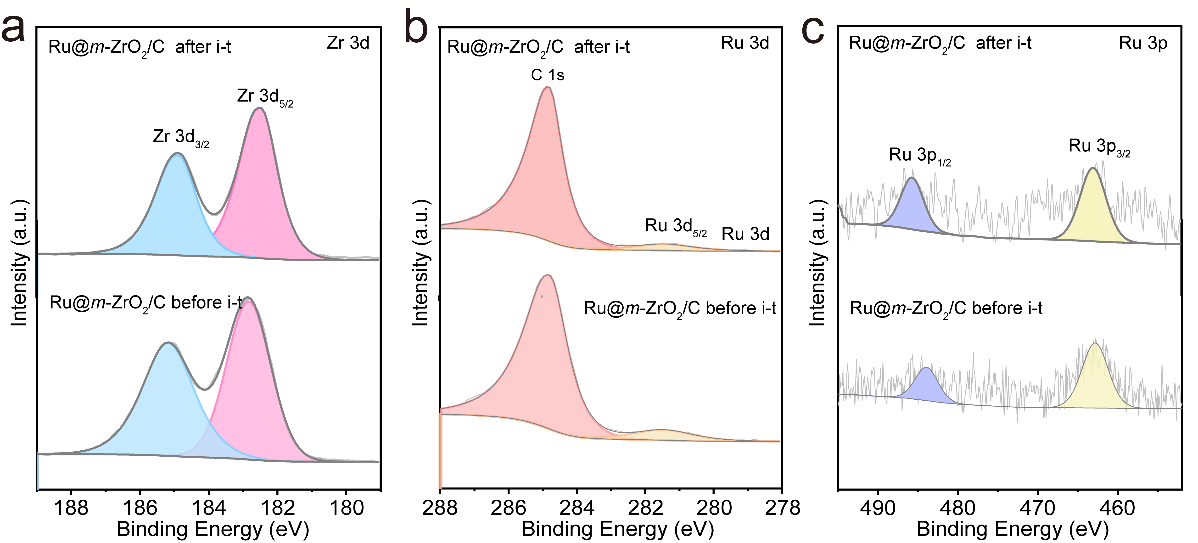


**Fig. S36** XPS spectra of Ru@*m*-ZrO_2_/C for **a** Zr 3d, **b** Ru 3d and **c** Ru 3p before and after i-t test in 1 M KOH.


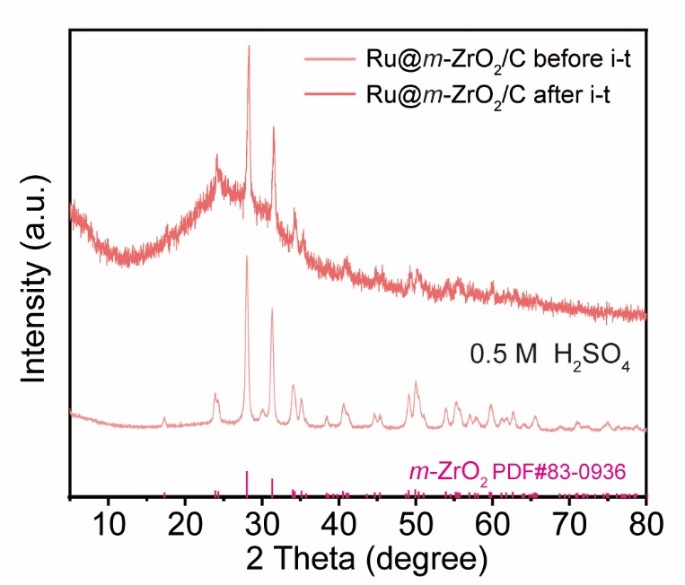


**Fig. S37** XRD patterns of Ru@*m*-ZrO_2_/C before and after i-t test in 0.5 M H_2_SO_4_.


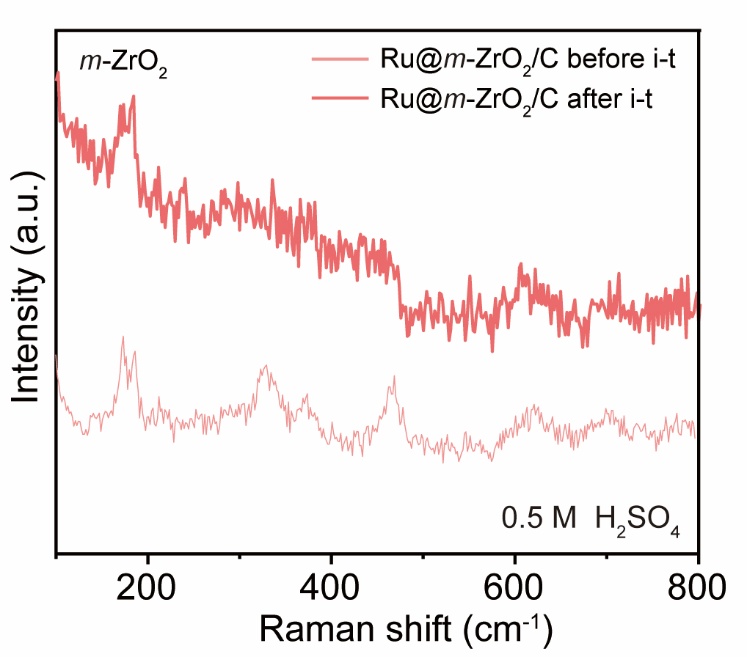


**Fig. S38** Raman spectra of Ru@*m*-ZrO_2_/C before and after i-t test in 0.5 M H_2_SO_4_.


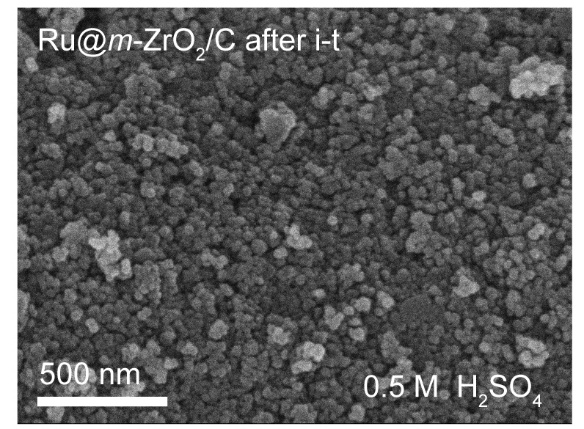


**Fig. S39** SEM image of Ru@*m*-ZrO_2_/C after i-t test in 0.5 M H_2_SO_4_.


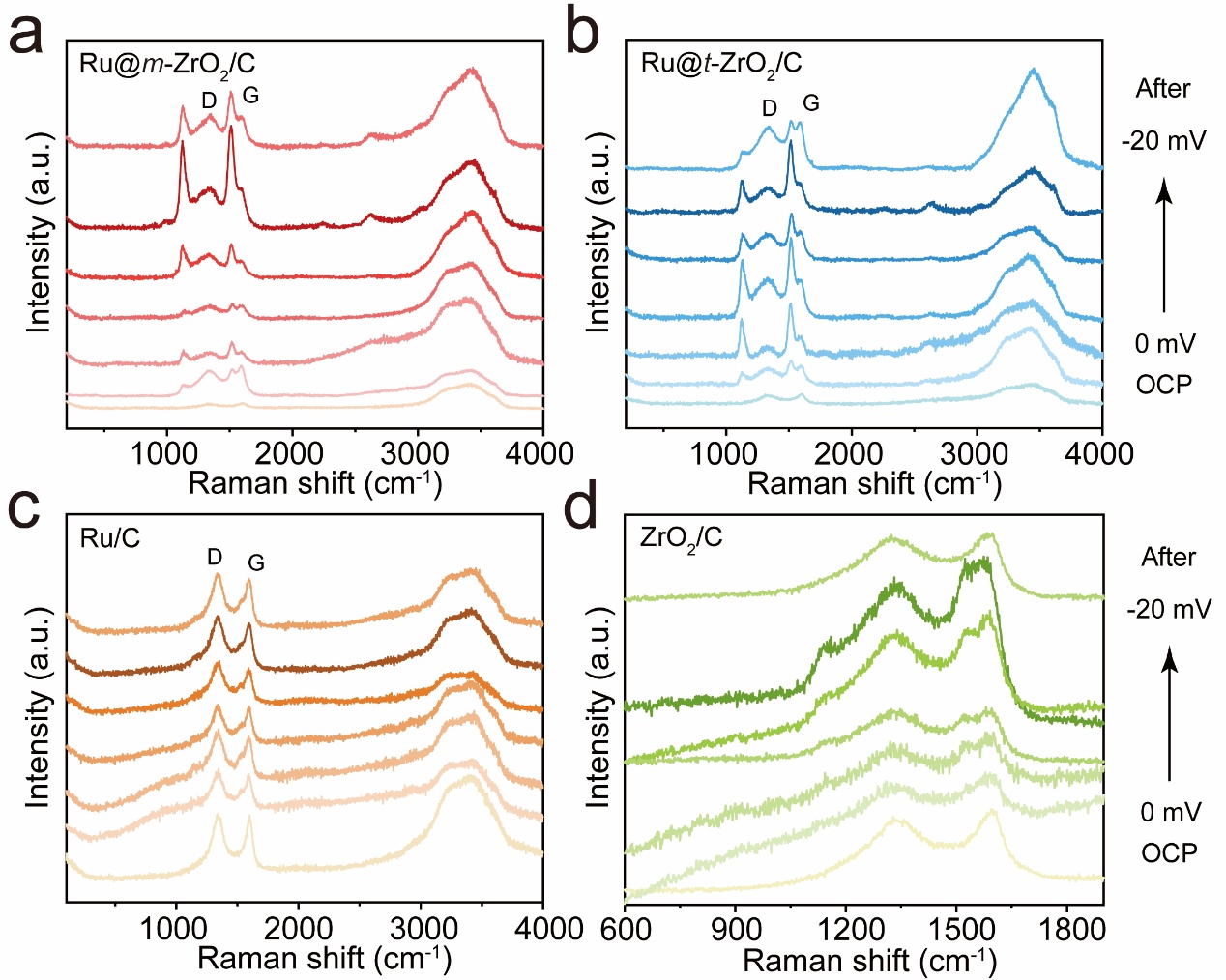


**Fig. S40** In-situ Raman spectra of **a** Ru@*m*-ZrO_2_/C, **b** Ru@*t*-ZrO_2_/C, **c** Ru/C and **d** ZrO_2_/C under applied potentials in 1 M KOH.


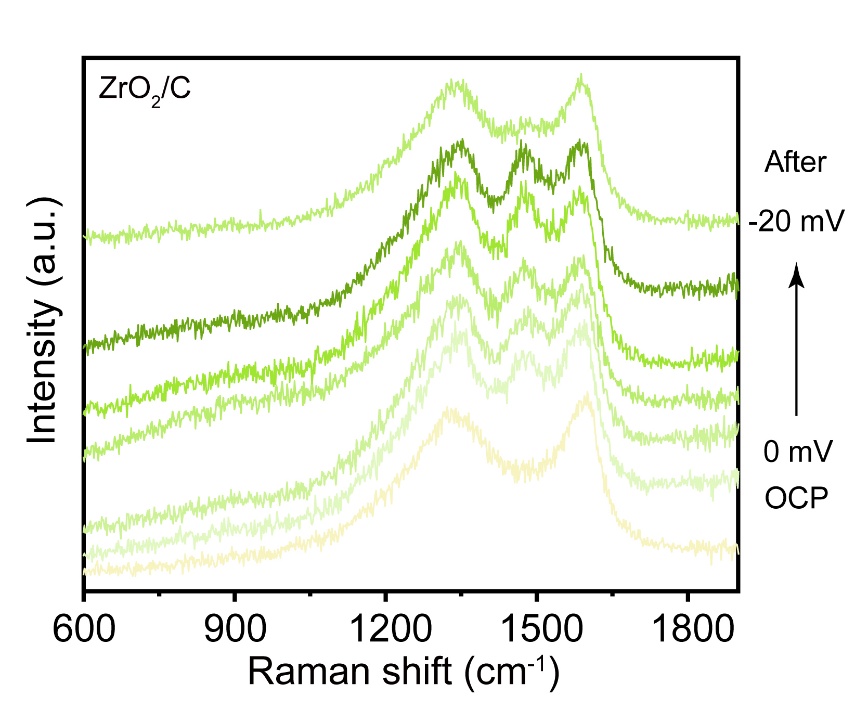


**Fig. S41** In-situ Raman spectra of ZrO_2_/C under applied potentials in 1 M KOD/D_2_O.


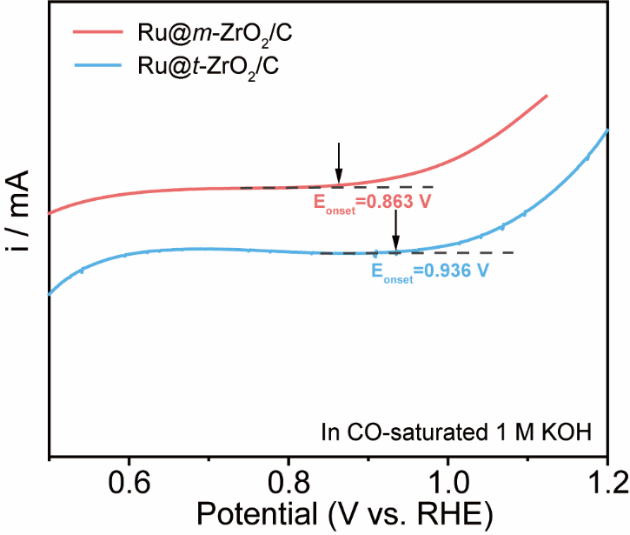


**Fig. S42** LSV curves of Ru@*m*-ZrO_2_/C and Ru@*t*-ZrO_2_/C in CO-saturated 1 M KOH.


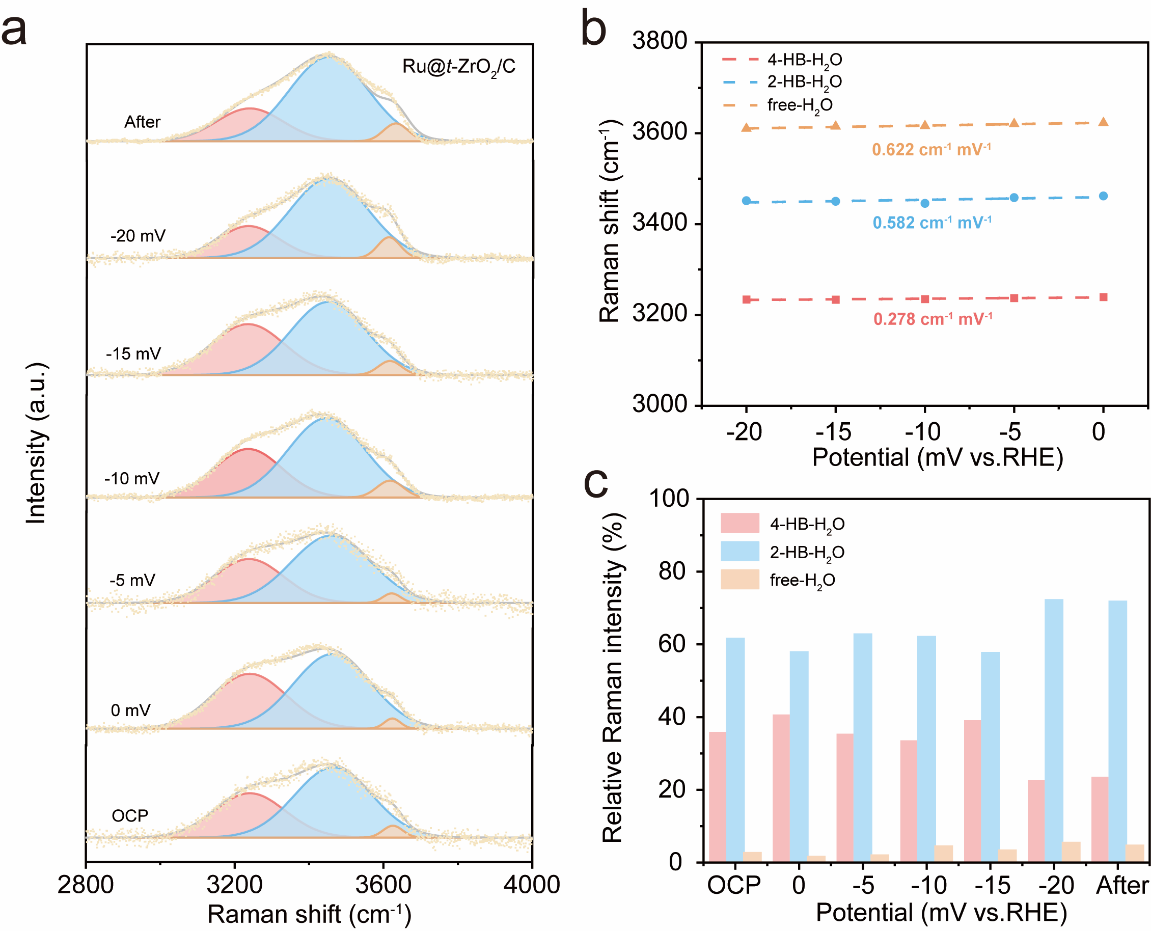


**Fig. S43** **a** In-situ Raman spectra of Ru@*m*-ZrO_2_/C in the range of 2800~4000 cm^-1^ in 1 M KOH. **b** The Stark slope and **c** population of 4-HB·H_2_O, 2-HB·H_2_O and free H_2_O derived from Raman spectra at different applied potential in **a**.


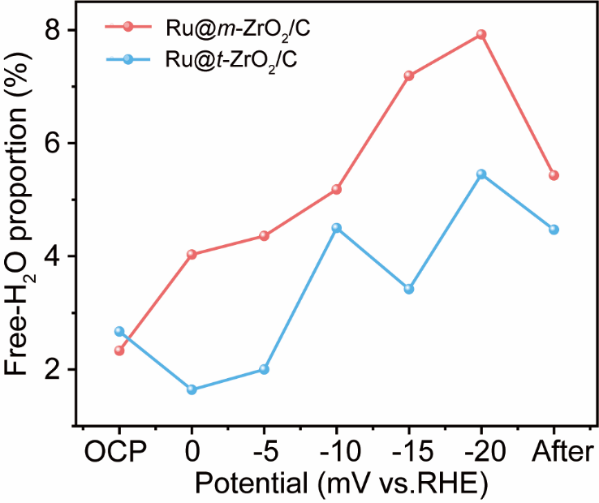


**Fig. S44** The proportions of free H_2_O against applied potentials on Ru@*m*-ZrO_2_/C and Ru@*t*-ZrO_2_/C.


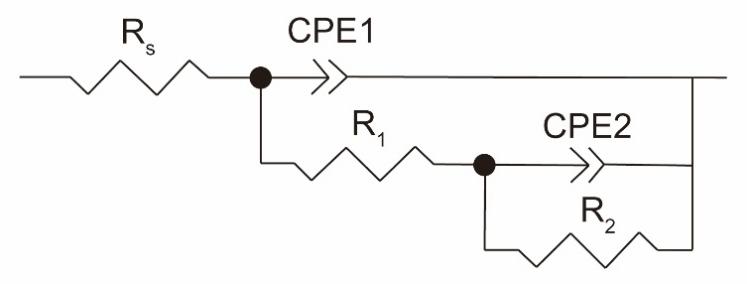


**Fig. S45** Equivalent circuit model for fitting EIS data in 1 M KOH.


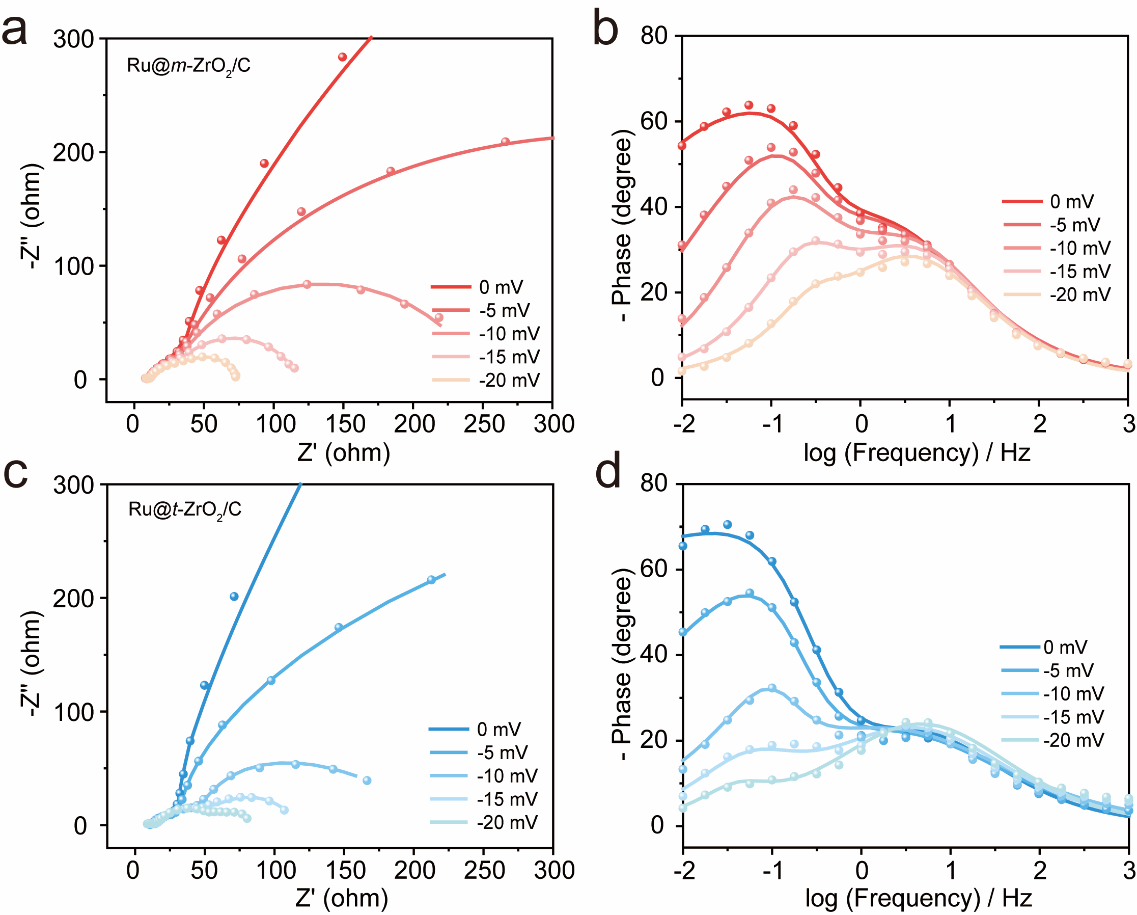


**Fig. S46** In-situ EIS measurement of **a, c** Nyquist and **b, d** Bode plots for **a, b** Ru@*m*-ZrO_2_/C and **c, d** Ru@*t*-ZrO_2_/C in 1 M KOH.


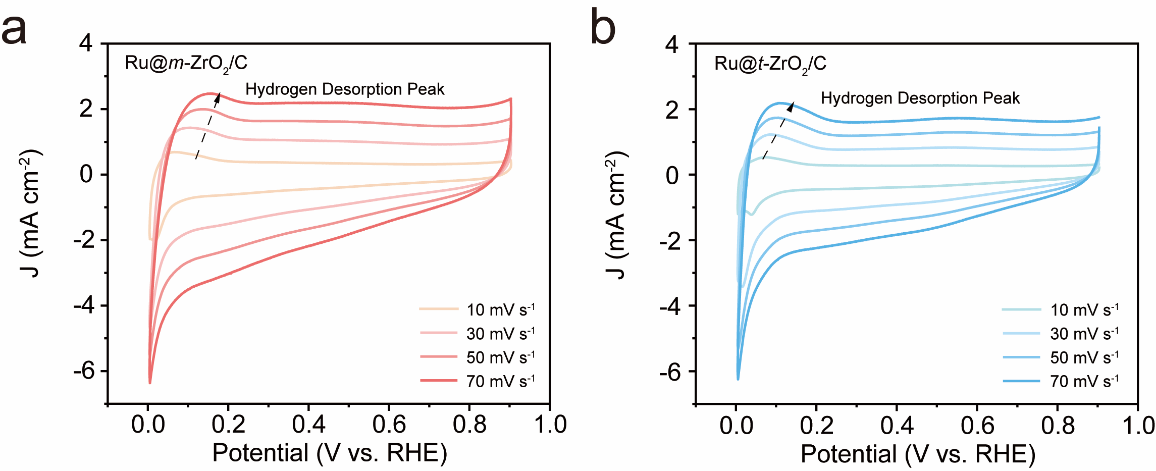


**Fig. S47** In-situ CV curves of **a** Ru@*m*-ZrO_2_/C and **b** Ru@*t*-ZrO_2_/C with the scan rate from 10 to 70 mV s^-1^ in 1 M KOH.


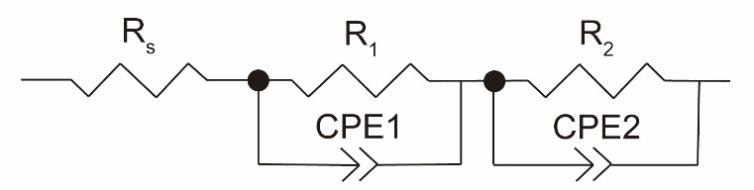


**Fig. S48** Equivalent circuit model for fitting EIS data in 0.5 M H_2_SO_4_.


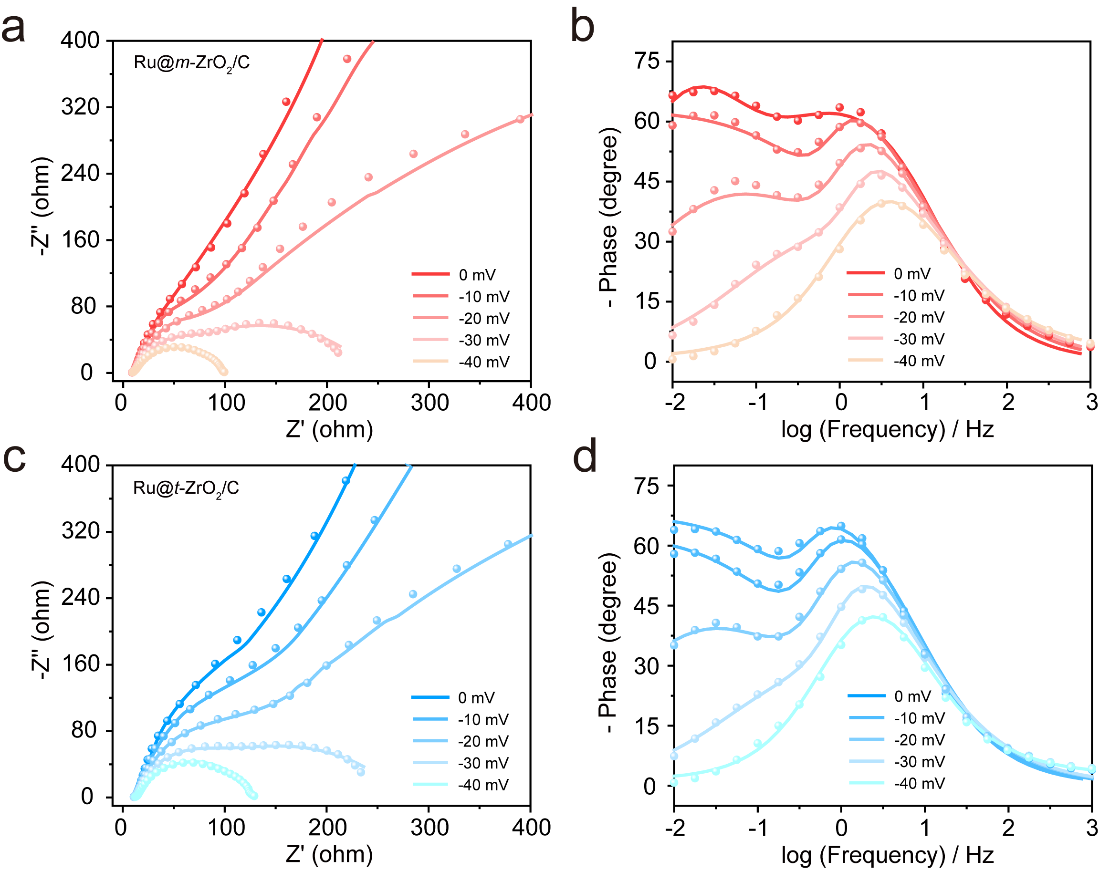


**Fig. S49** In-situ EIS measurement of **a, c** Nyquist and **b, d** Bode plots for **a, b** Ru@*m*-ZrO_2_/C and **c, d** Ru@*t*-ZrO_2_/C in 0.5 M H_2_SO_4_.


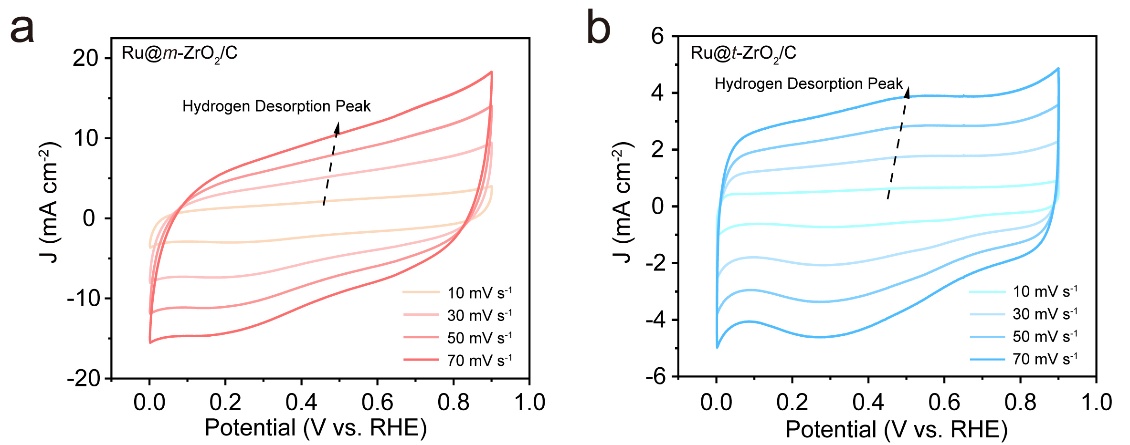


**Fig. S50** In-situ CV curves of **a** Ru@*m*-ZrO_2_/C and **b** Ru@*t*-ZrO_2_/C with the scan rate from 10 to 70 mV s^-1^ in 0.5 M H_2_SO_4_.


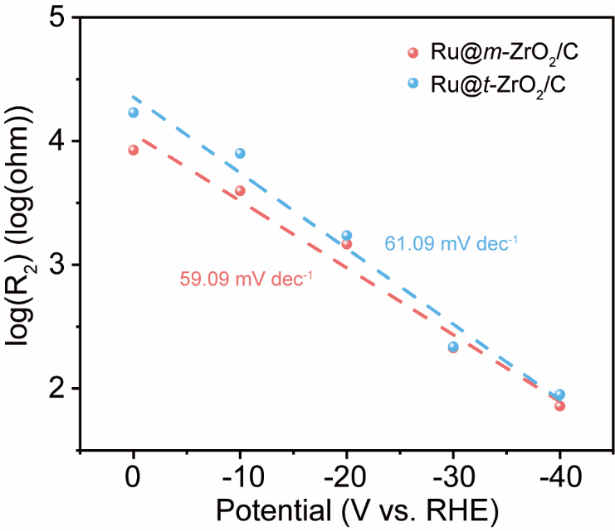


**Fig. S51** Tafel slope of Ru@*m*-ZrO_2_/C and Ru@*t*-ZrO_2_/C estimated from R_2_ in 0.5 M H_2_SO_4_.


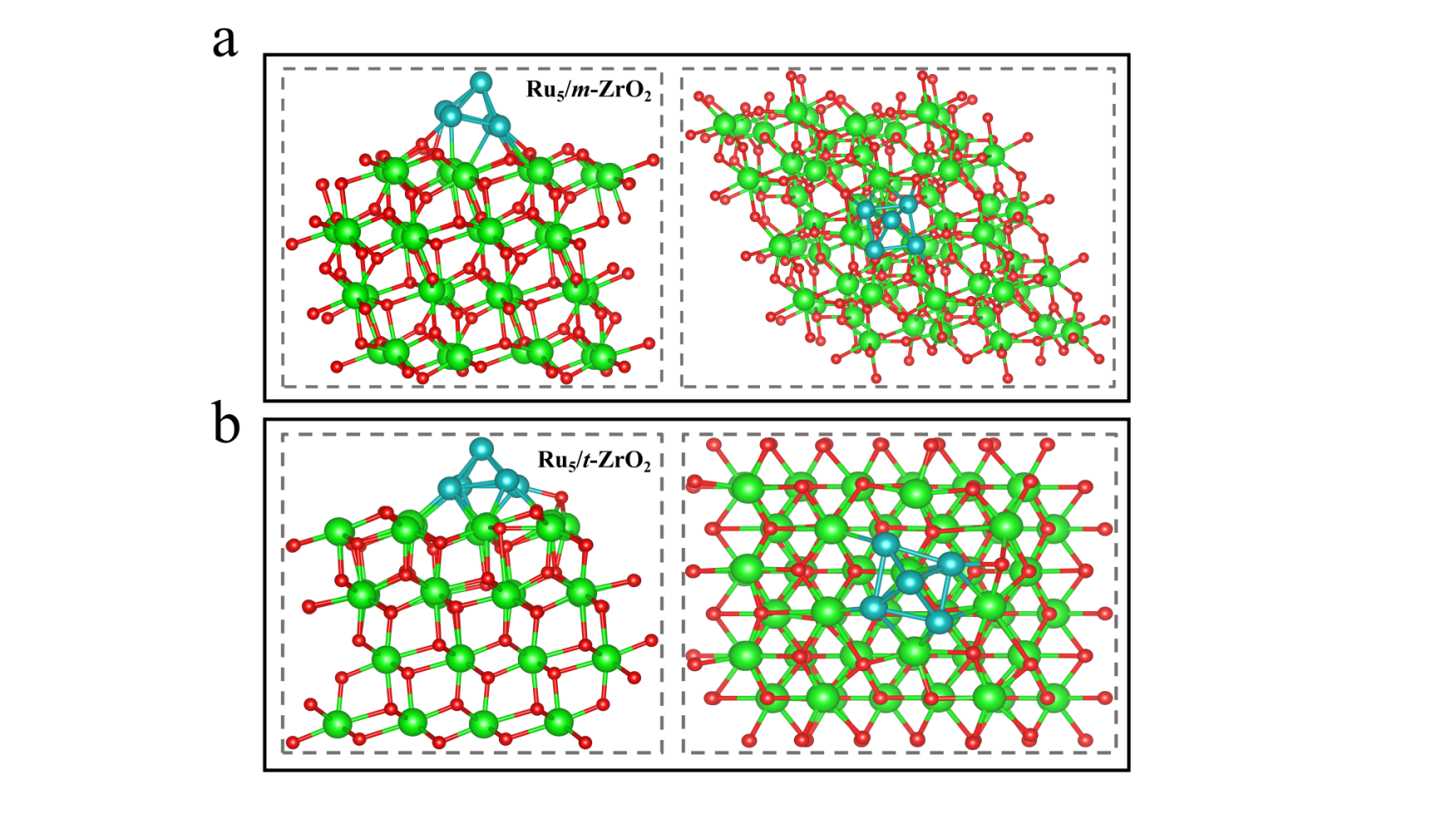


**Fig. S52** Optimized structures of **a** Ru_5_/*m*-ZrO_2_ and **b** Ru_5_/*t*-ZrO_2_.


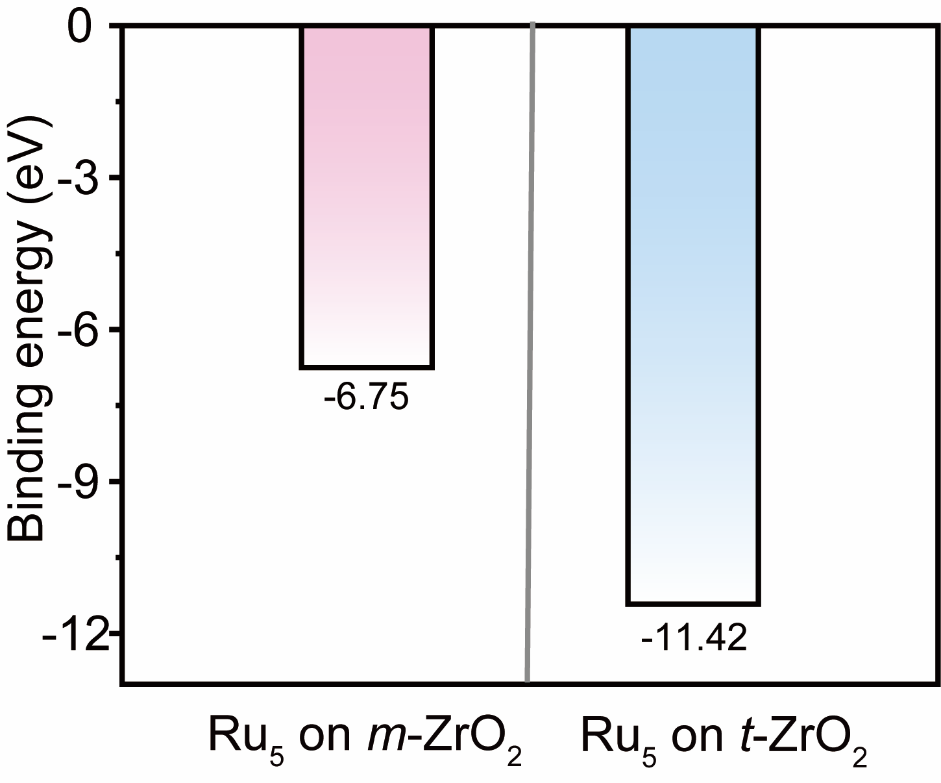


**Fig. S53** Binding energy of Ru_5_ cluster on *m*-ZrO_2_ and *t*-ZrO_2_.





**Fig. S54** PDOS profiles of Zr d orbital for Ru_5_/*m*-ZrO_2_ and Ru_5_/*t*-ZrO_2_.


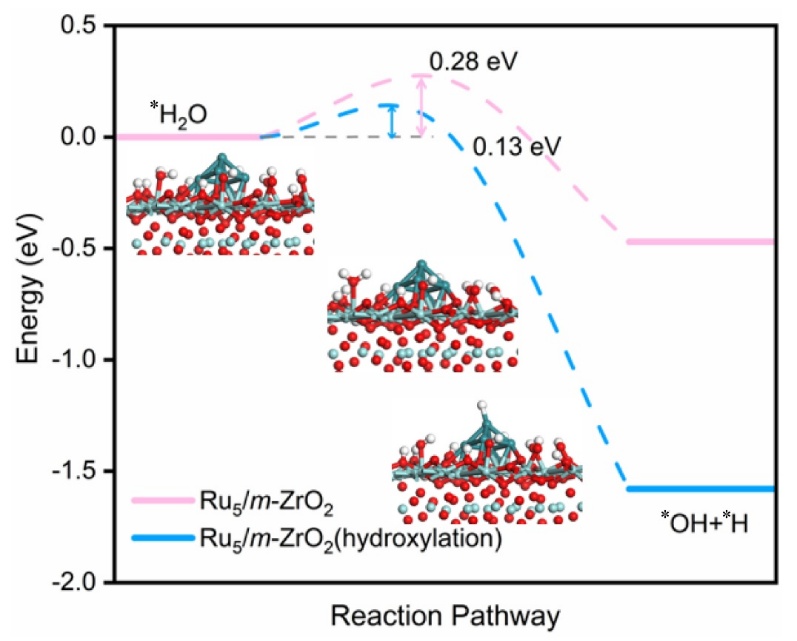


**Fig. S55** Adsorption energy and energy barrier for surface hydroxylation of Ru_5_/*m*-ZrO_2_.


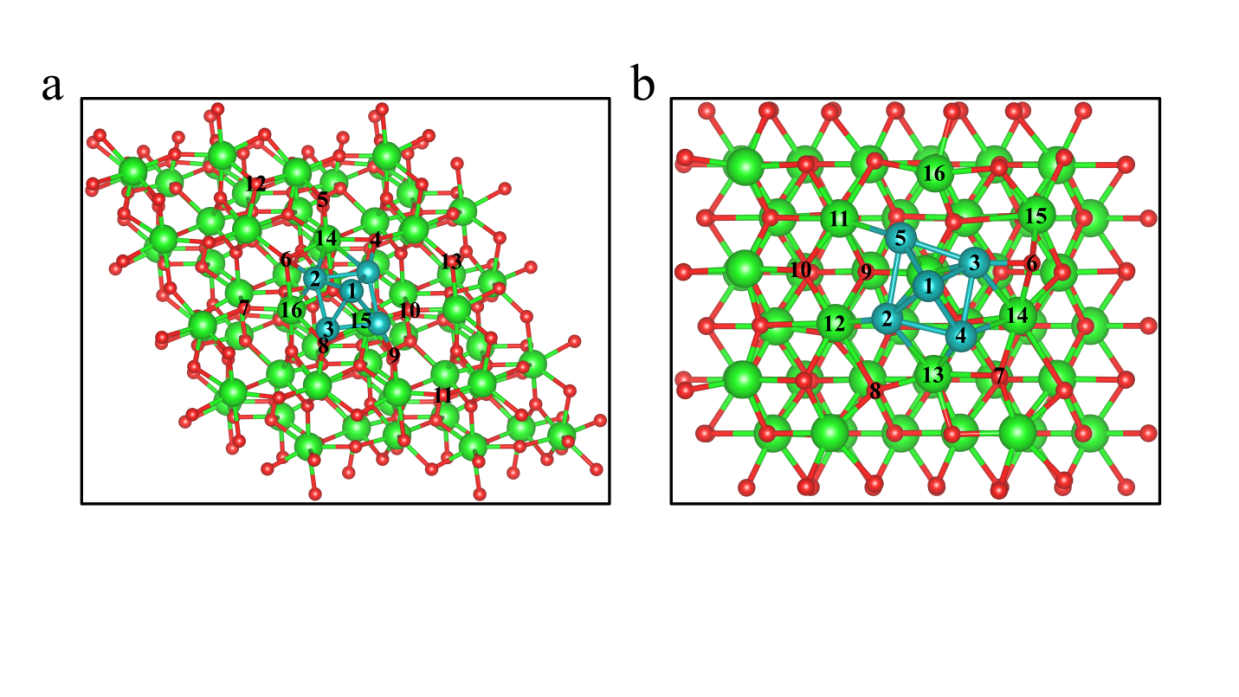


**Fig. S56** Selected hydrogen adsorption sites on **a** Ru_5_/*m*-ZrO_2_ and **b** Ru_5_/*t*-ZrO_2_.


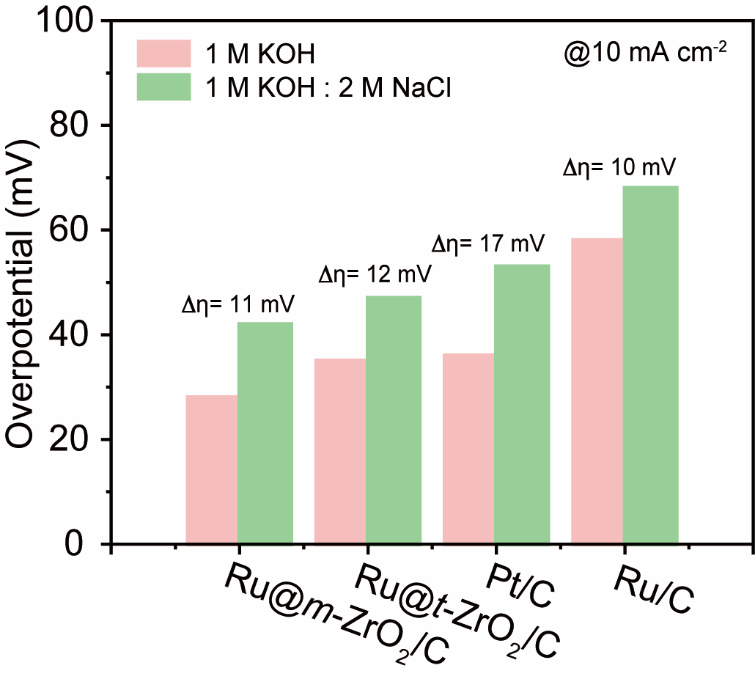


**Fig. S57** Comparison of overpotential for Ru@*m*-ZrO_2_/C, Ru@*t*-ZrO_2_/C, Pt/C and Ru/C in 1 M KOH and 1 M KOH + 2 M NaCl.


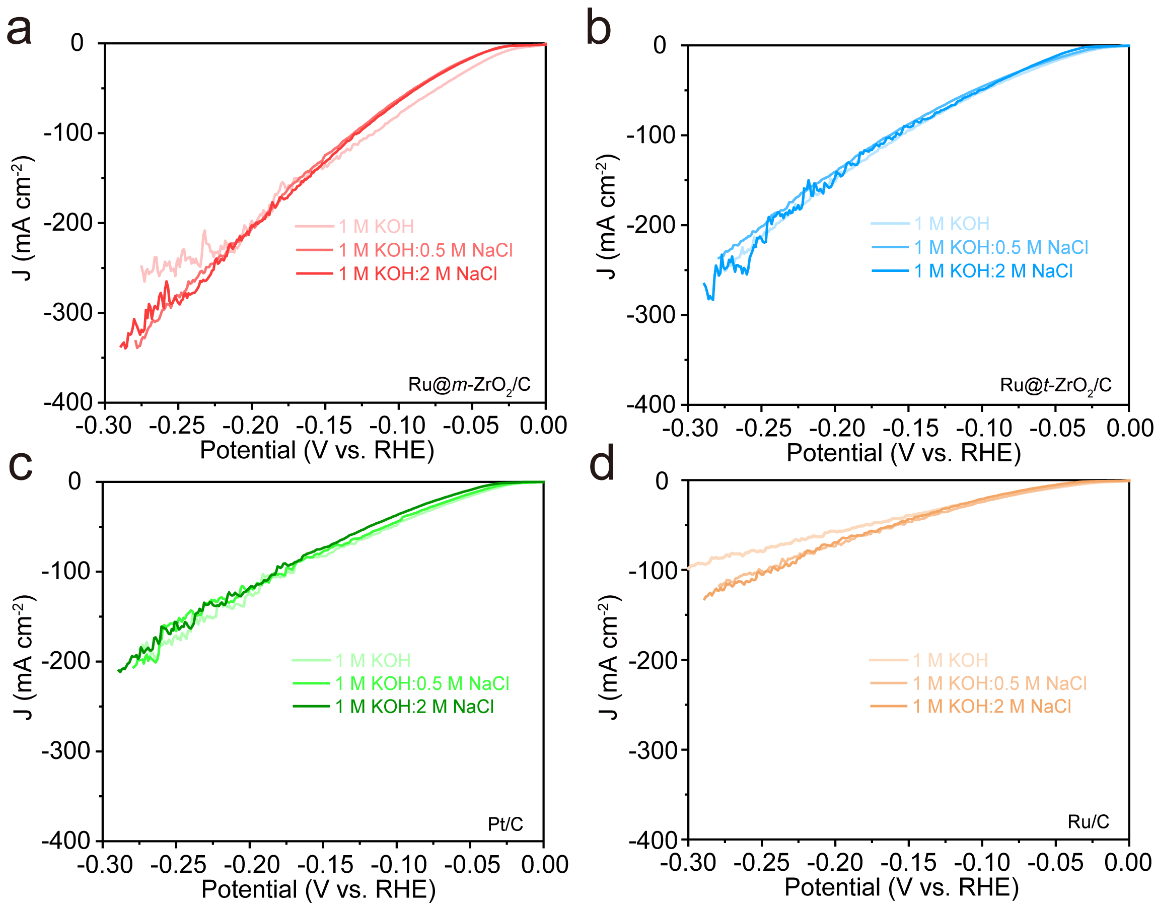


**Fig. S58** LSV curves of **a** Ru@*m*-ZrO_2_/C, **b** Ru@*t*-ZrO_2_/C, **c** Pt/C and **d** Ru/C in 1 M KOH + x M NaCl (x = 0, 0.5, 2).


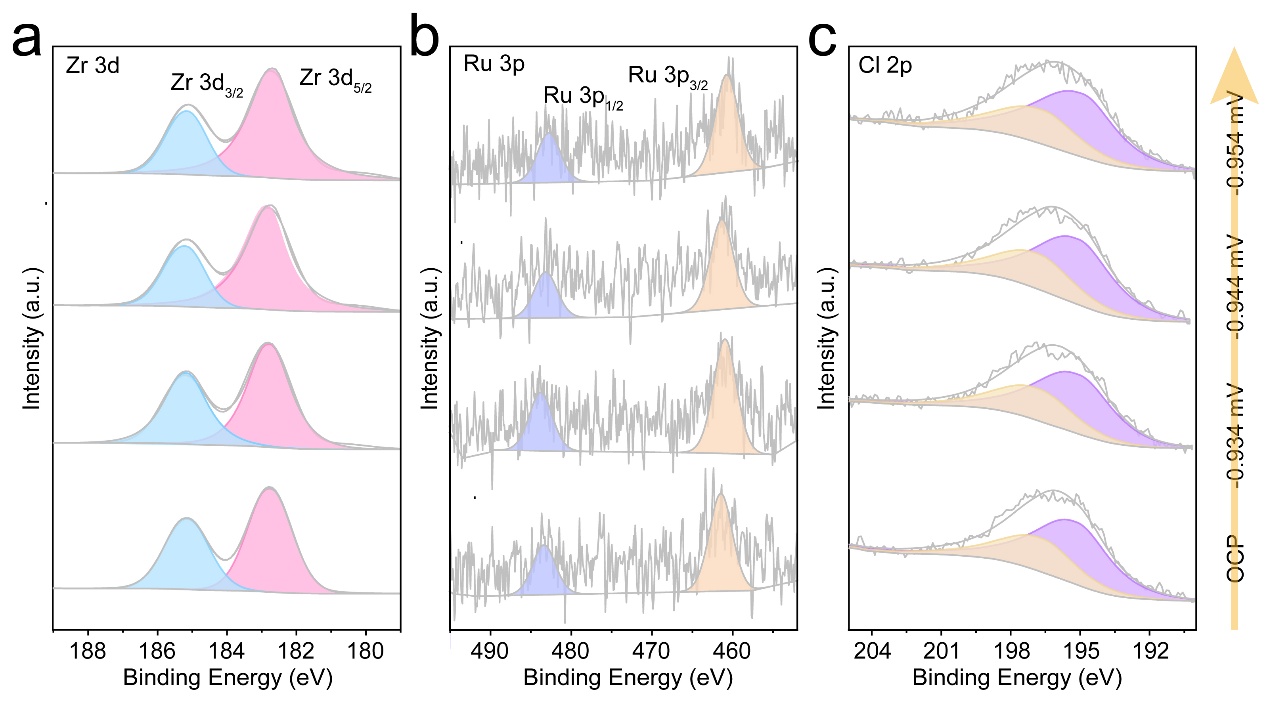


**Fig. S59** XPS spectra of Ru@*m*-ZrO_2_/C for **a** Zr 3d, **b** Ru 3p and **c** Cl 2p in 1 M KOH + 2 M NaCl.


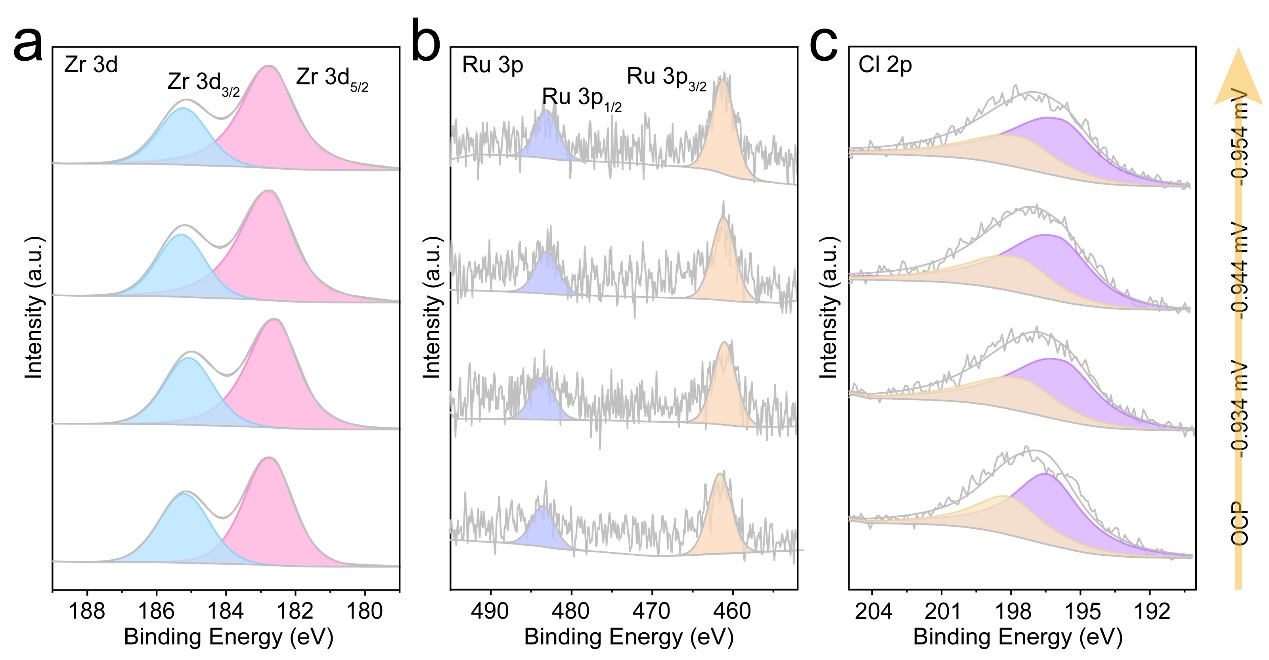


**Fig. S60** XPS spectra of Ru@*t*-ZrO_2_/C for **a** Zr 3d, **b** Ru 3p and **c** Cl 2p in 1 M KOH + 2 M NaCl.


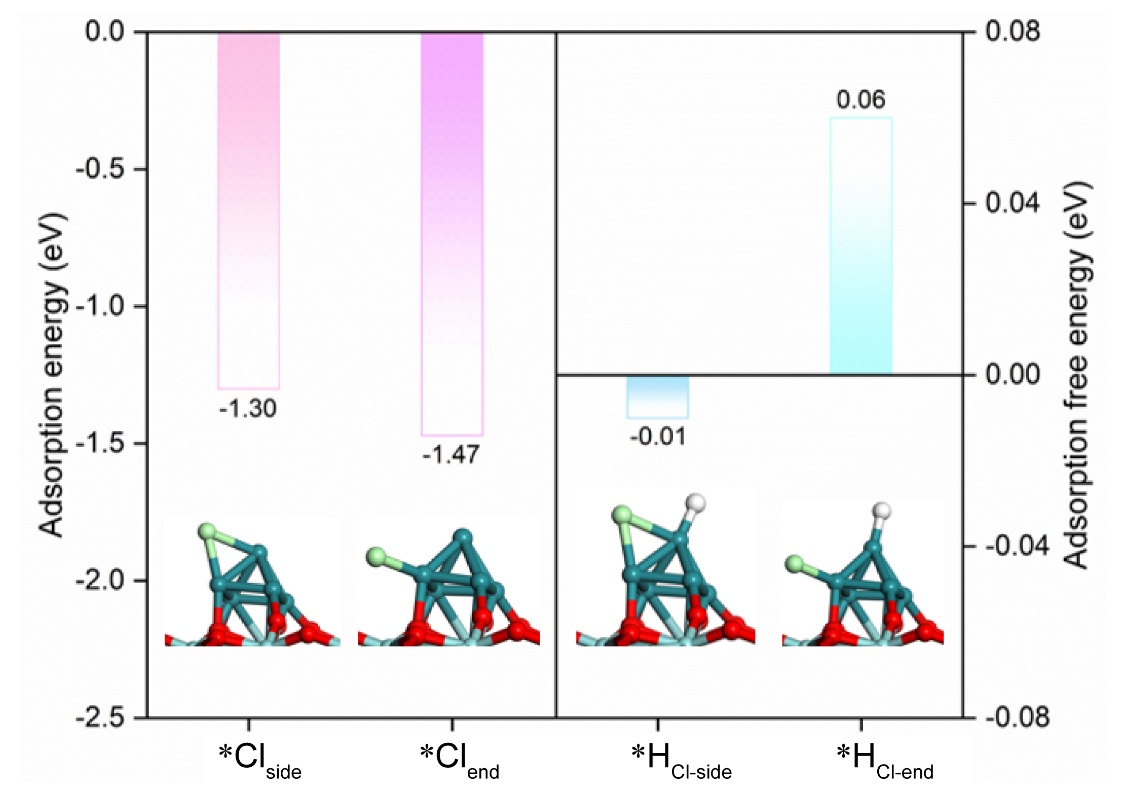


**Fig. S61** Adsorption energy of Cl⁻ on Ru_5_/*m*-ZrO_2_.


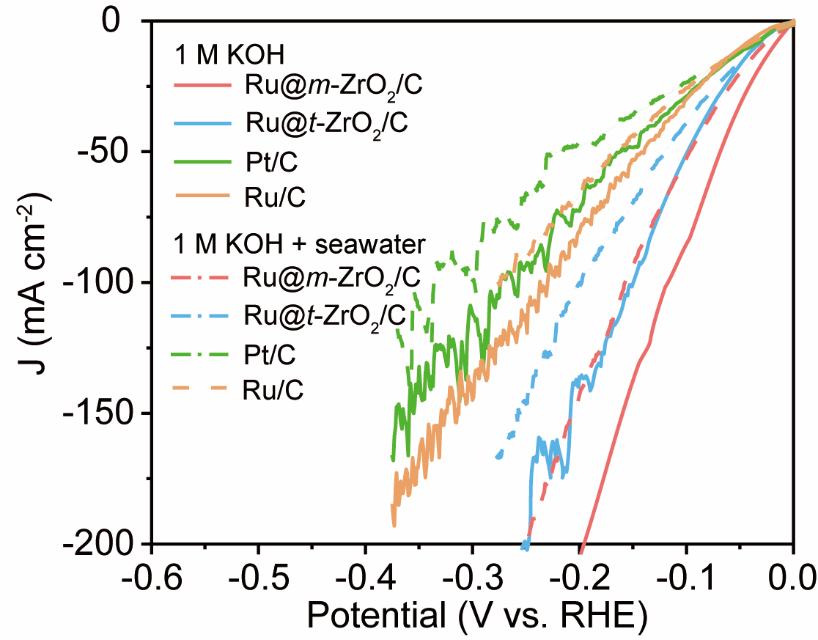


**Fig. S62** LSV curves of Ru@*m*-ZrO_2_/C, Ru@*t*-ZrO_2_/C, Pt/C and Ru/C in M KOH and 1 M KOH + simulated seawater.


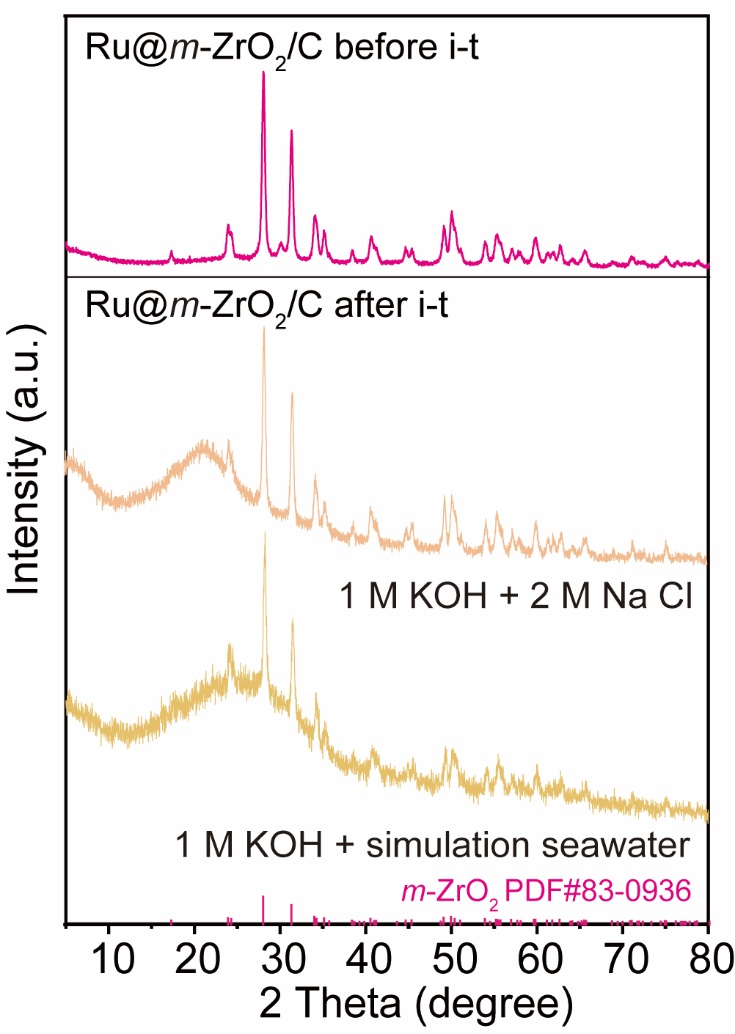


**Fig. S63** XRD patterns of Ru@*m*-ZrO_2_/C before and after i-t test in 1 M KOH + 2 M NaCl and 1 M KOH + simulated seawater, respectively.


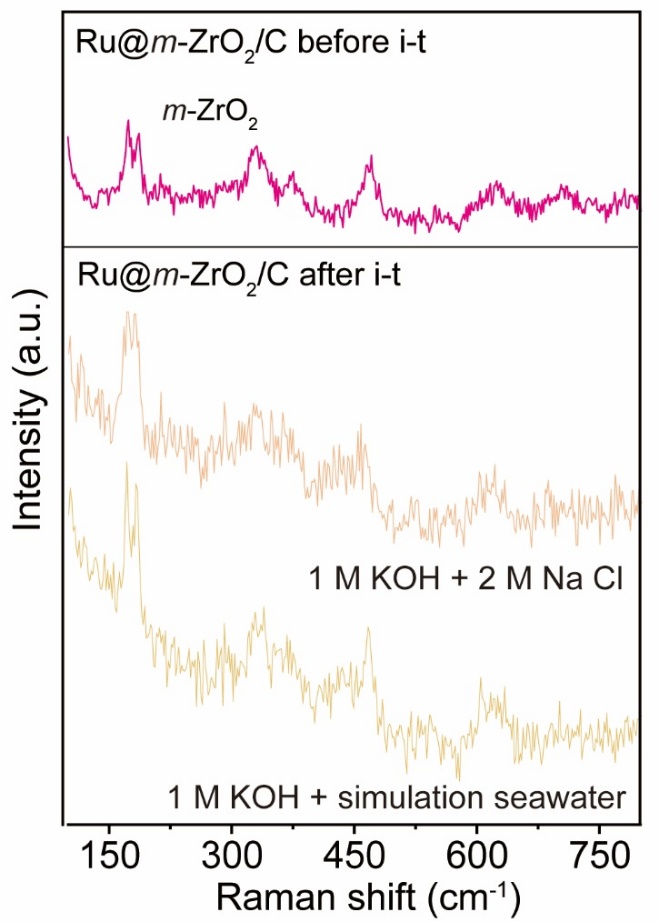


**Fig. S64** Raman spectra of Ru@*m*-ZrO_2_/C before and after i-t test in 1 M KOH + 2 M NaCl and 1 M KOH + simulated seawater, respectively.


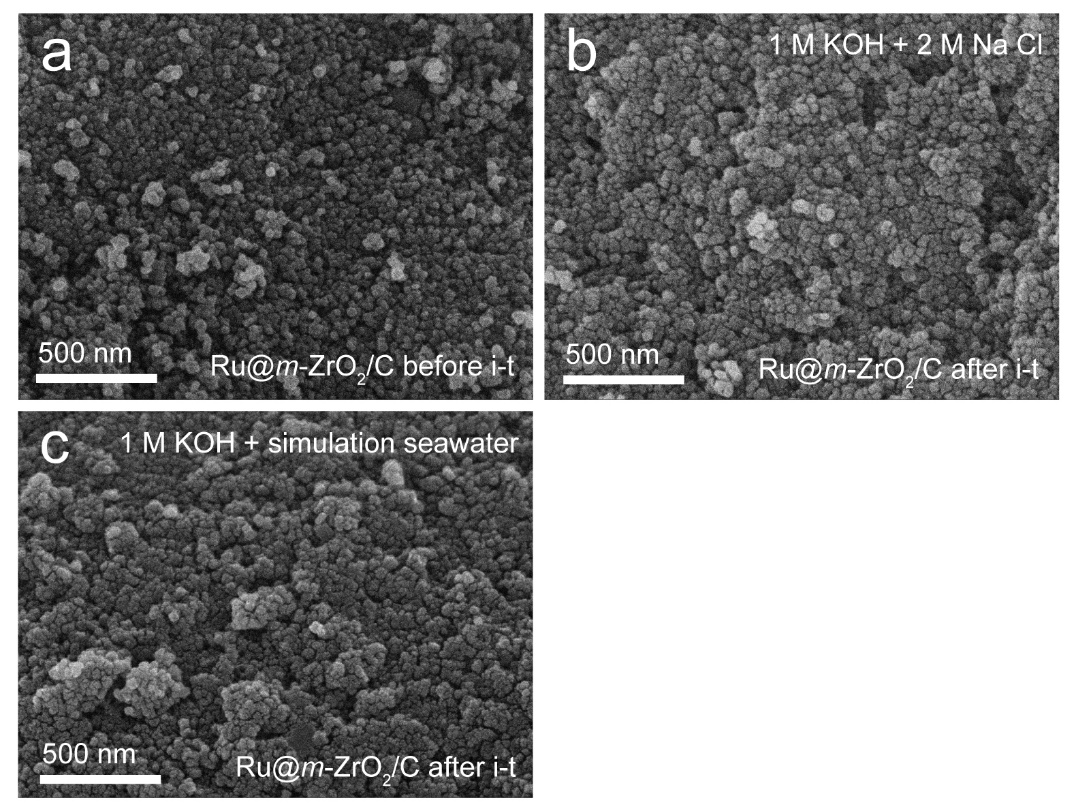


**Fig. S65** SEM images of Ru@*m*-ZrO_2_/C **a** before, **b** after i-t test in 1 M KOH + 2 M NaCl and **c** after i-t test in 1 M KOH + simulated seawater.

**
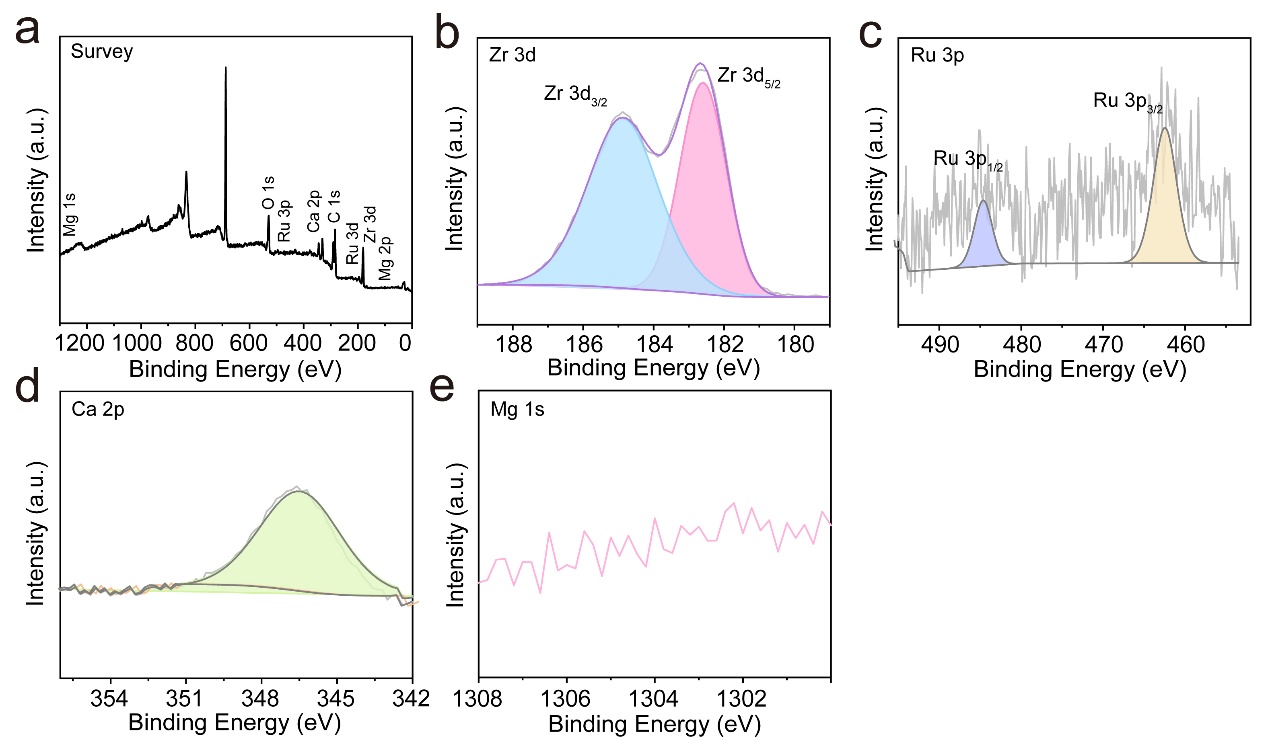
**

**Fig. S66** XPS spectra of Ru@*m*-ZrO_2_/C for **a** survey, **b** Zr 3d, **c** Ru 3p, **d** Ca 2p and **e** Mg 1s after i-t test in 1 M KOH + simulated seawater.

**
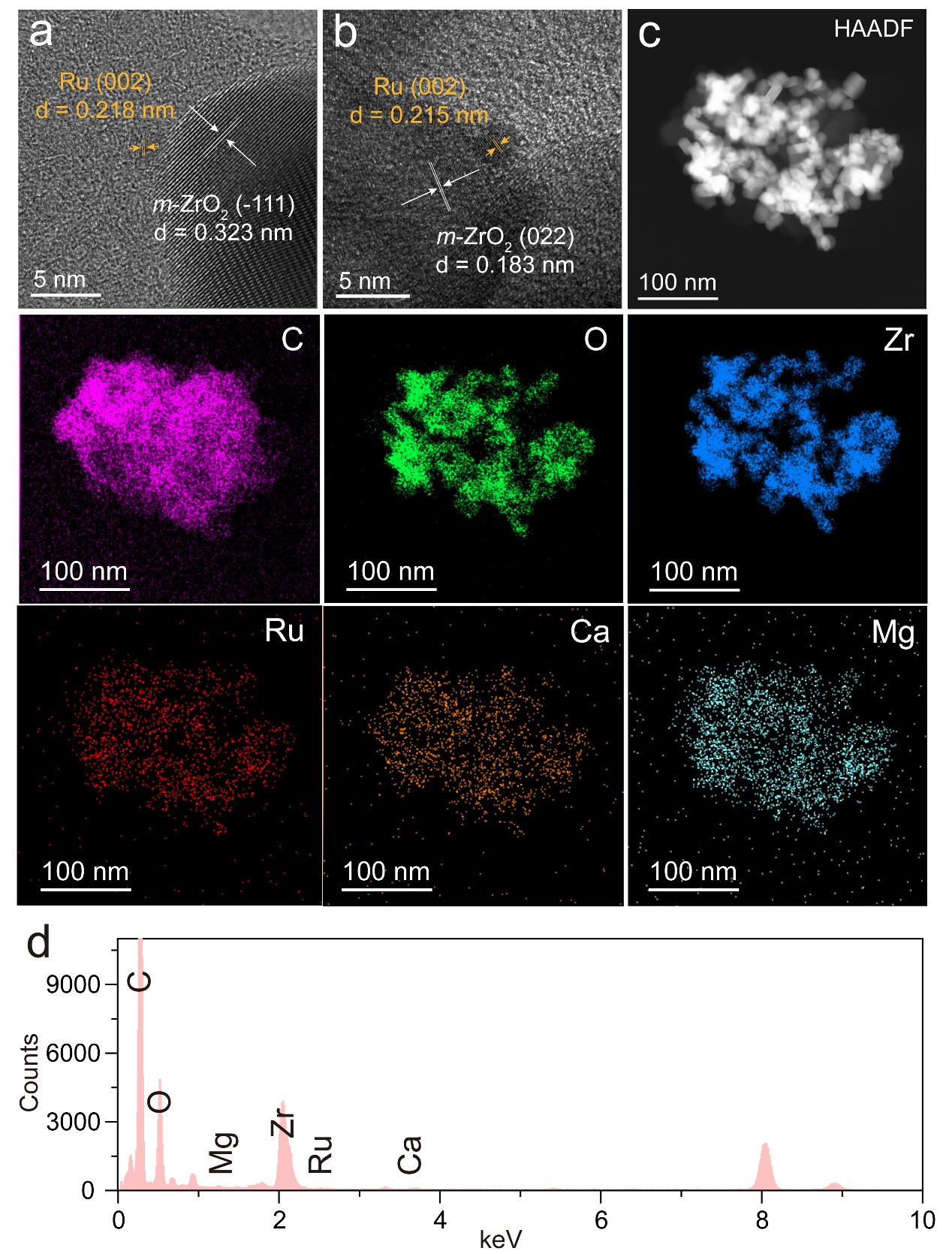
**

**Fig. S67** **a**, **b** HRTEM images, **c** HAADF image and the corresponding elemental mapping for C (pink), O (green), Zr (blue), Ru (red), Ca (orange) and Mg (cyan), **d** EDS of Ru@*m*-ZrO_2_/C after i-t test in 1 M KOH + simulated seawater.

**Table S1** The content of Ru in Ru@*m*-ZrO_2_/C and Ru@*t*-ZrO_2_/C determined by ICP-AES

| Sample | Ru content (%) |
| --- | --- |
| Ru@*m*-ZrO_2_/C | 0.72 |
| Ru@*t*-ZrO_2_/C | 1.02 |

**Table S2** EXAFS fitting parameters at the Zr K-edge for various samples

| Sample | Coordination | CN | R(Å) | σ^2^(×1 0^-3^Å^2^) | ΔE(eV) | R-factor |
| --- | --- | --- | --- | --- | --- | --- |
| Zr foil | Zr-Zr | 12 | 2.71 | -- | -- | -- |
| Ru@*m*-ZrO_2_/C | Zr-O | 8.1 | 2.15 | 9.3 | -4.5 | 1.60% |
|  | Zr-Zr | 6.3 | 3.48 | 6.0 | -4.5 |  |
| Ru@*t*-ZrO_2_/C | Zr-O | 4.4 | 2.09 | 3.0 | -5.5 | 0.64% |
|  | Zr-O | 2.4 | 2.30 | 4.6 | -5.5 |  |
|  | Zr- Zr | 12.3 | 3.62 | 7.3 | -5.5 |  |

CN: coordination numbers, R: bond distance; σ^2^: Debye-Waller factors, ΔE_0_: the inner potential correction. R factor reflects the goodness of fit.

**Table S3** EXAFS fitting parameters at the Ru K-edge for various samples

| Sample | Coordination | CN | R(Å) | σ^2^(×10^-3^Å^2^) | ΔE(eV) | R-factor |
| --- | --- | --- | --- | --- | --- | --- |
| Ru foil | Ru-Ru | 12 | 2.71 | -- | -- | -- |
| RuO_2_ | Ru-O | 6 | 2.01 | -- | -- | -- |
| Ru@*m*-ZrO_2_/C | Ru-O | 2.7 | 1.94 | 4.3 | -0.4 | 0.91% |
|  | Ru-Ru | 2.5 | 2.69 | 5.9 | -0.4 |  |
| Ru@*t*-ZrO_2_/C | Ru-O | 1.9 | 1.97 | 4.8 | -3.4 | 0.65% |
|  | Ru-Ru | 4.1 | 2.67 | 5.1 | -3.4 |  |

CN: coordination numbers, R: bond distance; σ^2^: Debye-Waller factors, ΔE_0_: the inner potential correction. R factor reflects the goodness of fit.

**Table S4** A series of Ru@*m*-ZrO_2_/C and Ru@*t*-ZrO_2_/C catalysts with different Ru cluster sizes

| the amount of RuCl_3_ precursor (mmol) | Ru cluster sizes on the catalyst (nm) |
| --- | --- |
| 0.30 | Ru@*m*-ZrO_2_/C (1.38 ± 0.06) |
| 0.28 | Ru@*m*-ZrO_2_/C (1.42 ± 0.06) |
| 0.31 | Ru@*m*-ZrO_2_/C (2.15 ± 0.05) |
| 0.34 | Ru@*m*-ZrO_2_/C (3.32 ± 0.65) |
| 0.30 | Ru@*t*-ZrO_2_/C (1.45 ± 0.07) |
| 0.31 | Ru@*t*-ZrO_2_/C (1.70 ± 0.04) |
| 0.34 | Ru@*t*-ZrO_2_/C (3.30 ± 0.07) |

**Table S5** The content of a series of Ru@*m*-ZrO_2_/C and Ru@*t*-ZrO_2_/C catalysts determined by ICP-AES.

| the amount of RuCl_3_ precursor (mmol) | Ru content on the catalyst (wt%) |
| --- | --- |
| 0.30 | Ru@*m*-ZrO_2_/C (0.72) |
| 0.27 | Ru@*m*-ZrO_2_/C (0.76) |
| 0.29 | Ru@*m*-ZrO_2_/C (1.43) |
| 0.32 | Ru@*m*-ZrO_2_/C (0.93) |
| 0.30 | Ru@*t*-ZrO_2_/C (1.02) |
| 0.27 | Ru@*t*-ZrO_2_/C (0.89) |
| 0.29 | Ru@*t*-ZrO_2_/C (1.35) |
| 0.32 | Ru@*t*-ZrO_2_/C (1.57) |

**Table S6** Comparison of the TOF for Ru@*m*-ZrO_2_/C, Ru@*t*-ZrO_2_/C, Pt/C and Ru/C in 1 M KOH

| TOF (H_2_ s^-1^) @ η | Ru@*m*-ZrO_2_/C | Ru@*t*-ZrO_2_/C | Pt/C | Ru/C |
| --- | --- | --- | --- | --- |
| η_100_ | 0.04086 | 0.0226 | 0.03919 | 0.00936 |
| η_200_ | 0.12776 | 0.06226 | 0.11327 | 0.02564 |
| Η_300_ | 0.24587 | 0.1169 | 0.20365 | 0.04267 |

**Table S7** HER performance comparison on the η_10_, Tafel slopes, mass activities, TOF and R_ct_ of Ru@*m*-ZrO_2_/C, Ru@*t*-ZrO_2_/C, Pt/C and Ru/C in 1 M KOH

|  | Ru@*m*-ZrO_2_/C | Ru@*t*-ZrO_2_/C | Pt/C | Ru/C |
| --- | --- | --- | --- | --- |
| η_10_ (mV) | 28 | 35 | 36 | 48 |
| Tafel slope (mV dec^-1^) | 31.4 | 42.2 | 51.0 | 69.9 |
| Mass activity (A mg^-1^) @ η = 50 mV | 2.72 | 1.22 | 0.06 | 0.15 |
| TOF (H_2_ s^-1^) @ η = 100 mV (CO-stripping) | 0.0409 | 0.0226 | 0.0392 | 0.0094 |
| R_ct_ (Ω) | 20.4 | 24.6 | 30.4 | 33.1 |

**Table S8** Comparison of the HER activity of the Ru@*m*-ZrO_2_/C with other reported catalysts in 1 M KOH.

| Catalysts | η_10_ (mV) | Tafel slope (mV dec^-1^) | Reference |
| --- | --- | --- | --- |
| Ru@*m*-ZrO_2_/C | 28 | 31.4 | This work |
| Ru@*t*-ZrO_2_/C | 35 | 42.2 | This work |
| 20% Pt/C | 36 | 51 | This work |
| 5% Ru/C | 48 | 69.9 | This work |
| Pt/C_60_ | 25 | 55 | Nat. Commun., 2023, 14, 2460. |
| Ru/HMCs-500 | 26.93 | 41.26 | J. Mater. Chem. A, 2023, 11(7), 3524-3534. |
| RuNi-alloy@SC | 34 | 55 | Chem. Eng. J., 2021, 417, 129319. |
| MSOR_x_ | 43 | 63.1 | Adv. Funct. Mater., 2023, 33, 2210939. |
| Rh/NiFeRh-LDH | 58 | 81.3 | Nano Lett., 2020, 20, 136. |
| 5% Ru-Ti_3_C_2_T_x_ | 46 | 37.6 | J. Alloy. Compd., 2023, 960, 170764. |
| Ru/Co-CAT/CC | 38 | 32.1 | Adv. Energy Mater., 2023, 13(20), 2204177. |
| Ru-CrN/NC | 53 | 59 | J. Mater. Chem. A, 2024, 12, 8291-8301. |
| Ru@TiO_2_ | 62 | 70 | Chem. Front., 2023, 10, 3852-3859. |
| NiRu-OH | 38 | 39 | Appl. Catal. B Environ., 2020, 269, 118824. |
| CoMnP/NF | 53.9 | 63 | Int. J. Hydrogen Energy, 2022, 47(26), 12927-12936. |
| Pt@Mo-S-Ni-CNTs | 61.2 | 52.3 | Carbon, 2024, 224, 119061. |
| Pt_1_/Ni(OH)_2_/C | 55 | 52.1 | Energy Environ. Sci., 2023, 16, 1035-1048. |
| Ir/WO_x_/rGO | 53 | 62 | Energy Environ. Mater., 2021, 4, 681-686. |
| Ru@1T-MoS_2_ MXene | 44 | 47 | Adv. Funct. Mater., 2023, 33(13), 2212514. |
| Pt_1_/NMHCS | 42 | 38 | Adv. Mater., 2021, 33, 2008599. |
| Ru SAs-NiP | 57 | 75 | Nano Energy, 2021, 80, 105467. |
| SA-Ru-MoS_2_ | 76 | 21 | Small Methods, 2019, 3, 1900653. |
| Ru-Fe-P | 44 | 80 | Appl. Catal. B Environ., 2020, 263 118324. |
| MoP/CNT | 86 | 73 | Adv. Funct. Mater., 2018, 28, 1706523. |
| Ru-NPs/SAs@N-TC | 97 | 58 | Adv. Funct. Mater., 2020, 30, 2003007. |

**Table S9** Comparison of the HER activity of the Ru@*m*-ZrO_2_/C with other reported catalysts in 0.5 M H_2_SO_4_.

| Catalysts | η_10_ (mV) | Tafel slope  (mV dec^-1^) | Reference |
| --- | --- | --- | --- |
| Ru@*m*-ZrO_2_/C | 56 | 49.4 | This work |
| Ru@*t*-ZrO_2_/C | 67 | 62.0 | This work |
| 20% Pt/C | 33 | 16.6 | This work |
| 5% Ru/C | 82 | 81.9 | This work |
| Ru-MoO_3-x_/Mo_2_AlB_2_ | 38 | 57.1 | Angew. Chem. Int. Ed., 2025, 64(24), e202504084 |
| Co_5_Ru_1_@NCNT/PF | 45 | 64 | Adv. Sci., 2022, 9, 2200010. |
| Ru-CrN/NC | 53 | 59 | J. Mater. Chem. A, 2024, 12, 8291. |
| NCAG/Ru-3 | 65 | 68.8 | Chem. Eng. J, 2022, 442, 136337. |
| RuS_x_/S-GO | 31 | 40 | Small, 2019, 15, 1904043. |
| s-RuS_2_/S-rGO | 69 | 64 | ACS Appl. Mater. Interfaces, 2018, 10 (40), 34098-34107. |
| Cu@Cu_3_P-Ru/CCG-500 | 102.5 | 63 | Appl. Catal. B Environ., 2023, 326, 122402. |
| CoRu/NC-700 | 6 | 21 | Chem. Eng. J., 2022, 450, 138026. |
| N-RuS_2_/Ru | 120 | 53 | RSC Adv., 2020, 10 (30), 17862-17868. |
| RuIrO_x_ | 12 | 21 | Nat. Commun., 2019, 10, 4875. |
| SL-Ni-Ru-VS_2_ | 20 | 34 | Small, 2024, 20, 2311217. |
| RuB_2_ | 53 | 66.9 | ACS Energy Lett., 2020, 5(9), 2909-2915. |
| CoRu@NG-3 | 52 | 65 | Electrochim. Acta, 2021, 382, 138337. |
| 0.04-Ru@CN-6 | 30 | 42 | J. Colloid Interface Sci., 2021, 604, 885. |
| RuSe_2_/CNTs-650 | 64 | 42.04 | Nanoscale, 2022, 14 (3), 790-796. |
| RuCu NSs/C‐250 °C | 19 | 27.3 | Angew. Chem. Int. Ed., 2019, 58, 13983-13988. |
| Ru/NG-750 | 54 | 44 | ACS Appl. Mater. Inter., 2017, 9, 3785. |
| Co_5_Ru_1_@NCNT/PF | 45 | 64 | Adv. Sci., 2022, 9 (15), 2200010. |
| 29.1 wt% Rh/SiNW | 110 | 24 | Nat. Commun., 2016, 7, 12272. |
| Ru/RuS_2_-2 | 45 | 24.4 | Angew. Chem. Int. Ed., 2021, 60 (22), 12328-12334. |

**Table S10** Fitting parameters of the in-situ EIS curves for Ru@*m*-ZrO_2_/C in 1 M KOH

| E (mV) | R_s_ | CPE1-T | CPE1-P | R_1_ | CPE2-T | CPE2-P | R_2_ |
| --- | --- | --- | --- | --- | --- | --- | --- |
| 0 | 9.424 | 0.007280 | 0.67315 | 112.9 | 0.003697 | 1.056 | 3791 |
| -5 | 9.541 | 0.007418 | 0.65769 | 119.5 | 0.004553 | 1.071 | 578.0 |
| -10 | 9.703 | 0.005467 | 0.72207 | 59.58 | 0.008862 | 0.9477 | 179.5 |
| -15 | 10.05 | 0.005271 | 0.72484 | 51.60 | 0.013146 | 1.011 | 56.09 |
| -20 | 10.74 | 0.005288 | 0.71446 | 48.62 | 0.028468 | 1.147 | 15.64 |

**Table S11** Fitting parameters of the in-situ EIS curves for Ru@*t*-ZrO_2_/C in 1 M KOH.

| E (mV) | R_s_ | CPE1-T | CPE1-P | R_1_ | CPE2-T | CPE2-P | R_2_ |
| --- | --- | --- | --- | --- | --- | --- | --- |
| 0 | 10.66 | 0.007368 | 0.63552 | 36.38 | 0.011858 | 1.015 | 6643 |
| -5 | 10.42 | 0.010371 | 0.54921 | 50.17 | 0.012939 | 1.050 | 1161 |
| -10 | 12.71 | 0.009088 | 0.51019 | 87.34 | 0.020134 | 1.130 | 120.2 |
| -15 | 13.56 | 0.005805 | 0.60386 | 58.63 | 0.065626 | 0.884 | 44.74 |
| -20 | 12.85 | 0.004999 | 0.62712 | 55.25 | 0.32741 | 1.075 | 14.29 |

**Table S12** Fitting parameters of the in-situ EIS curves for Ru@*m*-ZrO_2_/C in 0.5 M H_2_SO_4_.

| E (mV) | R_s_ | CPE1-T | CPE1-P | R_1_ | CPE2-T | CPE2-P | R_2_ |
| --- | --- | --- | --- | --- | --- | --- | --- |
| 0 | 9.302 | 0.004856 | 0.77435 | 192.0 | 0.005734 | 0.93836 | 8446 |
| -10 | 9.004 | 0.004476 | 1.1970 | 34.25 | 0.004817 | 0.70121 | 3952 |
| -20 | 8.751 | 0.003344 | 1.1400 | 37.23 | 0.006732 | 0.63067 | 1471 |
| -30 | 8.871 | 0.003202 | 1.1700 | 22.53 | 0.006343 | 0.61964 | 212.3 |
| -40 | 8.542 | 0.034723 | 0.35279 | 23.08 | 0.002598 | 0.85975 | 71.99 |

**Table S13** Fitting parameters of the in-situ EIS curves for Ru@*t*-ZrO_2_/C in 0.5 M H_2_SO_4_.

| E (mV) | R_s_ | CPE1-T | CPE1-P | R_1_ | CPE2-T | CPE2-P | R_2_ |
| --- | --- | --- | --- | --- | --- | --- | --- |
| 0 | 11.76 | 0.0066586 | 1.164 | 55.94 | 0.0043886 | 0.75457 | 16974 |
| -10 | 11.28 | 0.0045404 | 1.117 | 73.52 | 0.0056266 | 0.70275 | 7953.8 |
| -20 | 11.25 | 0.0034961 | 1.083 | 71.51 | 0.0079477 | 0.63686 | 1724.0 |
| -30 | 11.36 | 0.0033525 | 1.117 | 43.82 | 0.0073954 | 0.63206 | 217.40 |
| -40 | 9.114 | 0.0026221 | 0.86836 | 102.5 | 0.095085 | 0.12573 | 89.520 |

**Table S14** Comparison of the HER activity of the Ru@*m*-ZrO_2_/C with other reported catalysts in alkaline simulated seawater.

| Catalysts | Electrolyte | Overpotential (mV) | Reference |
| --- | --- | --- | --- |
| Ru@*m*-ZrO_2_/C | 1 M KOH + 2 M NaCl | 42@ 10 mA cm^-2^  129@ 100 mA cm^-2^ | This work |
| Ru@*t*-ZrO_2_/C | 1 M KOH + 2 M NaCl | 47@ 10 mA cm^-2^  159@ 100 mA cm^-2^ | This work |
| 20% Pt/C | 1 M KOH + 2 M NaCl | 53@ 10 mA cm^-2^  182@ 100 mA cm^-2^ | This work |
| 5% Ru/C | 1 M KOH + 2 M NaCl | 68@ 10 mA cm^-2^  239@ 100 mA cm^-2^ | This work |
| Ru@MoC | 1 M KOH + 0.5 M NaCl | 58@ 10 mA cm^-2^ | Angew. Chem. Int. Ed., 2025, 60(30), e202505031. |
| Ni@O-C/CNTs | 1 M KOH + 0.5 M NaCl | 47@ 10 mA cm^-2^ | The Innovation Materials, 2025, 3(3), 100148. |
| Ti/TiO_2_@NiBx (PEE) | 1 M KOH + 0.5 M NaCl | 91@ 10 mA cm^-2^ | Chem. Eng. J. 2022, 430, 132881. |
| Ru@*m*-ZrO_2_/C | 1 M KOH + simulate seawater | 158@ 100 mA cm^-2^ | This work |
| Ru@*t*-ZrO_2_/C | 1 M KOH + simulate seawater | 199@ 100 mA cm^-2^ | This work |
| 20% Pt/C | 1 M KOH + simulate seawater | 291@ 100 mA cm^-2^ | This work |
| 5% Ru/C | 1 M KOH + simulate seawater | 274@ 100 mA cm^-2^ | This work |
| Co@RuCo-3 | 1 M KOH + seawater | 161@ 100 mA cm^-2^ | Appl. Catal. B Environ., 2022, 315, 121554. |
| Ni_2_P-Fe_2_P/NF | 1 M KOH + seawater | 135@ 10 mA cm^-2^ | Adv. Funct. Mater., 2020, 31, 2006484. |
| Co-MoSe_2_ | 1 M KOH + seawater | 190@ 100 mA cm^-2^ | ACS Appl. Mater. Interfaces, 2022, 14, 10246. |
| Ru/B-CoP | 1 M KOH + seawater | 82@ 10 mA cm^-2^ | Chem. Eng. J., 2023, 452, 139175. |
| S-NiMoO_4_@NiFe-  LDH/NF | 1 M KOH + seawater | 220@ 100 mA cm^-2^ | J. Colloid Interface Sci., 2022, 613, 349–358 |
| Ni_2_P-Fe_2_P | 1 M KOH + seawater | 252@ 100 mA cm^-2^ | Adv. Funct. Mater., 2021, 31(1): 2006484. |
| Mn-MoWNi | 1 M KOH + seawater | 261@ 100 mA cm^-2^ | Fuel, 2024, 372, 132281. |
| NC-Ni_3_N21 | 1 M KOH + seawater | 290@ 100 mA cm^-2^ | ACS Sustain. Chem. Eng., 2023, 11(17): 6556-6566. |
| NiCoHPi@Ni_3_N/NF | 1 M KOH + seawater | 182@ 100 mA cm^-2^ | ACS Appl. Mater. Interfaces, 2022, 14, 22061-22070. |

**Table S15** Comparison of the AEMWE device performance of Ru@*m*-ZrO_2_/C || NiFe-LDH and recently-reported AEMWE devices in 1 M KOH.

| AEMWE devices | Operation temperature (°C) | Cell voltage (V) | Reference |
| --- | --- | --- | --- |
| Ru@*m*-ZrO_2_/C \|\| NiFe-LDH//NF | 60 | 1.64@0.5 A cm^-2^  1.76@1 A cm^-2^  1.98@2 A cm^-2^ | This work |
| Ru_SA/NP_-PNCFs \|\| NiFe-LDH | 20 | 1.77@0.5 A cm^-2^ | Joule, 2024, 8, 1790-1803. |
| UP-RuNiSAs/C \|\| NiFeO_x_ | 70 | 1.70@0.5 A cm^-2^  1.95@1 A cm^-2^ | Nat. Commun., 2024, 15, 2218. |
| SL-Pt cluster \|\| IrO_2_ | / | 1.59@0.5 A cm^-2^  1.74@1 A cm^-2^ | Adv. Funct. Mater., 2022, 33, 2212752. |
| Li_3.0_RuSn_0.8_ NWs/C \|\| NiFeO_x_H_y_ | 80 | 1.689@1 A cm^-2^ | J. Am. Chem. Soc., 2025, 147, 7711-7720. |
| Ru/Ni-N4C-300 \|\| NiFe-LDH | 50 | 1.72@0.5 A cm^-2^ | Adv. Funct. Mater., 2025, 35, 2416071. |
| Ru-YNC \|\| NiFe-LDH | 80 | 1.78@0.5 A cm^-2^  1.87@1 A cm^-2^ | Energy Environ. Sci., 2025, 18(12), 6141-6153. |
| Pt/C_com_ \|\| NiFe-LDH | 80 | 1.90@0.5 A cm^-2^ | Energy Environ. Sci., 2025, 18(12), 6141-6153. |
| Ru/C_com_ \|\| NiFe-LDH | 80 | 2.03@0.5 A cm^-2^ | Energy Environ. Sci., 2025, 18(12), 6141-6153. |
| Pt_1_+n/Ni_3_S_2_ \|\| RuO_2_ | 80 | 2.00@0.5 A cm^-2^ | Appl. Catal. B Environ., 2024, 354, 124074. |
| Pt/NiCoP@MXene \|\| NiFe-LDH | 80 | 1.74@0.5 A cm^-2^  1.80@1 A cm^-2^ | Angew. Chem., Int. Ed., 2024, 63, e202401819. |
| Fe_2_P-Co_2_P ǀǀ NPC NiFe(OH)_x_-Ni_3_S_2_ | 60 | 1.73@1 A cm^-2^ | ACS Nano, 2023, 17, 24070. |
| Mn-O-Ru \|\| RuO_2_ | 25 | 1.97@0.25 A cm^-2^ 2.36@0.5 A cm^-2^ | Adv. Mater., 2023, 35, 2303331. |
| NA-Ru_3_Ni/C \|\| NA-Ru_3_Ni/C | 60 | 1.82@0.5 A cm^-2^ 2.05@1 A cm^-2^ | Energy Environ. Sci., 2023, 16, 285-294. |
| Ru-LC-Ni(OH)_2_ \|\| NiFe-LDH | 80 | 1.65@0.5 A cm^-2^ 1.69@1 A cm^-2^ | Angew. Chem., Int. Ed., 2024, 136(7), e202317220. |
| PtPd-HEA \|\| NiFe-LDH | 80 | 1.81@1 A cm^-2^ | ACS Catal., 2025, 15, 11022-11033. |
| Pt/CoMoO_4_ \|\| NiFe-LDH | R.T. | 1.63@1 A cm^-2^ | Adv. Funct. Mater., 2025, 2423537. |
| PtRu/C \|\| IrO_2_ | 60 | 2.00@1.75 A cm^-2^ | Electrochem. Soc., 2021, 168, 054522. |
| NiCu MMO \|\| Ir | 50 | 2.00@1.85 A cm^-2^ | Electrochim. Acta, 2021, 371, 137837. |
| Ru_1_-Mo_2_C ǀǀ NiFe LDH | 65 | 1.83@1.0 A cm^-2^  2.03@2.0 A cm^-2^ | Energy Environ. Sci., 2024, 17, 1397. |
| CoNiS \|\| CoNiS | 60 | 2.00@2.2 A cm^-2^ | Nat. Commun., 2022, 13, 7956. |
